# Evaluation of the HOPE spiritual assessment model: a scoping review of international interest, applications and studies over 20+ years

**DOI:** 10.1186/s12904-025-01809-z

**Published:** 2025-07-07

**Authors:** Georgia Sleeth, Priya Gottlieb, Achutha Srinivasan, Ufuoma Thaddeus, Meera Mennillo, Gowri Anandarajah

**Affiliations:** 1 University of Washington School of Medicine, Seattle, WA USA; 2 Boston University, Boston, MA USA; 3 The Warren Alpert Medical School of Brown University, Providence, RI USA; 4 University of Rhode Island, Kingston, RI USA; 5 Family Medicine and Medical Science, The Warren Alpert Medical School of Brown University, 222 Richmond Street, Providence, RI USA

**Keywords:** Spirituality, Religion and medicine, Comprehensive healthcare, Communication, Spiritual assessment, Spiritual history, Palliative care

## Abstract

**Background:**

Evidence supports classifying spiritual health as a determinant of health and including spiritual care in comprehensive patient-centered care. Despite delineation of primary versus specialty palliative skills, including spiritual care, and availability of spiritual history/assessment communication tools designed for non-specialist (SH/SAs), medical teams continue to neglect patients’ spiritual needs. A possible contributor is that consolidated evidence regarding uses and/or effectiveness of these SH/SA tools is lacking.

**Aim:**

To explore interest, applications and evaluations of one of the well-known SH/SA tools - the HOPE spiritual assessment.

**Methods:**

We conducted a scoping review following Arksey and O’Malley’s protocol and PRISMA Extension for Scoping Reviews (PRISMA-ScR). We searched PubMed, Web-of-Science, Google Scholar, PsycInfo, Academic Search Premier, CINAHL, Atla Religion Database, with AtlaSerials and SocIndex, for all sources citing the original 2001 HOPE article (to July 2023); no restrictions on article type, location, language. We used tiered inclusion/exclusion criteria, corresponding to our specific research questions regarding interest, applications and evaluations of HOPE.

**Results:**

Of 1,047 unique sources, 909 underwent full-text review. 571 explicitly mentioned/cited HOPE, representing 51 countries, 21 languages, and multiple disciplines including: 55% medicine, 15% nursing, 7.5% psychology, 6% chaplaincy, 5% social work. 266 sources offered expert opinions about HOPE. 63 described specific experience using and/or evaluating HOPE; 17 from non-English speaking countries. 59 demonstrated acceptability, 34 feasibility, 30 content validity. Of the 31 formal studies/evaluations, 17 intervention studies of HOPE demonstrated validity as a clinical, educational, or qualitative research tool, and 14 studies analyzed the HOPE model itself, with 10 comparing SH/SA tools. In these comparisons, HOPE rated highly, as did some others. HOPE’s comparative strengths include: acceptability for diverse (secular/religious/multicultural) populations; adaptability across clinical settings; flexibility for use by novice and expert clinicians.

**Conclusion:**

This first systematically constructed review of any of the well-known SH/SA tools revealed broad, international interest in HOPE and evidence for its acceptability, feasibility, and validity in diverse settings. Next steps for improving patient-centered spiritual care include: disseminating evidence; clarifying spiritual care competencies/boundaries for different disciplines/settings; increasing required primary spiritual care training; increasing availability of spiritual care specialists; and improving clinical systems to support whole-person care.

**Supplementary Information:**

The online version contains supplementary material available at 10.1186/s12904-025-01809-z.

## Introduction

### Rationale

Over the last 50 years increasing evidence indicates that spiritual care is an important, yet often neglected, aspect of whole-person care, relevant not only in the hospice/palliative setting but in numerous other clinical settings [1–8]. The adoption of a biopsychosocial-spiritual approach to patient care [9, 10] has recently been further supported by public health leaders communicating that evidence is now robust enough to consider spirituality to be a determinant of health [1, 11]. 

Currently major healthcare organizations and accreditation bodies worldwide require that patients’ spiritual needs be addressed [12–16]. Similarly, medical education accrediting agencies and clinical practice guidelines have developed core spiritual care competencies for various specialities [17–21]. 

Furthermore, the clarification of primary palliative care versus specialty palliative care, including spiritual care, has delineated skills needed by all clinicians and those needed by specialists [22]. For patients to receive needed spiritual care, they need assistance from both primary and specialty spiritual care clinicians [23–25]. 

Early studies exploring the mismatch between patients’ desire for and physicians’ provision of spiritual care, revealed that major physician barriers include lack of an inclusive definition of spirituality, lack of training, and lack of time [26–31]. Consequently, several widely accepted definitions have emerged, as have many training tools [32–34]. 

Amongst these clinical training tools are several spiritual history and spiritual assessment models designed for primary spiritual care [33].– [34] The most well-known [33]– [34] include FICA [35], HOPE [36], SPIRIT [37], FAITH [38], CSI-Memo [39]. These differ from spiritual assessments designed for clinical chaplains (e.g. Fichett’s 7 × 7) [40] in that they do not provide the depth of assessment needed by spiritual care specialists. They also differ from spiritual assessment instruments designed for quantitative research (e.g. FACIT-SP-12) [41] since their goal is to enhance communication regarding patients’ spiritual needs during routine medical care, rather than quantify religious/spiritual domains for research. Even though these tools are clinical communication tools, not research instruments, some authors have voiced concerns that most primary spiritual care history/assessment (SH/SA) tools are not validated (e.g. Luccetti 2013) [33]. 

Despite increased awareness of patients’ spiritual care needs and availability of communication tools, recent studies reveal a persistent mismatch between medical teams’ provision of and patients’ desire for spiritual care [42–44]. While most physicians, nurses and other clinicians support the inclusion of spiritual care in medical care, they remain reluctant to inquire about patients’ spiritual needs [42–44]. One hypothesis for a contributor to this continued mismatch is lack of consolidated evidence regarding the acceptability, feasibility and/or validity of utilizing any of these communication tools.

### Goals of this scoping review

Given the paucity of information regarding uses of primary spiritual care history/assessment tools, the purpose of this scoping review is to broadly explore ways in which one well-known spiritual history/assessment tool, the HOPE spiritual assessment model, has been used and/or studied since first published in 2001. Review results may elucidate next steps in improving spiritual care provision during routine medical care.

To provide context for this review, below are details regarding: (1) definitions of spirituality for clinical settings; (2) definitions of spiritual history and spiritual assessment; (3) the HOPE model.

### Inclusive definition of spirituality

Several authors offer inclusive definitions of spirituality addressing needs of a variety of patients. Puchalski’s [45] consensus definition states: *“Spirituality is the aspect of humanity that refers to the way individuals seek and express meaning and purpose and the way they experience their connectedness to the moment*,* to self*,* to others*,* to nature*,* and to the significant or sacred.”*

Based in literature review, the 3H model clarifies the multidimensional nature of spirituality as encompassing existential/cognitive, experiential/emotional and behavioral aspects [10, 36], while the BMSEST models explore cross-cultural understanding of spirituality [10]. 

Spiritual pain, suffering or distress, can occur when: *“individuals are unable to find sources of meaning*,* hope*,* love*,* peace*,* comfort*,* strength and connection in life or when conflict occurs between their beliefs and what is happening in their life.”* [36].

### Spiritual history/assessment models

While recent palliative care consensus meetings have recommended the term ‘spiritual history’ for discussions conducted by primary spiritual care clinicians and ‘spiritual assessment’ for those by spiritual care specialists [46], there are many clinical settings (e.g. primary care) where clinical chaplains are not readily available. In these settings, clinicians, by necessity, have to make preliminary assessments regarding patients’ spiritual needs and create therapeutic plans [36, 47–49], based on patient-centered communication and shared decision making [50–52]. These might include simple modifications in treatment plans (e.g. modifying insulin regimen during Ramadan) or referral to a spiritual care specialist (e.g. community resource or clinical chaplain).

Given these different sets of needs based on setting and availability of spiritual care specialists, the term *‘spiritual history/assessment’* [SH/SA] will be used in this manuscript for communication models designed for primary spiritual care clinicians.

### The HOPE model

The HOPE Model for Spiritual Assessment (Table 1) is a spiritual history/assessment approach developed in the 1990s by Gowri Anandarajah MD as a communication training tool for family medicine residents and medical students. After being refined over several years during teaching sessions and by incorporating feedback from diverse patients and learners, it was first published in 2001 in the journal American Family Physician [36], and thus made readily available for anyone who wished to apply this practical tool. The HOPE communication approach facilitates patient-centered explorations of patients’ spiritual resources and needs during medical care. As a flexible model, it can be used by novice clinicians to gather basic history and also by seasoned clinicians to assist in shared decision making [50–52] with patients.

The HOPE model [36] provides 4 domains of inquiry/discussion, beginning with “**H**”: Sources of **H**ope, meaning, comfort, strength, peace, love and connection. This open-ended exploration of spiritual resources allows for conversations with a variety of patients, including those who do not identify as religious/spiritual, those with minority religious backgrounds, or those who have been hurt because of or by religion. Depending on how the H domain conversation goes, the clinician may proceed to “**O**” and “**P**” domains, which explore the patient’s relationship with **O**rganized religion and the **P**ersonal spiritual **p**ractices most meaningful to them. Finally, the “**E**” domain focuses on the **E**ffects of the patient’s spiritual beliefs/needs on their medical care and/or **E**nd-of-life decisions/care. This domain provides experienced clinicians the opportunity to utilize their assessment and shared decision-making skills to best meet their patients’ needs [50–52]. 

For each domain, the HOPE model provides example questions and normalizing statements to facilitate discussion. Clinicians may utilize, modify or omit questions based on the situation. They may also change the order of the questions and/or domains, following the opening question, based on the natural flow of the conversation. This “toolbox” approach allows clinicians to tailor their discussions to patients’ needs (Table 1).

**Table 1:** The HOPE model for spiritual assessment

Domain	Examples of Questions and Transition Statements	Comments from this Scoping Review's Sources**
**H**	(Sources of) **h**ope, meaning, comfort, strength, peace, love and connection	
	*Transition Statement*: *“We have been discussing your support systems. I was wondering*,*…” OR* “It sounds like you have been going through a very difficult time in your life. I was wondering…”* *Example Questions*: • What are your sources of hope, strength, comfort and peace? * • What do you hold on to during difficult times? * • What sustains you and keeps you going? • What is there in your life that gives you internal support? *Transition Statement*: *“For some people*, their religious or spiritual beliefs act as a source of comfort and strength in dealing with life’s ups and downs; is this true for you?”* • If the answer is “Yes,” go on to O and P questions. • If the answer is “No,” go to E *or* consider asking: Was it ever? If the answer is “Yes,” ask: What changed?	• Transition statements clarify purpose and facilitate acceptability of these questions • Starting with general spiritual themes, rather than words such as religion or spirituality, facilitate conversations with diverse and/or secular populations, and with those who might be estranged from their religion or faith community. • Clinicians might find that patients answer O & P questions spontaneously at this stage and organic conversation is preferable to following the HOPE format. • Seasoned clinicians *might* choose to ask the “What changed?” question to probe for possible past/present spiritual pain.
**O**	**O**rganized religion	
	*Example Questions*: • Do you consider yourself part of an organized religion? * • How important is this to you? • What aspects of your religion are helpful and not so helpful to you? • Are you part of a religious or spiritual community? Does it help you? How?	• This domain explores the role of religion and religious community in a person’s life. • It opens the door for exploration of both positive and negative experiences by seasoned clinicians.
**P**	**P**ersonal spirituality/ **P**ractices	
	*Example Questions*: • Do you have personal spiritual beliefs that are independent of organized religion? What are they? * • Do you believe in God (or the Transcendent)? What kind of relationship do you have with God? • What aspects of your spirituality or spiritual practices do you find most helpful to you personally? (e.g., prayer, meditation, reading scripture, attending religious services, listening to music, hiking, communing with nature)*	• This domain takes a strengths-based, patient-centered approach to exploring spiritual practices that might be of benefit to the patient • It acknowledges both religious and secular approaches to spiritual health and wellbeing
**E**	**E**ffects on medical care and **E**nd-of-life issues	
	*Example Questions*: • Has being sick (or your current situation) affected your ability to do the things that usually help you spiritually? (Or affected your relationship with God?) • As a doctor, is there anything that I can do to help you access the resources that usually help you? • Are you worried about any conflicts between your beliefs and your medical situation/care/decisions? * • Would it be helpful for you to speak to a clinical chaplain/community spiritual leader? * • Are there any specific practices or restrictions I should know about in providing your medical care? (e.g., dietary restrictions, use of blood products)* *If the patient has a terminal diagnosis/is dying*: • How do your beliefs affect the kind of medical care you would like me to provide over the next few days/weeks/months?	• The “E” domain can be used for continued spiritual history taking **or** for seasoned clinicians an opportunity for assessment of spiritual needs and shared decision making regarding the clinical care plan. • Together with insights gathered from the other domains, a non-specialist in spiritual care can use this domain to identify who might benefit from a referral to a spiritual care specialist. • A seasoned clinician might explore if anything has changed to probe for spiritual distress

*Most commonly cited or highly rated questions, based on findings from this scoping review

** Practical notes for each HOPE domain– derived from themes from comments in this scoping review's sources in which authors offered thoughts about using HOPE (see Table 3)

**Table 2:** Demographics

	Sources that Specifically Mention HOPE in the Text (with or without other SH/SA tools) (*N* = 571)	Sources that Include Some Evaluation of HOPE (*N* = 266)
• COUNTRIES	***N*** = 571	***N*** = 266
**USA**	305 (53.4%)	147 (55.3%)
**Canada**	17 (3.0%)	8 (3.0%)
**South & Central America** (Brazil, Chile, Colombia, Costa Rica, Ecuador, Mexico, Uruguay)	35 (6.1%)	13 (4.9%)
**Africa** (Democratic Republic of the Congo, Kenya, Libya, Nigeria, South Africa)	10 (1.8%)	5 (1.9%)
**United Kingdom (**England, Scotland, Wales, Northern Ireland)	62 (10.9%)	29 (10.9%)
**Northern Europe** (Denmark, Finland, Norway, Sweden)	7 (1.2%)	6 (2.3%)
**Western Europe** (Austria, Belgium, France, Germany, Netherlands, Switzerland)	42 (7.4%)	19 (7.1%)
**Eastern Europe** (Croatia, Czech Republic, Hungary, Lithuania, Poland, Slovenia)	15 (2.6%)	9 (3.4%)
**Southern Europe** (Greece, Italy, Malta, Portugal, Spain)	19 (3.3%)	6 (2.3%)
**Middle East** (Iran, Lebanon, Turkey)	8 (1.4%)	5 (1.9%)
**Asia** (China, India, Korea, Malaysia, Philippines, Singapore, Thailand, Hong Kong)	21 (3.7%)	6 (2.3%)
**Oceania** (Australia, Fiji, New Zealand)	30 (5.3%)	13 (4.9%)
**• LANGUAGES**	***N*** = 571	***N*** = 266
**ENGLISH**	**477 (83.5%)**	**225 (84.6%)**
**NON-ENGLISH**	**94 (16.5%)**	**41 (15.4%)**
Non-English Language Breakdown	*N* = 94	*N* = 41
Chinese	1 (1.0%)	0 (0.0%)
Croatian	2 (2.1%)	1 (2.4%)
Czech	2 (2.1%)	1 (2.4%)
Dutch	5 (5.3%)	3 (7.3%)
Finnish	2 (2.2%)	2 (4.9%)
French	9 (9.6%)	4 (9.8%)
German	14 (14.9%)	5 (12.2%)
Greek	1 (1.1%)	0 (0.0%)
Hungarian	1 (1.0%)	1 (2.4%)
Korean	1 (1.0%)	0 (0.0%)
Malay	1 (1.0%)	1 (2.4%)
Norwegian	1 (1.1%)	0 (0.0%)
Polish	4 (4.3%)	4 (9.8%)
Portuguese	22 (23.4%)	10 (24.4%)
Slovenian	4 (4.3%)	1 (2.4%)
Spanish	18 (19.1%)	4 (9.8%)
Swedish	2 (2.2%)	2 (4.9%)
Thai	1 (1.1%)	0 (0.0%)
Nigerian	1 (1.1%)	1 (2.4%)
Turkish	2 (2.2%)	1 (2.4%)
**• MAJOR DISCIPLINES**	***N*** = 571	***N*** = 266
Medicine	313(54.8%)	144 (54.1%)
Nursing	88 (15.4%)	50 (18.8%)
Psychology	43 (7.5%)	17 (6.4%)
Chaplaincy	35 (6.1%)	17 (6.4%)
Healthcare (unspecified)	32 (5.6%)	9 (3.4%)
Social Work	28 (4.9%)	15 (5.6%)
Occupational Therapy	7 (1.2%)	3 (1.1%)
Genetic Counseling	7 (1.2%)	4 (1.5%)
Other (pharmacology, public health, sociology, law, military, music therapy, business)	18 (3.2%)	7 (2.6%)
**• MEDICINE SUBSPECIALTIES**	***N*** = 313	***N*** = 144
Palliative Medicine	82 (26.2%)	30 (20.8%)
Psychiatry	45 (14.4%)	20 (13.9%)
Family Medicine	41 (13.1%)	24 (16.7%)
Oncology	34 (10.9%)	8 (5.6%)
Internal Medicine	25 (8.0%)	11 (7.6%)
Medical Subspecialities	17 (5.4%)	8 (5.6%)
Pediatrics	7 (2.2%)	3 (2.1%)
Gynecology	5 (1.6%)	4 (2.8%)
Surgery	6 (1.9%)	3 (2.1%)
Emergency Medicine	6 (1.9%)	4 (2.8%)
Rehabilitation	5 (1.6%)	3 (2.1%)
Unspecified	40 (12.8%)	26 (18.1%)

**Table 3:** Qualitative analysis of comments from sources regarding strengths and weaknesses of HOPE and other spiritual history/assessment (SH/SA) tools (*N* = 266 sources)

Themes	Subthemes	Representative Quotes
Most Common SH/SA Tools Mentioned	FICA, HOPE, SPIRIT	
**All SH/SA Tools**: **General Strengths**	1. Short, structured, easy to use by non-specialists in spiritual care 2. Enhances patient-centered care 3. Enables further conversations about spiritual care	“Mnemonics make these three spiritual assessment tools easy to remember and integrate a spiritual assessment into a holistic assessment.” (Chrash M. et al., 2011) “The availability of spiritual history-taking tools… provides valuable assistance in approaching this vital aspect of holistic care at the end of life, making it accessible to everyday care providers.” (Blaber, 2015) “these published instruments can guide the obtaining of spiritual history and facilitate the approach by doctors who do not yet have experience with this practice.” (Abuchaim, 2018) Typically, they are designed to minimise barriers to conversation by having open-ended questions that allow a person room to express their spiritual and religious understandings without feeling confined by expectation or judgment. (Grant, 2007)
**All SH/SA Tools**: **General Weaknesses**	1. Most lack cultural diversity; center Judeo-Christian beliefs 2. Require a conversation; difficult to integrate into clinic workflow 3. Perception that tools are not validated	“Some of the tools explored more abstract possibilities such as if the respondent considered themselves religious or spiritual. The limitation across these inquiries was their focus on what is outright religious.” (Bond & Brown, 2020) “It should be remembered that these tools require a conversation with people, as they are based on a dialogue. For those unable or unwilling to converse, these tools will have some limitations.” (Haire, 2010) “All these history-taking tools [HOPE, FICA, SPIRIT] are strikingly similar, even though they have all been developed independently. However, none has undergone any serious psychometric testing. The questions are relevant to understanding the lives and spiritual needs of patients, and one might argue that this sort of testing is no more required than it is required to validate how to ask questions about past medical history, occupation, sexual practices, and hobbies. Still, having valid and predictive instruments for clinicians would be a useful field of study.” (Sulmasy, 2002)
**The HOPE Model**: **Relative Strengths** (compared to other SH/SAs)*	1. Acceptable for diverse (secular, religious, multicultural) settings 2. Normalizing and unassuming language (e.g. sources of hope, strength) 3. Flexible communication tool (for novice and experienced clinicians) 4. Strength-based approach 5. Asks if anything changed	“The HOPE questions…allow for open-ended exploration of the patient’s general spiritual resources and concerns, and it serves as a natural follow-up to discussions of other systems of medical care. It does not immediately focus on the words “spirituality” or “religion”. This is thought to minimize barriers to discussion based on the use of language.” (Larson, 2003) “The HOPE questionnaire is useful because it is relatively brief, it is patient-centered and introduces the topic gradually, and it is respectful of the beliefs of most religious or spiritual traditions.” (Koenig, 2001) “The HOPE tool provides normalizing sentences to assist the healthcare worker when addressing ‘religious’ and ‘spiritual’ beliefs, using the phrase *‘for some people… is this true for you?*’ A patient may perceive such a phrase as less threatening and intrusive, facilitating communication.” (Blaber, 2015) “it provides both a clear structure for novice or uncomfortable practitioners, as well as a flexible and open approach for more experienced practitioners [GPs]”. (Whitehead, 2022) “The brief HOPE assessment tool itself becomes a therapeutic intervention whereby practitioners offer their presence, understanding, acceptance, and compassion when assessing spirituality.” (Kretzer, 2005) “It is worth emphasizing that it is the only questionnaire that seeks to know whether the disease interfered/altered the patient’s belief. " (Queiroz, 2022) “Pastors can benefit from understanding even a basic spiritual assessment tool such as the HOPE tool. The Hope Assessment questionnaire is brief, making it useful in time-limited situations. It is also one of the less intrusive initial assessments, sensitive to a wide range of belief systems and cultures.” (Robinson, 2012)
**The HOPE Model**: **Relative Weaknesses** **(compared to other SH/SAs)**	1. Perception of too many questions 2. Requires having a conversation 3. Perception that it has not been validated as a research tool	“The HOPE tool was criticized for its length [by some study participants]…. People who were comfortable addressing the topic with their existing consultation skills felt that tools such as HOPE can be too constraining and disrupt the flow of a consultation… [However] the HOPE tool is designed as a tool and framework and should be used flexibly according to patient cues and the demands of the consultation. (Whitehead, 2022) “The HOPE survey has not been validated as a research survey, but it allows an initial approach to the issues of spirituality and religion.” (Canteros, 2021)
Most Commonly Used/Recommended HOPE Questions by Domain*	1. H 2. O 3. P 4. E	H Domain** • *“It sounds like you have been going through a very difficult time in your life. I was wondering…”* - What are your sources of hope, strength, comfort and peace? - What do you hold on to during difficult times? • *“For some people*,* their religious or spiritual beliefs act as a source of comfort and strength in dealing with life’s ups and downs; is this true for you?”* *O Domain* • Do you consider yourself part of an organized religion? *P Domain* • Do you have personal spiritual beliefs that are independent of organized religion? What are they? • What aspects of your spirituality or spiritual practices do you find most helpful to you personally? (e.g., prayer, meditation, reading scripture, attending religious services, listening to music, hiking, communing with nature) E Domain** • Are there any specific practices or restrictions I should know about in providing your medical care? (e.g., dietary restrictions, use of blood products) • Are you worried about any conflicts between your beliefs and your medical situation/care/decisions? • Would it be helpful for you to speak to a clinical chaplain/community spiritual leader?

See Supplemental Table 1 for all 266 references used for this analysis and Table 2 for demographics of this subset of 266 sources

*See Table 1 for complete HOPE model plus relative strengths by domain and most commonly used questions (based on this qualitative analysis)

**H and E domains were the most often used domains for short screening adaptations of HOPE

## Methods

We selected a scoping review methodology to address the broad aims of this study. This review followed the 5-stage recommendations outlined by Arksey and O’Malley [53] with enhancements by Levac [54]. The PRISMA Extension for Scoping Reviews (PRISMA-ScR) [55] was used to guide the reporting process.

### Stage 1: identifying the research questions

The following specific research questions addressed our overarching study goal: (1) What is the extent and nature of interest in the HOPE model (disciplines, countries, languages, etc.)? (2) How has HOPE been used/adapted for different settings? (3) What studies/evaluations exist regarding the HOPE model itself or applications of HOPE? (4) What are strengths/weaknesses of HOPE? (5) What evidence exists regarding acceptability, feasibility and/or validity of HOPE?

### Stage 2: identifying relevant literature

A preliminary literature search aimed at identifying articles discussing the HOPE model revealed several challenges to capturing all relevant articles. These included: the very common word “hope”; the fact the HOPE model is referred to in various ways including “HOPE spiritual assessment”, “HOPE tool”, “HOPE model,” “HOPE spiritual history”, or often simply “HOPE”; and that often HOPE was not included in title or abstract, but rather appeared in other manuscript sections (e.g. methods).

Given these challenges, after consulting with a university medical librarian, the research team decided to undergo the labor-intensive task of doing full-text reviews of all articles citing the original HOPE article [36], rather than utilizing typical Boolean search strings. The librarian and research team agreed that this would yield the most comprehensive set of articles for review.

The following databases were searched for articles citing the original 2001 HOPE article [36] from publication until July 6, 2023: PubMed, Web-of-Science, Google Scholar, PsycInfo, Academic Search Premier, CINAHL, Atla Religion Database, with AtlaSerials and SocIndex. Articles were organized using Zotero [56]. 

### Stage 3: study/source selection

Given the broad aim of this study, researchers adopted a tiered approach to source selection, with different inclusion/exclusion criteria for each progressively narrower tier.

At all stages, at least 2 researchers independently reviewed each article for eligibility.

**Fig. 1:**
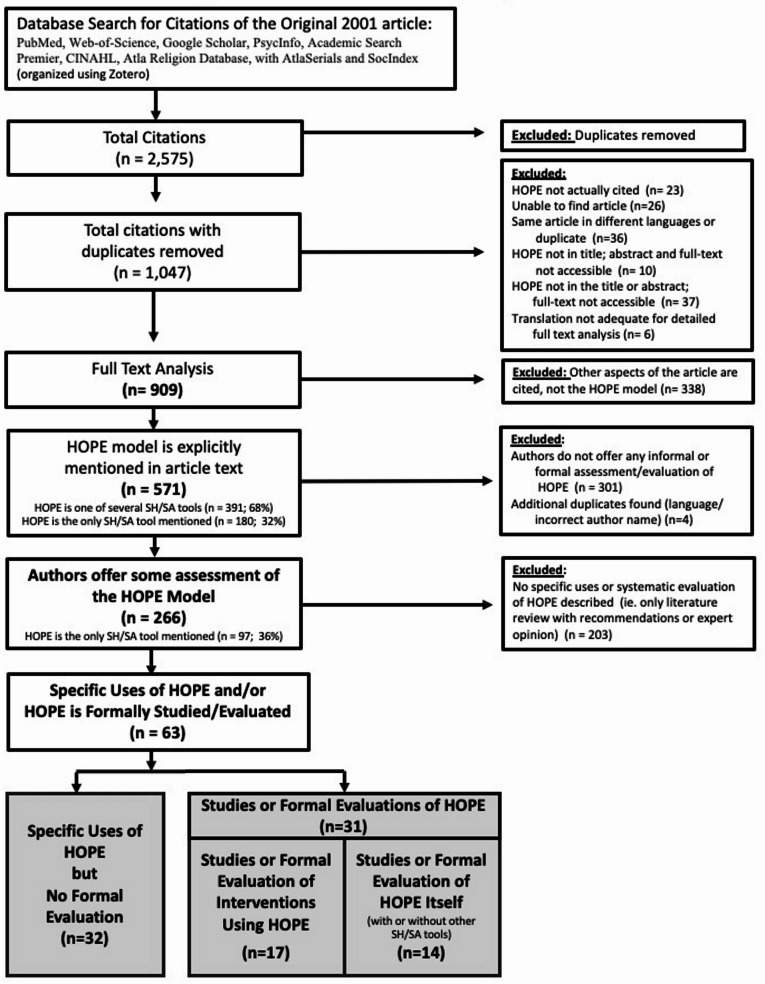
Prisma flow diagram

Given the large number of full-text reviews needed, the research team included 6 researchers. To ensure consistency in selection, a data-extraction questionnaire was developed and converted into an excel data-gathering spreadsheet. Disagreements between reviewers were resolved via discussion and/or input from a third reviewer. Additionally, researchers met regularly to clarify questions and/or resolve inconsistencies. Figure 1 (PRISMA Diagram) illustrates this tiered selection process.

*Tier 1*: After duplicates were removed, two researchers (AS, GA) independently screened all articles and excluded articles ineligible for full-text review. This included: source did not cite the HOPE article; inability to locate article; HOPE was not in title and/or abstract and full-text not available; same article in another language (duplicate); or translation not adequate for even superficial full-text screening.

*Tier 2*: The resulting 909 articles underwent full-text screening by 6 screeners (AS, PG, GA, GS, MM, KS), with each article reviewed by 2 screeners. Articles were excluded if they cited other aspects of the original article (e.g. definitions), but not the HOPE model. At this stage, data extraction included: author, publication-year, publication-type, country, language, whether HOPE was explicitly mentioned/cited in the text, whether multiple spiritual history/assessment tools were mentioned, and whether authors described a specific use of HOPE and/or offered evaluative comments about HOPE.

*Tier 3*: 571 articles explicitly mentioned/cited the HOPE model; in 180(32%) HOPE was the only spiritual history/assessment tool. Next, sources were excluded if authors did not include some assessment of HOPE (e.g. expert opinion, literature-based recommendations, experience using HOPE, formal evaluation/study).

*Tier 4*: The remaining 266 articles were further screened regarding whether authors offered only theoretical opinions or described specific experience using HOPE and/or had formally evaluated/studied HOPE. Those offering only theoretical opinions were excluded.

*Tier 5*: The remaining 63 articles were divided into articles that: evaluated/studied HOPE itself or an intervention using HOPE; or described a specific experience using HOPE, without an explicit evaluation of HOPE.

### Stage 4: charting & analyzing the data

This tiered screening process provided 4 source-subsets. Each underwent detailed data charting and quantitative and/or qualitative analysis corresponding to the specific research questions being addressed.

*Group 1- Sources Explicitly Mentioning HOPE*: These 571 sources provide insights into scope of interest in HOPE. Data of interest included countries, languages and disciplines. Two researchers (PG, GA) charted and organized data (Table 2).

*Group 2 - Sources Offer Some Assessment of HOPE*: These 266 sources (Supplemental Table 1) provide formal and/or informal assessments of HOPE. Many also assessed other spiritual history/assessment tools. 5 reviewers (PG, AS, GS, MM, GA) extracted narrative comments about spiritual history/assessment tools from sources (each source by 2 reviewers). Comments underwent qualitative analysis (Table 3) by 5 researchers (GS, MM, UT, PG, AS). GA, HOPE’s author, abstained from this analysis to minimize bias.

*Group 3 - Specific Uses of HOPE; No Evaluation*: 5 researchers (GS, UT, MM, AS, PG) extracted detailed data from these 32 articles (Table 4), including: author, year, country, language, setting, participants, description of use and outcomes. All 6 researchers (GS, UT, AS, PG, MM, GA) then independently reviewed each article and table accuracy. Disagreements in data interpretation were resolved during group analysis meetings.

*Group 4 - Formal Study/Evaluation of HOPE or Interventions Using HOPE*: For these 31 articles, the same 5 researchers extracted the same data as for group 3 adding study method and outcomes. They also divided studies into those evaluating an intervention utilizing HOPE (*N* = 17; Table 5) and those evaluating the HOPE model itself (*N* = 14; Table 6). All 6 authors then independently reviewed each article and table accuracy, resolving disagreements in analysis meetings.

### Stage 5: collating, summarizing and reporting results

Data synthesis, identifying key findings and elucidating gaps in knowledge were done by all 6 researchers through iterative group analysis meetings. Each group of evidence was reviewed and discussed until consensus was reached.

Consensus regarding evidence pertaining to acceptability, feasibility and validity of the HOPE model was also achieved through group analysis meetings. Since HOPE is a flexible communication tool, not a measurement tool, definitions for validity used for quantitative instruments [57], such as psychometric testing, do not apply [58–60]. 

Epstein and others describe the complexity involved in evaluating communication models in “real-life” settings, which are subject to numerous variables (e.g. patient factors, clinical factors, setting, social context) [61, 62]. Therefore researchers utilized insights regarding assessing validity in qualitative research [59, 60] and communication models (e.g. shared decision making, patient-centered communication) [61, 63] to develop appropriate criteria to apply to sources.

Researchers developed a rubric to ensure consistency in definitions used to assess evidence in each article for: (1) Acceptability; (2) Feasibility; (3) Face Validity; (4) Content Validity; (5) Validity as a Teaching Tool; (6) Validity as a Clinical Tool; (7) Validity as a Qualitative Research Tool; (8) Validity in Different Languages (Supplemental Table 2).

## Results

### Scope of interest in the HOPE model and other spiritual history/assessment tools

Of the 571 sources explicitly mentioning/citing HOPE, 68% also mention other similar tools. Therefore, this data-subset provides insight into general interest in healthcare spiritual history/assessment (Table 2).

Articles originated from almost all world regions with approximately half (*N* = 305;53.4%) from United States, followed by United Kingdom (*N* = 62;10.9%), Western Europe (*N* = 42;7.4%), South & Central America (*N* = 35;6.1%) and Oceania (*N* = 30;5.3%). Twenty-one languages were represented, with English most common (*N* = 477; 83.5%), followed by Portuguese (*N* = 22), Spanish (*N* = 18), and German (*N* = 14).

The most common disciplines were medicine (*N* = 313;54.8%), nursing (*N* = 88;15.4%), psychology (*N* = 43;7.5%), chaplaincy (*N* = 35;6.1%), and social work (*N* = 28;4.9%). Common medicine subspecialties were palliative care (*N* = 82;26.2%), psychiatry (*N* = 45;14.4%), family medicine (*N* = 41;13.1%) and oncology (*N* = 34;10.9%).

### Expert opinion evaluation of HOPE and other spiritual history/assessment tools

266 sources included some assessment of HOPE, reflecting authors’ expert opinion, literature review, experience, and/or formal studies (Supplementary Table-1; Table-2). Qualitative analysis of authors’ extracted evaluative comments revealed strengths/weaknesses of HOPE and other spiritual history/assessment tools (Table 3).

Perceived strengths of spiritual history/assessment tools in general (most commonly mentioned– HOPE [36], FICA [35], SPIRIT [37]) were ease of use and ability to enhance patient-centered care. Their collective weaknesses were perceived lack of validation. Some authors, when describing these SH/SA tools a whole, expressed concern regarding lack of cultural diversity.

Several sources described relative strengths of the HOPE model. A commonly discussed strength is HOPE’s acceptability among diverse, multicultural and/or secular populations. Sources felt that this was due to its opening questions, which focus on general spiritual resources rather than starting by asking about religion, spirituality or faith. Another commonly discussed strength was its flexibility due to its 4 domains with suggested, but not required, questions. This combination of structure plus flexibility allowed for its adaptation and use by both novice and expert clinicians. HOPE’s most frequently discussed weaknesses, like other similar tools, was perceived lack of validation. Some authors also expressed concern over its length and its need for a conversation; whereas others pointed out that specific questions within each domain are only suggestions and considered the conversational aspect a strength.

See Table 3 and Table 1 for details of comments linked to specific HOPE domain and questions. Together, these 266 articles provide evidence for face validity.

#### Specific uses and/or evaluation of the HOPE model

Sixty-three articles described specific experiences using the HOPE model and/or formal evaluation/studies of HOPE. These articles underwent in-depth data extraction and analysis (Tables 4, 5, 6), [33–34, 64–124] revealing insights into diversity of uses, acceptability, feasibility and validity, including most commonly utilized HOPE questions/domains (Table 1; see*).

### Specific uses/adaptions of HOPE; no formal evaluation

32 articles described uses or adaptions of HOPE but did not provide evaluative data regarding HOPE (Table 4) [64–95]. These originated from 11 countries; 2 published in Portuguese and 1 in Czech. Sources utilized HOPE in clinical (*n* = 9), [64–72] educational (*n* = 9),[73–81] research (*n* = 12), [82–93] and other (*n* = 2) [94–95] settings.

Of the 9 articles [64–72] describing clinical uses, two [64, 66] report routine use of HOPE on *all* patients. Wynn [66] reports that all patients admitted to a UK hospice facility undergo the HOPE spiritual assessment, and Brady [64] describes using a questionnaire based on HOPE on all patients in their chronic pain clinic. Four are case studies providing literature review and detailed descriptions of how authors used HOPE with a patient [69–72]. Gibeau [65] describes creating a wellness model, specific to addiction, based on HOPE and two other models. Gomi [67] and Hydit [68] used questions from HOPE and other spiritual history/assessment models to create and test new models for psychiatric patients [67] and cancer patients [68]. 

Nine articles describe educational uses of HOPE, without evaluation data [73–81]. Two selected HOPE as their exclusive spiritual history/assessment teaching tool [76, 77]. Five used HOPE plus other tools [73–75, 78, 81]. Two used HOPE to create their own training tool for MICU nurses [79] and rehabilitation professionals [80]. Four were required student sessions [73, 74, 77, 81] - Feldstein reports successful implementation with 500 medical students [74], Sandor with 416 medical and nursing students [73] and Hunt and Elliot with social work students over many years [77, 81]. 

Twelve articles describe adaptations of HOPE for their research studies [82–93]. All used some, but not all domains of HOPE. Five were qualitative studies [83, 84, 87, 91, 93], 5 were quantitative [82, 85, 86, 88, 92] and 2 were mixed methods [89, 90]. Seven used other spiritual history/assessment tools in addition to HOPE, to develop their own research instrument [83–85, 89, 90, 92, 93]. 

Two articles described utilizing HOPE for other settings. Kuhl [94] slightly modified the full HOPE model for use by AirForce commanders to assess airmen wellbeing. Kellaher [95] describes using HOPE with the Appreciative Inquiry Model to improve organizational culture.

All these articles demonstrate face validity of HOPE. Some illustrate feasibility in specific settings (Table 4).

**Table 4:** Articles describing specific uses or adaptions of the HOPE model WITHOUT explicit evaluation of HOPE (*N* = 32)

Author Year Reference Type	Type of Use Discipline Country Article Language Translations	Specific Setting Population Number	Use of HOPE	Description of Use	Evidence for Acceptability, Feasibility or Validity?
**CLINICAL**					
**2007** **Brady S**[64] Book Chapter	● **Clinical** ● Medicine ● United States ● English ● N/A	● Pain medicine clinic ● Patients with chronic pain patients ● n = **“all”**	Adapted as a clinical tool, routinely used for all patients	The author uses a questionnaire, based on HOPE, for all patients in their chronic pain clinic. *“I take a spiritual history on all patients…. Although there are many different types of spiritual health inventories*,* I have created my own inventory around the HOPE format… HOPE is an anacronym for a 4 category approach to asking patients about spirituality: H - ask about sources of hope……”*	Acceptability: yes Feasibility: yes Face Validity: yes
**2009** **Gibeau C**[65] Thesis	● **Clinical** ● Social Work/ Addiction ● United States ● English	● Social Work ● Alcohol Use/Addiction ● n = N/A	Used to develop a model specific to addiction	The HOPE model was used alongside 2 other spiritual wellness models (Purdy 2005 & Westgate 1996) to create a modified personal faith-based wellness model that can be used in combination with traditional medicine as a treatment for individuals with alcohol abuse and addiction.	Acceptability: yes Feasibility: N/A Face Validity: yes
**2013** **Wynne L**[66] Journal Article	● **Clinical** ● Hospice/ Palliative Care ● England ● English ● N/A	● A hospice center ● Admitted patients ● N = **“all”**	Used for routine clinical intake on all patients	The HOPE spiritual assessment is *routinely* conducted on admission to this hospice facility, and a spiritual care plan is created for each patient. The spiritual care plan is updated regularly to make sure patients’ spiritual needs are being met routinely and assessed continually.	Acceptability: yes Feasibility: yes Face Validity: yes
**2013** **Gomi S**[67] Journal Article	● **Clinical** ● Mental Health ● United States ● English ● N/A	● Community mental health centers ● Mental health providers and consumers ● *N* = 48	Used to create a new SH/SA tool for patients with psychiatric disabilities	Authors used HOPE, FICA, SPIRIT, MIMBRA, and other sources to create a new set of spiritual assessment questions for use as a clinical communication tool with patients dealing with severe mental illness. The new questions were then tested using a focus group at 3 urban community mental health centers.	Acceptability: yes Feasibility: N/A Face Validity: yes
**2017** **Hvdit EA**[68] Journal Article	● **Clinical** ● General Practice (GPs) ● Denmark and Norway ● English ● N/A	● GPs in Denmark; Patients with cancer ● 31 GPs and 24 cancer patients ● *N* = 55	Used to create a new tool for facilitating existential communication with cancer patients, for the Dutch context.	Based on literature review and criteria, authors selected HOPE, FICA and SPIRIT as examples of tools to use as a foundation to develop their own tool for “existential communication” between GPs and patients with cancer (the EMAP). A draft of the new tool was presented to GPs and cancer patients for feedback and then underwent 2 rounds of an expert Delphi panel process to finalize the tool.	Acceptability: yes Feasibility: N/A Face Validity: yes
**2003** **Larson K**[69] Journal Article	● **Clinical Case Study** ● Nursing ● United States ● English ● N/A	● Post-operative care ● Patient with amputation ● *N* = 1	Case Study (detailed account of clinical use) re: post-op recovery	Author briefly reviews spiritual assessment including the SPIRIT and HOPE models. She provides one case study describing how she, as a nurse, used the HOPE model with an elderly patient following below-the-knee amputation for a non-healing ulcer. Demonstrates how spiritual coping strategies can help with grieving over loss of a limb and subsequent adjustment and recovery.	Acceptability: yes Feasibility: yes Face Validity: yes
**2011** **Chrash M**[70] Journal Article	● **Clinical Case Study** ● Nursing ● United States ● English ● N/A	● Follow-up hospitalization ● Patient with chronic illness (COPD) ● *N* = 1	Case Study (detailed account of clinical use) re: advanced care planning	An Advanced Practice Nurse performed a follow-up assessment on a patient after hospitalization for an acute COPD exacerbation. The HOPE tool was applied to assist in a holistic assessment and developing an advance care plan; 1–2 questions from each of the 4 domains was asked, as part of a medical interview. HOPE was found to help effectively and efficiently determine patients’ preferences and resulted in advance care planning concordant with the patient’s beliefs and values (visit took 30–35 min).	Acceptability: yes Feasibility: yes Face Validity: yes
**2015** **Hemphill B**[71] Journal Article	● **Clinical Case Study** ● Occupational Therapy ● United States ● English	● Occupational therapy clinic ● Addiction ● *N* = 1 (case study & review)	Case Study (detailed account of clinical use) re: recovery/ addiction	Reviewed principals of spiritual assessment and several spiritual assessment tools (e.g. Koenig’s approach, FICA, SPIRIT, HOPE). Chooses to use HOPE in the case study. Demonstrated how the discussion elucidated how the patient’s Buddhist beliefs helped him cope with his addiction and family conflicts.	Acceptability: yes Feasibility: yes Face Validity: yes
**2016** **Finocchiaro D**[72] Journal Article	● **Clinical Case Study** ● Nursing ● United States ● English ● N/A	● Outpatient care/home visits ● Patient with terminal cancer ● *N* = 1	Case Study (detailed account of clinical use) re: end-of-life care	Description of how a nurse used the HOPE model during outpatient visits to assess and address the spiritual needs of a 40-year-old woman with end-stage colorectal cancer, who had young children. Questions from **all 4 domains** of HOPE were used, which allowed the nurse to tailor care to the patient’s needs.	Acceptability: yes Feasibility: yes Face Validity: yes
**EDUCATION**					
**2006** **Sandor MK**[73] Journal Article	● **Education** ● Medicine & Nursing ● United States ● English ● N/A	● Medical school & nursing school ● Junior nursing students (*n* = 122) and first year medical students (*n* = 194) ● *N* = 416	Used as an educational tool	Used in a 2 week “Spirituality and Clinical Care” course curriculum for med students and nursing students. Teaching method was didactics and small group case discussions. Students were given information on 4 spiritual assessment tools (HOPE, FICA, SPIRIT and Kinney), but did not receive specific skills training. Evaluation was about the overall curriculum’s effect on students’ attitudes and development. No specific feedback or evaluation of HOPE or other tools.	Acceptability: yes Feasibility: yes Face Validity: yes
**2008** **Feldstein BD**[74] Journal Article	● **Education** ● Family Medicine ● United States ● English ● N/A	● Stanford Medical School ● Fam Med Clerkship ● N = approx. 500 students.	Used as educational tool	This article describes the experience of successfully integrating a curriculum on spirituality and multicultural literacy, with a focus on end-of-life care, into the required family medicine clerkship at Stanford Medical School. Students learn how to explore patients’ sources of hope, strength, and meaning, including spiritual and religious beliefs using the HOPE or FICA models for spiritual assessment. No curriculum evaluation is provided.	Acceptability: yes Feasibility: yes Face Validity: yes
**2011** **Dugan BDA**[75] Journal Article	● **Education** ● Pharmacy ● United States ● English ● N/A	● Private, Christian University ● Students at the School of Pharmacy ● *N* = 24	Used as an educational tool	Describes an elective spiritual care course for pharmacy students. Students were provided SPIRIT, FICA and HOPE as examples of spiritual assessment tools. They were instructed to choose one assessment tool to complete two practice spiritual history interviews with other students in the class. No specific evaluation of students’ spiritual assessment knowledge or skills provided.	Acceptability- yes Feasibility-yes Face Validity- yes
**2012** **Prescott G**[76] Journal Article	● **Education** ● Pharmacy ● United States ● English ● N/A	● University of Buffalo School of Pharmacy ● Third and fourth year students ● N = “all”	Used as an educational tool	Describes a 6-week clinical elective on ethics and spirituality for clinical pharmacology students. Students are given the HOPE article prior to their clinical elective. In addition to other requirements (e.g. reflective essays, cases), students are required to conduct a minimum of 4 spiritual assessments, using the HOPE format, with patients at clinical sites that have chaplains as part of the team (2 at the faith-based free clinic and 2 at the HIV/AIDS clinic). These are graded by the rotation faculty. This article does not provide course or student evaluation data.	Acceptability- yes Feasibility- yes Face validity- yes
**2017** **Elliot N**[77] Journal Article	● **Education** ● Social Work ● United Kingdom ● English ● N/A	● Kingston University ● Students in the School of Social Work ● N= “all”	Used as an educational tool	Uses the HOPE model for a 2–3 h teaching session for social work students. Does not name HOPE or show the whole model in the article. However, it quotes one question from HOPE in the text– “*for some people*,* their religious or spiritual beliefs act as a source of comfort and strength in dealing with life’s ups and downs. Is this true for you?”* Authors explain that this question provides a “safe, normalizing” way of opening the door to discussion. They use this question with students in a pair-share activity and advise students that if they receive a positive response with patients that they can follow up with other questions from the HOPE model.	Acceptability- yes Feasibility: yes Face Validity: yes
**2017** **Milstein G**[78] Journal Article	● **Education** ● Psychiatry ● United States ● English ● N/A	● Mental health wellness program ● Conference participants ● *N* = 59	Used as an educational tool	Describes a mental health wellness program done in collaboration between consumers, clergy, & clinicians. Mentions that as part of this they train clinicians in how to include spirituality and religion in their assessment and treatment planning. They present 3 tools in their clinician training - HOPE, FICA and Pargament, 2007. They evaluate the whole program but no evaluation of spiritual assessment training specifically.	Acceptability- yes Feasibility- yes Face Validity- yes
**2018** **Patton LA**[79] Thesis	● **Education** ● Nursing ● United States ● English ● N/A	● Medical Intensive Care Unit ● MICU Nurses ● *N* = 12	Used to develop a new educational tool	Used HOPE as background information to develop their own teaching tool for MICU nurses. Tested their own teaching instrument in this study.	Acceptability: N/A Feasibility: N/A Face Validity: yes
**2020** **Jones KF**[80] Journal Article	● **Education** ● Rehabilitation ● Australia ● English ● N/A	● Rehabilitation hospital ● Rehab professionals (multiple disciplines) ● *N* = 16	Used to develop a new educational tool	Created their own “spiritual care training tool” for rehab setting, after reviewing HOPE and FICA, with a greater emphasis on spiritual strength and how rehab professionals can help. Did a qualitative study assessing their whole spiritual care training program. Does not provide their tool in the article.	Acceptability: N/A Feasibility: N/A Face validity: yes
**2022** **Hunt J**[81] Journal Article	● **Education** ● Social Work ● United States ● English ● N/A	● Belmont University ● Social work students ● N= “all”	Used as an educational tool	Used in teaching spiritual assessment to social work students; students examine 4 tools - HOPE, Hodge’s SA “toolbox” for SW, JCAHO questions and Nelson-Baker’s SA model for geriatrics - as well as any tools they have encountered in clinical settings. They then develop their own interview guide in groups and conduct a spiritual self-assessment. No curriculum evaluation reported, however authors offer “lessons learned” over 10 years teaching this curriculum.	Acceptability: yes Feasibility: yes Face Validity: yes
**RESEARCH**					
**2006** **Fick JL**[82] Thesis	● **Research** ● Genetic Counseling ● United States ● English ● N/A	● Genetics clinic ● 43 prenatal patients & 103 caretakers of pediatric patients ● *n* = 146	Adapted and used in a research study (quantitative)	HOPE model was used to develop 8 questions out of a 21-question questionnaire to assess the views of patients and caretakers on the relevance and importance of discussing spirituality during genetic counseling appointments.	Acceptability: yes Feasibility: N/A Face Validity: yes
**2010** **Seth SG**[83] Journal Article	● **Research** ● Genetic Counseling ● United States ● English ● N/A	● A general hospital ● Pregnant Latina patients referred for genetic counseling ● *n* = 11	Adapted and used in a research study (qualitative)	Used HOPE and DUREL to develop their own interview guide for a qualitative study of Latina women’s decisions regarding amniocentesis	Acceptability: yes Feasibility: N/A Face Validity: yes
**2010 Thangathurai**[84] Thesis	● **Research** ● Internal Medicine/ Oncology ● United States ● English ● N/A	● Oncology hospital ● Physicians & nurses ● *N* = 11	Adapted and used in a research study (qualitative)	The qualitative portion of this mixed method study used interview questions derived from FICA and HOPE. Interviews were conducted with physicians and nurses, exploring their beliefs on how spirituality impacts the care of terminally ill patients.	Acceptability: yes Feasibility: N/A Face Validity: yes
**2011** **Iranmanesh S**[85] Journal Article	● **Research** ● Nursing ● Iran ● English ● Farsi	● Kerman Medical University ● Third and fourth year nursing students ● *N* = 200	Used to develop a research questionnaire (quantitative)	Used HOPE and 3 other tools (Narayanasamy, McSherry and Burkhardt) and key informant interviews to create and test their own quantitative questionnaire assessing the beliefs and perceptions of nurses in the Iranian context.	Acceptability: yes Feasibility: N/A Face Validity: yes
**2014** **Adanikin AI**[86] Journal Article	● **Research** ● Obstetrics ● Nigeria ● English ● 2 local Nigerian languages	● Obstetric clinics at two teaching hospitals ● Antenatal patients ● *n* = 397	Adapted and used in a research study (quantitative)	Aspects of HOPE were adapted and used as a self-administered questionnaire for a study to assess the spiritual care needs of patients during pregnancy and childbirth in Nigeria, where there is a high maternal and perinatal mortality rate & patients seek prayer houses over hospitals for their delivery location. The questionnaire was pretested at 2 teaching hospitals. *“Content validity was ensured by translating the questionnaire to major local languages and back translated into English.”*	Acceptability: yes Feasibility: N/A Face Validity: yes Language Adaptability: yes
**2016** **Motl J**[87] Thesis	● **Research** ● Psychiatry ● Czech Republic ● Czech ● Czech	● Psychiatric Hospital in Havlíÿkÿv Brod ● Patients diagnosed with psychotic illnesses ● *n* = 9	Used to develop an interview research guide (qualitative)	Used one question from HOPE in their semi-structured interview guide for their qualitative study of the role of spirituality in people who experience psychosis. (Translated to Czech) - *“What aspect of your spirituality or your spiritual practice do you find most helpful?”*	Acceptability: yes Feasibility: N/A Face Validity: Yes Language adaptability: yes
**2017** **Briggs M**[88] Journal Article	● **Research** ● Family Medicine ● United States ● English	● interdenominational churches ● African American seniors ● *n* = 67	Adapted and used in a research study (quantitative)	Questions from HOPE were adapted for use in a quantitative survey assessing the effects of a church-based health education program on fall risk in the elderly. HOPE was the only spiritual assessment tool used. However, assessment was multidimensional (included medication use, social support, hospitalizations, falls, independence in daily activities, depression, etc.). Details of HOPE specific data were not provided.	Acceptability: yes Feasibility: N/A Face Validity- yes
**2018** **McNair T**[89] Thesis	● **Research** ● Nursing ● United States ● English ● N/A	● Nursing school ● Faculty Nurses ● *n* = 19	Used to develop their own survey for research study (mixed methods)	HOPE and FICA were used as a foundation for developing their own survey questions for this study. Goal of the study was to assess nurses’ perspectives on spiritual care and explore if they believe there is a connection between spiritual care and healing.	Acceptability: yes Feasibility: N/A Face Validity: yes
**2018** **Oliviera JAC**[90] Thesis	● **Research** ● Family Medicine ● Brazil ● Portuguese ● Portuguese	● National survey ● Family Physicians in Brazil ● *n* = 547	Adapted for use along with other tools as part of a larger study (mixed methods)	This study aimed to widely assess Brazilian family physicians’ attitudes towards spiritual care, barriers to providing spiritual care, their spiritual well-being and the frequency with which they discuss spiritual topics during clinical care. Several research questionnaires were used (e.g. FACIT-SpNI & Durel-P). Elements from HOPE, FICIA, and SPIRIT were used to evaluate the frequency and themes of spiritual topics assessed by physicians in practice.	Acceptability: yes Feasibility: N/A Face Validity: yes
**2019** **Brown JL**[91] Journal Article	● **Research** ● Gynecology ● New Zealand ● English ● N/A	● Physiotherapy clinics ● Patients with pelvic surgery complications ● *n* = 7	One question used in a research study (qualitative)	A question adapted from HOPE was used as part of a mixed method study to determine impact of complications from pelvic mesh surgery on their lives. The question used was: *“For some people their religious or spiritual beliefs act as a source of comfort and strength in dealing with life’s ups and downs; what has been your experience?”*	Acceptability: yes Feasibility: N/A Face Validity: yes
**2022** **Barbosa ACP**[92] Thesis	● **Research** ● Family Medicine ● Portugal ● Portuguese ● N/A	● Practices across mainland Portugal ● Family Physicians ● *N* = 165	Adapted to create their research questionnaire (quantitative)	Used FICA and HOPE to create their own quantitative instrument for their study aimed at assessing physicians’ approach to spirituality/ religion in the clinical setting. As part of the study, participants rate how often they use certain questions with their patients (including several from the HOPE tool). Findings: Most physicians do not discuss spirituality/religion with patients, even though they think it is appropriate in the medical context. Most common questions asked are: a) what specific practices or restrictions should be taken into account during medical care (35.8%); (b) is the person religious, spiritual or a person or faith (27.3%); (c) whether the person has any personal spiritual practices they use (e.g. prayer, meditation) 26.7%.	Acceptability: yes Feasibility: N/A Face Validity: yes
**2023** **Mastrangelo S**[93] Journal Article	● **Research** ● Internal Medicine ● Switzerland ● English ● N/A	● A dialysis center ● Patients on hemodialysis ● *N* = 20	Used to develop a new research instrument (qualitative)	Used HOPE and FICA as inspiration to create their own qualitative research instrument. Study goal was to explore the spiritual expectations of dialysis patients in a Swiss dialysis center. Details of use not provided.	Acceptability: yes Feasibility- N/A Face Validity- yes
**OTHER**					
**2005** **Kuhl**[94] Research Report Air Force	● **Other** ● Airforce ● United States ● English ● N/A	● Airforce Commander development	Slightly adapted for use for assessing well-being of Airmen	The author slightly modified the HOPE model as a practical guide for Airforce Commanders, charged with ensuring the overall well-being of Airmen, including their spiritual wellbeing. Airmen face significant stress in their roles. The author argues that while physical, emotional and social wellbeing are generally well understood, spiritual wellbeing is less understood. He argues that the HOPE model provides a guide to open discussions, that is respective of a wide range of views regarding spirituality. No evaluation data provided.	Acceptability: yes Feasibility: N/A Face Validity: yes
**2021** **Kellaher K**[95] Thesis	● **Other** ● Healthcare Administration ● United States ● English ● N/A	● Healthcare Leadership Development (MBA project)	Used to create a model for improving organizational culture	The author combined the Appreciative Inquiry Model and the HOPE Model as a method that leaders can use to create and support best practices in the field of healthcare administration. They note that the application of HOPE could aid in creating an organizational culture of empathy and respect for diversity. They aim for this new model to help cultivate “servant leaders”. They did not test their model.	Acceptability: yes Feasibility: N/A Face Validity: yes

#### Studies/Evaluation of the HOPE model or interventions using HOPE

Studies offering formal evaluation of HOPE (*N* = 31) [33–34, 96−124] included those examining interventions using HOPE (*N* = 17; Table 5) [96–112] and those evaluating the HOPE model itself, with or without other similar tools (*N* = 14; Table 6) [33–34, 113–124] These 31 studies represent 10 countries, were published in 4 languages and translated into 6 languages.

##### Studies evaluating interventions utilizing HOPE

The 17 studies (Table 5) [96–112] evaluating interventions using the HOPE model included clinical (*N* = 3), [96–98] educational (*N* = 7)[99–105] and research settings (*N* = 7) [106–112].

Clinically, Blum’s study [96] revealed that using 1–3 questions from each of the 4 domains of HOPE, as a routine screening tool on all patients admitted to their inpatient palliative care unit in France, resulted in an increase in chaplain referrals from 8.5 to 28%. In India, Paswan [97] successfully used one question from each HOPE domain as a clinical screening tool for their diverse population, to identify patients for whom spirituality was important for inclusion in their study on integrating patient-selected spiritual practices into psychiatric treatment plans. Stratton [98] successfully used HOPE as a clinical standard for inclusion in their study of the effects of chaplain visits. A meaningful chaplain visit was determined to have occurred if the chaplain note indicated that at least one domain of HOPE had been discussed.

Seven studies of educational interventions using HOPE encompassed medical student (*N* = 1),[99] resident (*N* = 3) [100, 102, 104] and nursing (*N* = 3) [101, 103, 105] education. In all 7, educators selected HOPE as the only spiritual history/assessment training tool. Most studies used multiple evaluation methods. According to Kirkpatrick’s 4 levels of educational evaluation [113], four studies reached level-2 (knowledge and skills acquisition) [99, 100, 103, 105]. One study was longitudinal and included level-3 evaluation (changes in clinical approach/practice), [102] and two reached level-4 evaluation (measurable organizational-level improvements) [101, 104]. Two studies [100, 105], specifically included faculty training. Bush’s study [105] also provides students’ feedback regarding HOPE, providing evidence of stakeholder acceptability. Together, these 7 studies demonstrate acceptability, feasibility and validity of the HOPE model as a teaching tool.

Seven studies [106–112] used HOPE (all 4 domains) for their interview guides. In all studies HOPE met researchers’ needs, demonstrating feasibility and validity as a qualitative research instrument.

##### Studies evaluating the HOPE model itself, with or without other tools

Fourteen studies (Table 6)[33–34,113−124] directly evaluated the HOPE model and its questions. Of these, 10 compared HOPE with other spiritual history/assessment tools [33–34, 117−124] and 4 examined the HOPE model alone [114–117].

Several of the 10 studies comparing spiritual history/assessment tools used similar methods. In five [33, 34, 118–120], researchers used literature review to develop criteria for rating content validity of several tools. Five studies [121–125] used consensus approaches (e.g. Delphi technique, multi-step group process) to elicit expert/stakeholder/patient opinion regarding content validity and feasibility. Most studies found several spiritual history/assessment tools with similar ratings, but studies varied in rating criteria and which tools they studied. HOPE achieved strong ratings in all studies. Other common highly rated tools were SPIRIT [37], FICA [35], FAITH [38] (Table 6). Two articles concluded that HOPE was the most appropriate tool for their setting [34, 121].

In four studies, authors chose to only evaluate HOPE [114–117]. In each, authors selected the HOPE model as appropriate for their setting, based on literature review, and tested their hypothesis. Reis’s [114] survey of USA genetic councilors (*N* = 127) found that H & E questions were rated most relevant (93%, 86%) and that 69% “definitely” or “might” use HOPE with their clients. Koster’s [115] small study (*N* = 9) of Dutch general practice physicians, aiming to determine if HOPE could facilitate patient communication regarding “meaning” revealed mixed results. Fopka-Kowalczk [116] selected HOPE for use in Poland and reports validation of HOPE in Polish. Finally, Whitehead [117] selected HOPE for possible use by general practitioners in England. This feasibility study revealed that 65% of general practitioners would be comfortable using HOPE with patients and 77% would be comfortable being asked these questions if they were a patient.

## Discussion

### Main study findings and gaps

#### General findings

This scoping review aimed to broadly characterize interest and uses of the HOPE model for spiritual assessment. To our knowledge, this is the first systematically-constructed review of any spiritual history/assessment communication tool created for clinicians who are not spiritual care specialists. Our review revealed: (1) broad interest in HOPE, spanning numerous continents, languages and disciplines; (2) studies providing insight into effective uses of HOPE in clinical, educational and research settings; and (3) studies testing the acceptability, feasibility and validity of this communication model.

#### Global interest in HOPE and other similar tools

This review found 571 articles that specifically mention/cite the HOPE model. 68% also discussed other tools, indicating that these articles represent interest in spiritual history/assessment in general, rather than HOPE in particular. While over half originated from the USA, most world regions are represented (Table 2). Similarly, though English was most common, 21 languages are represented. Common interested disciplines are medicine, nursing, psychology, chaplaincy and social work. This widespread interest in the “how” of incorporating spiritual history/assessment into clinical practice likely stems from increasing recognition of the value of spiritual care in holistic patient-centered care, organizational-level requirements, and paucity of training opportunities [12–21, 42−43, 126–127]

#### Persistent lack of knowledge of existing spiritual history/assessment tools

Despite broad interest in spiritual history/assessment and the persistent mismatch between patients’ needs and provision of spiritual care, [42–44, 126−127] this review reveals a lack of knowledge of existing tools. For example, studies by Brown [123] and Jones [124] show that while participants believe spiritual assessment is important, few had heard of HOPE or other tools (e.g. FICA [35], SPIRIT [37]).

#### Strengths and weaknesses of the HOPE model

Authors who selected HOPE for use in their setting and/or evaluated HOPE (Tables 4, 5 and 6) and those who offer expert opinion evaluations (Table 3) point to the following strengths (see Table 1): (1) H Domain– allows for conversation with people from diverse backgrounds (secular, religious, multicultural); (2) HOPE’s normalizing and transition statements - facilitate inclusive, non-threatening conversations; (3) Flexibility − 4 domains of inquiry with suggested (but not required) questions provide guidance for novice clinicians and flexibility for expert clinicians; (4) Strength-Based Approach– allows patients to identify/utilize their own spiritual resources; (5) Asks if anything has changed– facilitates identification of potential spiritual suffering or distress; (6) The E Domain– can be used for simple history taking by novices or shared decision making by experienced clinicians.

**Table 5:** Evaluations of interventions using the HOPE model for spiritual assessment (*N* = 17)

Year Author Reference Type	● Type of Use ● Discipline ● Country ● Article Language ● Translations	Specific Setting Population Number	Intervention using HOPE (or How HOPE was used)	Study Method	Main Findings/ Outcomes	Evidence for Acceptability, Feasibility or Validity?
**CLINICAL USES AND EVALUATION OF THE HOPE MODEL**						
**2014** **Blum D**[96] Thesis	● **Clinical** ● Palliative Care ● France ● French ● French	● Inpatient Palliative Unit in France ● All admitted patients ● *N* = 495 125 (study group) 370 (comparison)	● HOPE used as an interview guide for spiritual assessment on *all* patients admitted to the inpatient palliative care unit. ● All 4 domains of the HOPE model were used with 1–3 slightly modified questions from each domain. ● Translated to French.	*Study question*: What is the impact of routine spiritual assessment (HOPE) on addressing patients’ spiritual needs? *Methods*: • Quantitative study • Measured the number of referrals to chaplain before and after implementing routine admission HOPE spiritual assessment	Routinely conducting a spiritual assessment (using the HOPE model) on admission increased the use of chaplains by patients/families. *Specific findings*: ● During the study period, 28% of patients received a chaplain visit (*n* = 35) compared 8.2% and 8.5% in the two years used as control (2011 & 2012). *p* < 0.0001.	Acceptability: yes Feasibility: yes Validity: Face - yes Content - yes Teaching tool - N/A Clinical tool - yes Research tool - N/A Language– yes (French)
**2019** **Paswan RK**[97] Journal Article	● **Clinical/** **Research** ● Medicine -Psychiatry ● India ● English ● Unknown	● Outpatient Psychiatry Setting in India ● Patients (diverse religions– Hindu, Muslim, Sikh, Jain) ● *N* = 510 260 (study group) 250 (control)	● The HOPE model was used as a clinical screening tool for study inclusion. ● All 4 domains of the HOPE model were used (one question from each domain) ● The HOPE model was also used as a qualitative questionnaire at the 1 month and 6-month follow-up.	*Study question*: Does incorporating spiritual practices into the psychiatric treatment plan improve outcomes? *Methods*: ● Patients with “high spirituality” were included (HOPE interview) ● Study group: given standard psychiatric treatment and allowed to incorporate spiritual practices of their choosing into treatment. ● Control group: given standard treatment and not allowed to incorporate spiritual practices. ● Outcome measures: WHOQOL-BREF and HOPE at 0, 1, 6 months	The HOPE model was successfully used as a clinical screening tool for study inclusion (i.e. to identify people for whom spirituality is important) and also as a qualitative assessment tool throughout the study. *Findings of the overall study*: ● Both groups had improvement in WHOQOL-BREF scores as the study progressed. ● The study group had statistically significant better scores on WHOQOL-BREF at months 1 & 6 in comparison to control group.	Acceptability: yes Feasibility: yes Validity: Face - yes Content - yes Teaching tool - N/A Clinical tool - yes Research tool– yes (qualitative) Language-unknown
**2019** **Stratton R**[98] Thesis– PhD	● **Clinical/ Research** ● Chaplaincy ● United States ● English ● N/A	● Hospital chaplain service ● Patients admitted to acute care hospital, who had received a chaplain visit ● *N* = 1654 ● *N* = 160 80 (religious) 80 (non-religious)	● The HOPE was used as a clinical standard for inclusion in the study. ● All 4 domains of the HOPE model were used ● Patients were included if they had received a chaplain intervention during the hospital stay, as determined by a chaplain visit documenting discussion of at least one domain of HOPE and duration of visit greater than 5 min.	*Study goal*: assess patient satisfaction in religious and nonreligious patients receiving chaplain interventions. *Methods*: ● Random selection of patients who filled out the HCAHPS patient satisfaction survey underwent chart review for study inclusion. ● Patients who had received a chaplain intervention (HOPE criteria) were included. ● Included patients were divided into religious and nonreligious groups ● Statistical analysis of HCAHPS scores of both groups.	The HOPE model was successfully utilized as a clinical standard to determine whether a meaningful chaplain intervention had occurred in a diverse patient population *Findings of the overall study*: ● No statistically significant differences in satisfaction between religious and non-religious patients receiving chaplain services. ● Recommendations: Spiritual needs of non-religious patients warrant further study.	Acceptability: yes Feasibility: yes Validity: Face -yes Content– yes Teaching tool– N/A Clinical tool– yes Research tool– N/A Language– N/A
**EDUCATIONAL USES AND EVALUATION OF THE HOPE MODEL**						
**2004** **King DE**[99] Journal Article	● **Education** ● Medicine– medical students ● United States English ● N/A	● Medical School ● Medical Students ● *N* = 146	● HOPE selected as the spiritual history training tool for medical students. ● Integrated longitudinal spiritual history training into the first-year medical school Doctoring Course. *Educational Intervention*: ● Students were given readings regarding spiritual care. ● HOPE (all 4 domains) was used to teach & practice spiritual history skills in two standardized patient sessions in fall and spring semesters	*Study goals*: To evaluate the effectiveness of spiritual history training using HOPE. *Methods*: 3 components: ● The videotaped end-of-year OSCE examination using standardized patient (SP), which included relevant spiritual history ● A survey regarding students’ attitudes towards incorporating spiritual care into medical care pre- and post- intervention ● Including a didactic question regarding the HOPE model in the end-of-year written exam.	HOPE was validated as an effective training tool for medical students. ● In videotaped SP interviews, 30% of students asked about sources of hope, 50% asked about organized religion, 23% asked about religious/spiritual believes in medicine, and 38% asked about personal spiritual practices. ● On the attitudes questionnaire there was a modest increase in the desire to accommodate patients’ beliefs in medical treatment plans ● 94% students answered question on exam about HOPE correctly	Acceptability: yes Feasibility: yes Validity: Face - yes Content - yes Teaching tool– yes Clinical tool– N/A Research tool - N/A Language - N/A Kirkpatrick Training Evaluation Level: 1 & 2
**2010** **Attar MA**[100] Journal Article	● **Education** ● Medicine -Pediatrics & Med-Peds ● United States English ● N/A	● Residency Program ● Pediatric and Med-Peds Residents ● *N* = 34 ● (23 study group; 11 comparison group)	HOPE selected as the training tool for a residency curriculum on communicating bad news (CBN), including eliciting the spiritual needs *Educational Intervention*: ● A longitudinal curriculum ● Residents given references plus a pocket card with CBN model plus the HOPE model ● Residents received a 1-hour didactic each year (3) with peer role-play, plus direct observation in the NICU. ● All 4 domains of the HOPE model were used to teach ● Faculty input solicited and faculty training provided.	*Study goals*: To assess the effectiveness of this longitudinal curriculum *Methods*: ● Residents’ self-assessment of confidence with their skills ● Residents’ performance on a standardized patient exercise. ● The intervention groups’ scores were compared pre- and post- intervention (as 1st year and 3rd year residents). ● 3rd year residents’ scores (intervention group) were compared with a comparison group who had not received the curriculum.	HOPE was validated as an effective training tool for residents. This curriculum, utilizing HOPE, resulted in improved observed clinical skills and self-assessment regarding providing spiritual care to seriously ill children & families. ● Compared to comparisons groups, the study group reported more frequently exploring parents’ needs for spiritual support ● Compared to comparison groups, the study group scored higher on standardized patient exercises. ● This curriculum was feasible to implement and continued to be implemented past the 3-year study.	Acceptability: yes Feasibility: yes Validity: Face - yes Content - yes Teaching tool– yes Clinical tool– yes Research tool - N/A Language - N/A Kirkpatrick Training Evaluation Level: 1 & 2
**2011** **Lind B**[101] Journal Article	● **Education/ Clinical** ● Nursing ● United States ● English ● N/A	● Cardiovascular Care Unit ● CCU Nursing Staff ● *N* = 37	HOPE was selected as the spiritual history/assessment method to train CCU nurses. *Educational Intervention*: ● Nurses underwent a 2-hour spiritual care training using the HOPE model (all 4 domains) ● HOPE questions were modified and printed on a card for nurses to use as a framework to assess patients’ spiritual needs.	Study goals: To assess the effects of the spiritual care training session with HOPE & the HOPE pocket card: ● comparison of responses on patient satisfaction survey re: “how well the hospital staff addressed your spiritual and emotional needs?” in the 4 quarters before and 2 quarters after the teaching intervention ● number of consultations requested by nursing staff to hospital chaplains and frequency of spiritual care plan use pre/post intervention ● anecdotal nurse feedback.	HOPE was validated as an effective teaching tool AND clinical tool. ● Patient satisfaction with the CCU was greater post intervention (74%, 71%) compared to pre- (62–69%) and compared to hospital-wide score (65%). ● The number of pastoral care consultations increased from average of 16/month to 27/month ● use of the spiritual care plan increased from 0 to 1–4 uses/month post-training ● anecdotally, nurses felt more comfortable assessing and intervening with spiritual care.	Acceptability: yes Feasibility: yes Validity: Face - yes Content - yes Teaching tool– yes Clinical tool - yes Research tool - N/A Language - N/A Kirkpatrick Training Evaluation Level: 1, 2, 3 & 4
**2016** **AnandarajahG**[102] Journal Article	● **Education** ● Medicine– Family Medicine ● United States ● English ● N/A	● Residency Program ● Family Medicine Residents ● *N* = 26 (13 study group; 13 comparison group)	The HOPE model was used to teach spiritual history and assessment skills to family medicine residents. *Educational Intervention*: ● A longitudinal residency spiritual care curriculum, integrated into the usual residency curriculum ● This spiritual care (SC) curriculum included several components: a multi-cultural, biopsychosocial-spiritual model of care; spiritual assessment skill training (using HOPE); rounds with clinical chaplains; small group discussions regarding residents’ own clinical experiences; annual spiritual self-care workshop.	*Study goals*: To assess the short-term and long-term effects of this curriculum. *Methods*: ● 10-year longitudinal individual interview qualitative study ● Family medicine residents were interviewed regarding their attitudes and their approach to integrating spiritual care into their medical practice. ● Intervention group was interviewed pre- (as 1st year residents) and post- intervention (as 3rd year residents and again 8 years later). ● Interviews of residents (as 3rd years) who received training were compared to those of a comparison group (as 3rd years) who had not received training.	The HOPE model was an effective component of this curriculum. *Specific study findings*: ● All residents, regardless of personal beliefs or SC training, described patient scenarios in which spirituality played a role. ● Compared to untrained residents SC trained residents described using more nuanced interviewing techniques, & voiced decreased skill-related barriers to SC. ● Intervention physicians 8 years into practice had integrated SC skills into their “toolbox” of clinical skills ● High impact training elements: patient-centered spiritual assessment (HOPE), chaplain rounds, self-care workshops, and a multicultural SC framework.	Acceptability: yes Feasibility: yes Validity: Face - yes Content - yes Teaching tool– yes Clinical tool– N/A Research tool - N/A Language - N/A Kirkpatrick Training Evaluation Level: 1, 2 & 3 Longitudinal Study
**2022** **Thomas AJ**[103] Thesis	● **Education** ● Nursing ● United States ● English ● N/A	● Long Term Care Facility ● Nursing Staff ● *N* = 6	The HOPE model was selected as the spiritual history/assessment training tool for this teaching session *Educational intervention*: ● A PowerPoint training for nursing staff regarding spiritual care. ● Included the HOPE model for spiritual assessment	*Study goals*: To assess the effectiveness of this brief training *Methods*: • A pre/post self-assessment of competency using the 10-item Spirituality Care Competence Scale (SCCS)	The HOPE model was an effective training tool for nurses in this setting *Specific study findings*: ● Pre-assessment scores indicated that nurses did not feel prepared to provide spiritual care to patients. ● There was an increase in SCCS scores following PowerPoint training (including the HOPE model)	Acceptability: yes Feasibility: yes Validity: Face - yes Content - yes Teaching tool - yes Clinical tool– N/A Research tool - N/A Language - N/A Kirkpatrick Training Evaluation Level: 1, 2
**2022** **Kimball SL**[104] Journal article	● **Education** ● Medicine ● United States ● English ● N/A	● A teaching hospital’s Immigrant & Refugee Health Center ● Internal medicine residents ● *n* = 28	HOPE was selected as the spiritual history/assessment training tool *Educational Intervention*: • A clinical chaplain trained internal medicine residents to use 2 screening questions from HOPE for appropriate clinic patients • If the answer to the first question - *“For some people*,* their religious or spiritual beliefs act as a source of comfort and strength in dealing with life’s ups and downs. Is that true for you?”-* is “YES” then residents were instructed to offer a chaplain referral as in the E domain of HOPE.	*Study Goals*: Pilot study to test the feasibility of embedding chaplains in a primary care setting for immigrants, refugees and asylum seekers in Boston *Methods*: • CPE chaplain interns were embedded in the clinic • Internal medicine residents were trained in HOPE questions • If a patient screening resulted in the patient requesting a chaplain visit, the internal medicine resident introduced the patient to the chaplain (a “warm handoff”) • Tracked referrals and outcomes	HOPE is feasible and appears valid as a training tool and a clinical tool. Training internal medicine residents in the 2 screening questions from HOPE appears effective in identifying patients who might benefit from chaplain referral. *Specific study findings*: • 28 patients were screened • 9 met with chaplains and for 6 (67%) spirituality was relevant to their treatment decision-making.	Acceptability: yes Feasibility: yes Validity: Face - yes Content - yes Teaching tool - yes Clinical tool– yes Research tool - N/A Language - N/A Kirkpatrick Training Evaluation Level: 4
**2023** **Bush RS**[105] Journal Article	● **Education** ● Nursing ● United States ● English ● N/A	● Nursing School ● Undergraduate Psychiatric Mental Health Nursing Students ● *N* = 103 (intervention) *N* = 84 (comparison)	The HOPE model was used as to teach spiritual care skills to undergraduate nursing students. Course leaders selected the HOPE model, based on literature review and because “*it is brief*,* easy to use and allows for a broad interpretation of spirituality”.* *Educational intervention*: ● Students were trained on the HOPE model via both didactic and experiential methods, including practice with standardized patients. ● Clinical faculty also received training on using the HOPE model for clinical and educational purposes	*Study goals*: To evaluate the effectiveness of this curriculum *Methods*: ● Comparison of student scores on two clinical competency scales between trained and untrained cohorts. ● Student responses to open-ended questions & reflections about the HOPE model. ● Clinical and simulation faculty feedback	The HOPE model was validated as an effective training tool for undergraduate nursing students. *Specific study findings*: ● The trained cohort scored higher on both competency scales compared to untrained cohort. ● Most students’ feedback was positive. Students cited HOPE as easy to use and “*opened the door to a more in-depth discussion of spiritual needs and provided nursing students an opportunity to speak comfortably with patients and each other about spiritual well-being*.” ● Faculty appreciated the faculty training; reported that students responded well to HOPE model.	Acceptability: yes Feasibility: yes Validity: Face - yes Content - yes Teaching tool - yes Clinical tool– N/A Research tool - N/A Language - N/A Kirkpatrick Training Evaluation Level: 1 & 2
**RESEARCH USES OF THE HOPE MODEL**						
**2005** **Giorgio B**[106] Journal Article	● **Research** ● Undergraduate students ● Australia ● English ● N/A	● University ● Undergraduate Psychology Students ● *N* = 77	● HOPE was one of four instruments used in this study. It was the only qualitative questionnaire amid three quantitative scales ● All four domains of HOPE were used for this study, but only answers to H are presented in this paper.	*Study goals*: to assess how students (undergraduates) view spirituality and derive meaning in their lives. *Methods*: ● Scores on the Spirituality Rating Scale, the Spiritual and Religious Dimensions Scale, and the Spiritual Transcendence scales were summed to form a composite score and entered into SPSS for analysis. ● Handwritten responses to the HOPE questions were collected and underwent thematic analysis.	The HOPE model was effectively used as a qualitative research tool. *Finding from the HOPE questions*: ● locus of internal support for students is external to the self, found in relationship to others ● religious or spiritual beliefs are seen as a source of strength and comfort in 38% of participants ● for most participants, conventional ideas of religion and spirituality lack relevance and meaning.	Acceptability: yes Feasibility: yes Validity: Face - yes Content - yes Teaching tool– N/A Clinical tool– N/A Research tool– yes (qualitative) Language - N/A
**2014** **Gupta PS**[107] Journal Article	● **Research** ● Family Medicine ● United States ● English ● N/A	● Family medicine resident teaching practice ● Patients with diabetes ● *n* = 18	● Several questions for the semi-structured interview guide for this focus group study were adapted from the HOPE model (all 4 domains of HOPE).	*Study Goals*: To explore motivators for diabetes self-management, with a focus on clarifying the role of spirituality as a self-care motivator. *Methods*: ● Focus group study ● Audio-recorded transcripts of the focus groups underwent thematic analysis	HOPE (all 4 domains) was effectively used and adapted for this qualitative focus group study. *Findings from HOPE questions*: ● Diabetes has a significant impact on daily life ● Patients varied in the role spirituality played in their illness, from minimal to profound impact ● All appeared comfortable discussing spirituality in the context of strength and hope	Acceptability: yes Feasibility: yes Validity: Face - yes Content - yes Teaching tool - N/A Clinical tool - N/A Research tool– yes (qualitative) Language - N/A
**2017** **Estupiñan B**[108] Journal Article	● **Research** ● Medicine ● United States ● English ● N/A	● Medical School ● Medical students ● *N* = 146	● Used modified open-ended questions from the HOPE model as a component of their study (all 4 domains of HOPE)	*Study Goals*: To analyze how spiritual well-being may reduce the occurrence of negative burnout symptoms in medical students *Methods*: ● Online survey of medical students ● Quantitative tools: Maslach Burnout Inventory (MBI); WHOQOL- SRPB ● Qualitative tool: modified HOPE questions	HOPE (all 4 domains) was effectively adapted and used as the qualitative component of this study. *General study findings*: • Significant inverse relationship between spirituality and exhaustion/cynicism • Significant positive relationship between spirituality and professional efficacy *Findings from HOPE questions*: • Students most frequently reported family, friends, God/higher power as sources of comfort • Over half reported no affiliation to a religious or spiritual community • 85.6% expressed conflicts between personal beliefs and medical care/ decisions	Acceptability: yes Feasibility: yes Validity: Face - yes Content - yes Teaching tool - N/A Clinical tool - N/A Research tool– yes (qualitative) Language - N/A
**2018** **Jones S**[109] Journal Article	● **Research** ● Mental Health ● Australia ● English ● N/A	● A mental health community support service organization ● Patients with severe mental illness ● *N* = 16	● Authors selected HOPE for this qualitative study because it offers a *“framework in which to explore spirituality and its relationship to their mental health*,* well-being & recovery.”* ● Questions from all 4 domains of HOPE were used plus questions specifically related to relationship with nature.	*Study goal*: to explore the views and experience of spirituality among people living with mental illness in Australia. *Methods*: ● Qualitative individual interview study using the HOPE model as an interview guide ● Responses were analyzed and organized thematically.	HOPE was effectively used as an instrument for this qualitative study. *Findings from HOPE questions*: Three themes emerged: ● concepts of spirituality ● benefits of spiritual practices ● perceived spiritual benefits of recreational and physical activity.	Acceptability: yes Feasibility: yes Validity: Face - yes Content - yes Teaching tool - N/A Clinical tool - N/A Research tool– yes (qualitative) Language - N/A
**2018** **Jones JEH**[110] Thesis - PhD	● **Research** ● Psychology ● United States ● English ● N/A	● Survivor of suicide groups ● Adult survivors of parental suicide ● *N* = 9	● Used questions from the HOPE Model (H, O & P domains) for the semi-structured interview guide for this qualitative study. ● Researcher slightly adapted questions for flow of the interview	*Study Goals*: To assess the impact of a parent’s suicide on the spiritual experience of adult survivors. *Methods*: ● Qualitative individual interviews using an interview guide adapted from the HOPE model ● Interviews were analyzed using an inductive thematic analysis technique	Questions from 3 domains of HOPE were successfully used in the semi-structured interview guide for this qualitative individual interview study *Findings from HOPE questions*: ● Parental suicide causes survivors to question meaning, values and worldview– spiritual suffering ● Many participants did not find the compassion or healing they sought from their religious community ● The stigma of suicide poses barriers to accessing support in healing	Acceptability: yes Feasibility: yes Validity: Face: yes Content: yes Teaching tool: N/A Clinical tool: N/A Research tool: yes (qualitative) Language: N/A
**2022** **Jamil A**[111] Journal Article	● **Research** ● Pharmacy ● United States ● English ● Hindi and Urdu	● University Pharmacy Dept. ● South Asian Immigrant patients with DM type II, CAD, MI or CVD ● *N* = 12	● Used questions from the E domain of HOPE for this qualitative study, along with questions from 2 other models ● Authors slightly adapted questions to focus on medication adherence. ● Translated to Hindi & Urdu.	*Study goal*: to explore how cultural and spiritual beliefs influence medication adherence in this population. *Methods*: ● Qualitative interview study ● Used the E domain of the HOPE model plus questions from the Explanatory Model and the Dimensions of Medication Adherence models.	HOPE was effectively modified for used as an interview guide for a qualitative study. *Finding from HOPE questions*: Participants’ responses suggest that religious/spiritual beliefs may strengthen medication adherence	Acceptability: yes Feasibility: yes Validity: Face - yes Content - yes Teaching tool - N/A Clinical tool - N/A Research tool– yes (qualitative) Language– yes (Hindi & Urdu)
**Di Placido M**[112] 2023 journal article	● **Research** ● Sociology ● Italy ● English ● Italian	● Hospital Heme/Oncology Department ● Oncology Patients ● *n* = 18	● Translated and shortened the HOPE model for a qualitative study of oncology patients ● Used all 4 domains of the HOPE model ● Due to the pandemic the research team trained nurses to conduct the interviews.	*Study goals*: The broader mixed-methods study compares institutional, professionals’ and patients’ perspectives on spiritual care. Part 3 of this project utilizes the HOPE questions, as described below. *Methods*: ● Researchers trained nurses on the use of HOPE for a qualitative interview study ● Nurses conducted 1:1 interviews with oncology patients ● Researchers analyzed interviews	HOPE was successfully translated into Italian and modified for use as a qualitative research tool. *Study findings (from HOPE)*: ● patients were mostly “non-practicing believers” (14/18) ● despite a low level of religiosity, spirituality—broadly defined to include prayer, music and walks in nature—seems to provide comfort and support to most patients. ● patients’ needs seem existential rather than religious/spiritual	Acceptability: yes Feasibility: yes Validity: Face -yes Content– yes Teaching tool– N/A Clinical tool– N/A Research tool– yes (qualitative) Language– yes (Italian)

**Table 6:** Evaluations of the HOPE model for spiritual assessment itself (With or without other SH/SA tools) *N* = 14

Year Author Reference Type	● Type of Use ● Discipline ● Country ● Article Language ● Translations	Specific Setting Population Number	Intervention using HOPE (or How HOPE was used)	Study Method	Main Findings/ Outcomes	Evidence for Acceptability, Feasibility or Validity?
**EVALUATION OF THE HOPE MODEL ALONE**						
**2007** **Reis LM**[113] Journal Article	● **Clinical** ● Genetic Counseling ● United States English ● N/A	● National Society of Genetic Counselors ● random sample of genetic counselors ● *N* = 127	● Authors selected HOPE from among 7 other similar spiritual history/assessment models, based on criteria derived from the literature. ● Reasons for selection: “*This tool is patient-centered*,* utilizes an interactive conversational style*,* introduces the topic of spirituality gradually by beginning with indirect aspects of spirituality*,* provides an opportunity for clients to decline further exploration*,* and is respectful of most religious traditions’ framework of spirituality”*	*Broad study goals*: *“to explore spiritual assessment practices & barriers within genetic counseling.”* *Goals specific to HOPE*: To examine the feasibility of HOPE as a spiritual history/assessment model within genetic counseling *Methods*: ● An emailed on-line questionnaire to genetic councilors ● Questionnaire developed by a multidisciplinary team to ensure content validity. ● Assessed counselors’ perceived relevance of and comfort with questions from each of the four domains of HOPE	*General Findings*: 60% of genetic counselors reported performing a spiritual history/ assessment within the past year, only 8.7% assessed spirituality in more than half of their sessions. *Findings related to HOPE*: ● H questions were rated most relevant (93% found at least 3 of the 4 questions relevant); then E (86%); then O, and P. ● 69.1% said they definitely (22.4%) or might (46.7%) incorporate HOPE questions into patient care ● 65% felt having an spiritual history/assessment tool would increase their ability to elicit information from clients	Acceptability: yes Feasibility: yes Validity: Face - yes Content - yes Teaching tool– N/A Clinical tool– N/A Research tool– N/A Language - N/A
**2010** **Koster**[114] Thesis	● **Clinical** ● Medicine– General Practice (GP) ● Netherlands ● Dutch ● Dutch	● Northern Netherlands GP Training Programs ● GPs– both GP trainers and participants in training programs ● *N* = 9	● HOPE was selected based on literature review. ● Reason for selection: *“Of the three most frequently mentioned models… the HOPE model seems to be the most relevant for the Dutch GP*,* because [it] uses a broad view of meaning*,* has been developed for a routine consultation and makes a clear connection between meaning and the effect of meaning. on [patient care].*	*Broad Study Goal*: To find methods for GPs to increase their awareness of meaning and the effects of meaning for their patients. *Goal relative to HOPE*: can the HOPE model be used to improve communication regarding meaning? *Methods*: • Conducted two 1:1 semi-structured interviews and one focus group with 7 GPs. • Included specific questions regarding the practicality of using HOPE as a communication tool *regarding meaning*.	*General findings*: GPs generally felt that understanding the role of meaning for patients was important *Findings related to HOPE*: ● Mixed findings regarding opinions on the HOPE model as a tool for discussing meaning ● 1:1 interviews: HOPE is a useful guide and can be adapted to GP’s style and patients’ needs. ● focus group: Mixed responses. Some felt it was relevant in the hospital; some felt it “unnatural” to use a scripted set of questions	Acceptability: mixed Feasibility: mixed Validity: Face - yes Content - yes Teaching tool– N/A Clinical tool– N/A Research tool– N/A Language– yes (Dutch)
**2022** **Fopka-Kowalczyk**[115] Journal Article	● **Research & Clinical** ● Medicine ● Poland ● English ● Polish	● Research ● Chronic Illness ● Experts & Patients *N* = 11 experts (doctors, nurses, psychologists, patients’ family); ● *N* = 15 patients (with chronic illness)	● Authors selected HOPE as a potentially viable model for the Polish context, based on a review of the English and Polish literature, ● Reason for selection: *“allows for an open-ended exploration of an individual’s spiritual resources and concerns*”	*Study goal*: *To culturally adapt and validate a qualitative tool to explore patients’ spiritual needs— HOPE* *Methods*: A 6-step method ● preparation/literature review ● forward translation/reconciliation ● backward translation/reconciliation ● validation– evaluation by experts, then patients ● approval by author ● final Polish version of HOPE	● Adaptation of the HOPE model into the Polish language was found to be culturally and linguistically appropriate ● Validated by experts (*N* = 11) ● Validated by patients (*N* = 15) who felt the scale was understandable, useful for other patients. ● HOPE has been validated in Polish for clinical uses and research uses as a qualitative tool.	Acceptability: yes Feasibility: yes Validity: Face - yes Content - yes Teaching tool– N/A Clinical tool– yes Research tool - yes Language - yes (Polish)
**2022** **Whitehead IO**[116] Journal Article	● **Clinical** ● General Practice/ Family Medicine ● England ● English ● N/A	● National Online Survey ● General Practitioners in England *N* = 177	● The HOPE model was selected for this study based in literature review ● Reason for selection: “*provides both a clear structure for novice or uncomfortable practitioners*,* as well as a flexible and open approach for more experienced practitioners. The initial question is an open*,* non-religious one*,* ‘what gives you hope in difficult times?’ The tool is designed to be used flexibly*,* allowing it to be a useful addition to a GP’s consultation skills*,* rather than a box-ticking exercise.”*	*Study Goals*: *“to investigate how comfortable GPs feel discussing spiritual health with their patients*,* and to assess the potential benefit of the HOPE model to overcome barriers”* *Methods*: ● Mixed methods study examining quantitative and qualitative data on barriers, facilitators, and use of the HOPE tool. ● Online survey to GPs in England ● Questions assessed: (1) Attitudes on spiritual history in general; (2) Attitudes towards HOPE (as patient, as provider); (3) Comfort asking spiritual history for 5 clinical scenarios, including if HOPE would be helpful	GPs found the HOPE model to be a generally feasible and acceptable spiritual history/assessement communication model. ● 65% of respondents would be comfortable using the HOPE model with patients ● 77% would be comfortable being asked these questions (HOPE) if they were a patient. ● Strengths of HOPE: starting question is open and inclusive; helpful, especially for those not already comfortable with providing spiritual care. ● Weaknesses of HOPE: the length and GPs preferred free form conversations to structured conversations once started.	Acceptability: yes Feasibility: yes Validity: Face - yes Content - yes Teaching tool– N/A Clinical tool– N/A Research tool– N/A Language - N/A
**EVALUATION OF MULTIPLE SH/SA TOOLS INCLUDING THE HOPE MODEL**						
**2013** **Lucchetti**[33] Journal Article	● **Clinical** ● Medicine ● Brazil ● English ● N/A	● Research ● Systematic Literature Review & ● Expert Evaluation *N* = 3 (experts)	N/A	*Study Goal*: To compare the most commonly used instruments for conducting a spiritual history in the clinical setting. *Methods*: ● Systematic literature review revealed 25 spiritual history/ assessment tools ● 3 authors developed 16 metrics to use in evaluating these tools, based on literature review ● 3 authors independently evaluated all 25 tools based on these 16 metrics, and resolved disagreements until consensus was reached.	*General Findings*: ● Tools with the greatest scores were FICA, SPIRIT, FAITH, HOPE, RCP (13, 12, 12, 11, 11 points) ● Average time to administer each: 4–5 min FICA; 5–6 min HOPE & FAITH; 10–15 min SPIRIT; 20–25 min RCP) *Findings specific to HOPE*: ● Authors deducted 1 point for “lack of validation” and 1 point for “dealing with terminal events” (However, HOPE’s E domain covers **E**ffects & **E**nd-of-Life care and HOPE had been previously validated as a teaching tool [99,100, 101]) *Authors conclude*: Use of each tool must be individualized, to setting, profession, time, patient profile.	Acceptability: yes Feasibility: N/A Validity: Face - yes Content - yes Teaching tool– N/A Clinical tool– N/A Research tool– N/A Language - N/A
**2013** **Pennaertz R**[117] Thesis	● **Clinical** ● Palliative care ● Netherlands ● Dutch ● N/A	● Research ● Literature Review & ● Expert Evaluation *N* = 1 (expert)	N/A	*Study goals*: To identify spiritual history/assessment tools suitable for use by primary care clinicians in palliative care in Netherlands *Methods*: ● Literature review revealed 32 tools that underwent further analysis ● Applied 7 criteria, based on the work of Joep Van de Geer, re: suitability for the Dutch context ● Tools were additionally evaluated using ABC model (Attention, Guidance, Crisis Intervention)	*General findings*: 5 spiritual history/assessment tools met all 7 criteria (SPIRIT, HOPE, FACT, FAITH, 5 dimensions); 1 met 6/7 criteria (FICA). All 6 tools are appropriate for the Dutch context. *Findings specific to HOPE*: ● Meets all 7 criteria & strong ABC ● Strengths: easy to remember; asks about effects on medical care and last phase of life (only in a few other tools). ● Weakness: some confusion regarding “source of strength”	Acceptability: yes Feasibility: N/A Validity: Face - yes Content - yes Teaching tool– N/A Clinical tool– N/A Research tool– N/A Language– Yes (Dutch)
**2013** **Piotrowski LF**[118] Journal Article	● **Clinical** ● Medicine– Palliative Care ● United States ● English ● N/A	● Academic Medical Center ● All members interdisciplinary palliative care team (nurses, chaplains, social workers, physicians, fellows, nurse practitioners, students, etc.) ● *N* = 374 (Part 1) ● “all members of the inter-disciplinary team” (for Part 2)	● HOPE was one of several spiritual history/assessment models systematically evaluated for use as the screening tool at their institution. ● HOPE was ultimately selected and implemented as the screening tool routinely used on admission	*Project Goals*: To improve the quality of spiritual care provided to palliative care patients. *QI Project Part 1*: Needs assessment and pilot spiritual history training *QI Project Part 2*: Systematically evaluated 5 tools for use in their setting (FICA, HOPE, SPIRIT, Spiritual Cat Scan and Spiritual Pain Assessment Form). *Methods*: ● detailed presentation of each tool ● input from all team members ● all information reviewed by a sub-committee ● subcommittee recommendations reviewed by entire team with further discussion and input ● consensus regarding tool choice.	*Project findings*: Consensus reached on employing the HOPE model + 1 extra question “Are you at peace?” as the spiritual screening tool that best fit their setting. *Resulting actions related to HOPE*: ● Began implementing spiritual care screening, using HOPE with documentation in the EHR. ● A chaplain reviews these screens and arranges follow-up with appropriate patients for a full evaluation and spiritual care plan.	Acceptability: yes Feasibility: yes Validity: Face - yes Content - yes Teaching tool– N/A Clinical tool– yes Research tool– N/A Language - N/A
**2014** **Wijker DDE**[119] Thesis	● **Clinical** ● Medicine (hospital setting) ● Netherlands ● Dutch	● Research ● Literature Review ● Survey of spiritual care providers (*N* = 82) ● Expert Evaluation of spiritual assessment tools (*N* = 1 expert)	N/A	*Study Goal*: to improve the assessment of patients’ spiritual care needs in the hospital setting. Part 1: Survey of spiritual caregivers Part 2: Evaluation of spiritual history/assessment tools *Methods*: ● Literature review: identification of key concepts and available tools. ● Survey of spiritual care providers regarding current practice ● Evaluated 7 tools using structured criteria, based on literature review (included clinical and research specific criteria): HOPE, Fitchett’s 7 × 7, SPIRIT, FICA, Lastmeter, FACITsp and Nijmeegs Model.	*General Finding*: ● Survey– almost no providers used structured spiritual assessment tools; most preferred a narrative approach; training is needed. ● Evaluation of tools based on 8 criteria - Author concludes that Lastmeter and Nijmeegs are most promising for the Dutch context. (FACITsp & HOPE met 4/8 criteria and FICA, SPIRIT and 7 × 7 met 3/8 criteria) *Findings Specific to HOPE*: ● “*The model is beneficial as it enables a neutral conversation starter” (for diverse patients)* ● *Needs testing in Dutch context*	Acceptability: yes Feasibility: N/A Validity: Face– yes Content– yes Teaching tool– N/A Clinical tool– N/A Research tool– N/A Language– N/A
**2015** **Blaber M**[34] Journal Article	● **Clinical** ● Palliative Care ● England ● English ● N/A	● Palliative Care ● Systematic Expert Evaluation ● *N* = 3 experts	N/A	*Goals*: To evaluate four of the more widely used spiritual history taking tools for suitability in palliative care settings. *Methods*: ● utilized criteria found on literature review re: qualities of effective spiritual history/assessment tools. ● applied these criteria to the top 4 rated tools in the article by Lucchetti et al., (2013) - FICA, SPIRIT, HOPE, FAITH ● Assessed the suitability of each tool for palliative care, detailing strengths and weaknesses per tool.	*Overall findings*: Found the HOPE model to *“most comprehensively reflect the healthcare literature in exploring the various elements of spirituality known to be important to a person’s wellbeing at the end of life.”* *Other strengths*: memorable; acceptable; normalizing introductions; ability to be individualized; exploration of formal/informal beliefs, range of spiritual practices, and end of life. *Weakness*: not previously validated, possible cultural bias, time	Acceptability: yes Feasibility: N/A Validity: Face - yes Content - yes Teaching tool– N/A Clinical tool– N/A Research tool– N/A Language - N/A
**2017** **Memaryan N**[120] journal article	● **Research** ● Mental Health ● Iran ● English ● Farsi?	● Interdisciplinary Expert Panel ● *N* = 11 (first Delphi round) ● *N* = 10 (second Delphi round)	N/A	Used in a Delphi study to develop a spiritual history/assessment tool for the Islamic population *Methods*: a 3-phase process ● Phase 1 & 2– a 6-member expert panel agreed upon a conceptual framework and reviewed the literature regarding tools. ● Phase 3– the panel selected 33 questions, from 4 tools (BELIEF, FICA, HOPE, and SPIRIT) for in-depth assessment. ● 33 questions underwent a 2-phase Delphi process of content validation by consensus of 11 experts with focus on relevance, simplicity and clarity.	*Outcomes*: ● Consensus on a validated set of spiritual history/assessment questions for use with the Islamic population in Iran. ● The final 16 validated questions included: − 7 questions from HOPE − 4 questions from FICA − 3 questions from SPIRIT − 3 questions from BELIEF *Findings Specific to HOPE*: Had the most number of questions included in the final 16 validated questions (see above)	Acceptability: yes Feasibility: N/A Validity: Face- yes Content- yes Teaching tool- N/A Clinical tool- yes Research tool– N/A Language -?
**2021** **Brown P**[121] Thesis	● **Education** ● Social Work ● United States ● English ● N/A	● Bachelor of Social Work (BSW) and Master of Social Work (MSW) training programs ● Social Work Educators ● *N* = 145	N/A	*Study Goals*: Assess SW educators’ opinions regarding what content should be included in a spiritual care curriculum and their familiarity and/or use of common spiritual history/assessment (SH/SA) tools. *Methods*: ● Literature review to identify common SH/SA tools. ● Online survey of social work (SW) educators to assess familiarity and use of tools (FICA, HOPE, FAITH, SPIRIT, CSI-MEMO)	*Overall Findings*: ● Most SW educators believed spiritual care should be included in SW curriculum. ● Most were unfamiliar with the most common tools (77%). ● Those who were familiar with SH/SA tools were most familiar with SPIRIT, FICA & HOPE. ● Most SW curricular did not include SA/SH tools (76%). Of those that did, the most commonly taught were FICA and HOPE. *Findings Specific to HOPE*: One of the most familiar and most commonly taught SH/SA tools	Acceptability: N/A Feasibility: N/A Validity: Face - yes Content– N/A Teaching tool– N/A Clinical tool– N/A Research tool– N/A Language - N/A
**2021** **Jones KF**[122] Journal Article	● **Education** ● Healthcare professionals ● Australia ● English ● N/A	● Australia (Nationwide) ● Experts in spiritual care who work in a healthcare field. ● *N* = 107	N/A	*Study goals*: To determine components to be included in spiritual care training for healthcare professionals, including spiritual history/assessment tools. *Methods*: ● 3 rounds of a Delphi study ● Round 1– participants listed: most important curricular elements; preferred teaching methods; clinical scenarios for training; current spiritual assessment and referral procedures ● Rounds 2 & 3– Responses were analyzed and presented for participants to rank ● Consensus achieved if > 75% rated item as “desirable” or “essential”	*General Findings*: ● Consensus achieved on several components for training. ● Highest ranked: “screening all patients for spiritual concerns” ● Consensus also achieved on who should screen: “all members of the healthcare team” *Findings specific to HOPE*: ● Round 1 - most common tools: FICA, HOPE, SPIRIT, FAITH. ● Rounds 2 - participants ranked: HOPE (44.7%) and FICA (43.4%) the highest as “desirable” or “essential” for training. However, 40% of participants were not familiar with either tool. ● No consensus re: tools	Acceptability: yes Feasibility: N/A Validity: Face - yes Content - yes Teaching tool– N/A Clinical tool– N/A Research tool– N/A Language - N/A
**2022** **de Queiroz CM**[123] Journal Article	● **Clinical** ● Medicine (nonspecific) ● Brazil ● Portuguese	● Research ● Literature Review & ● Expert Evaluation *N* = 7 experts	N/A	*Study Goal* Identify spiritual assessment tools for clinical setting & evaluate these tools. *Methods*: ● Literature review– database search in English and Portuguese ● Evaluated each spiritual history/assessment tool and validated them based on 8 inclusion criteria and 8 exclusion criteria.	*General Findings*: Identified 7 spiritual history/assessment tools that met their validation criteria: Kuhn Spiritual Inventory; Matthews Spiritual History; FICA; SPIRIT; HOPE; ACP Spiritual History; CSI: Memo *Finding Specific to HOPE*: ● *“addresses patients’ spirituality in a very general way*,* enabling them to discuss their most intimate beliefs*,* regardless of whether they follow an organized religion”* ● *“the only questionnaire that seeks to find out whether the disease interfered/changed patient’s beliefs*	Acceptability: yes Feasibility: N/A Validity: Face - yes Content - yes Teaching tool– N/A Clinical tool– yes Research tool– N/A Language– N/A
**2023** **Campbell D**[124] Journal Article	● **Clinical** ● Nursing ● United States ● English ● N/A	● Magnet teaching hospital in the Midwest ● Patients in the Medical-Surgical and Telemetry Unit ● *N* = 101	N/A	*Study Goal* Develop a concise quantitative spiritual screening instrument for hospital use. *Methods*: • Literature review to identify common spiritual history/assessment tools • Researchers selected 10 of the most relevant questions from HOPE, SPIRIT, FICA, and FAITH • Researchers created a survey for patients to: (1) select top three questions; (2) evaluate each question on the 1–4 Likert scale; (3) suggest any additional helpful questions • Researchers created a 4-question quantitative screening tool based on the most highly rated questions	*General Findings/Outcomes*: • Incorporated the new 4-item screening tool into the EMR, triggering Pastoral Care referrals • Screening resulted in an increase in chaplain referrals from 1,305 in 2011 to 10,294 in 2019 (689% increase) *Finding Specific to HOPE*: • 1 question, unique to HOPE, received highest Likert score (3.29); and highest % of patients including it in their top 3 questions (60%) *(“For some people*,* their religious or spiritual beliefs act as a source of comfort and strength in dealing with life’s ups and downs. Is this true for you?”)* • 1 question, unique to HOPE had second highest % of patients selecting it as one of their top 3 (50%); not selected for the screening tool because it is qualitative. *(“What is there in your life that gives you internal support*,* or what sustains you and keeps you going?”)* • All 4 final questions were adaptions of questions from HOPE (3 also seen in other tools)	Acceptability: yes Feasibility: N/A Validity: Face - yes Content - yes Teaching tool– N/A Clinical tool– yes Research tool– N/A Language– N/A

Some sources point to its length as a weakness of HOPE, while others note that it is a flexible tool. A shorter version of HOPE, guided by the most frequently used/selected questions, may be helpful in some settings (see Table 1; Table 3). Several sources mention lack of validation as a concern about HOPE and other spiritual history/assessments (see validation section below).

#### Clinical applications of HOPE

12 articles provided descriptions of clinical applications of HOPE (Tables-4,5) with 9 selecting HOPE as the only spiritual history/assessment tool. Four provided case-reports [69–72]. Three report positive effects of using HOPE routinely for all patients admitted to their clinical service [64, 66, 96] and 2 used HOPE as a clinical standard for study inclusion [97, 98]. Three used HOPE, with other tools, to create a new tool [65, 67, 68]. This variety of successful uses speak to the versatility of this communication model. However, only 3 studies formally evaluated effects of using HOPE clinically [96–98], leaving room for further study.

#### Educational uses of HOPE

Of the 16 articles describing educational uses of HOPE,[73–81,99−105] 9 selected HOPE as the only spiritual history/assessment teaching tool [76, 77, 99–105] and 7 included formal evaluation of HOPE [99–105]. All 7 moved beyond simple feedback evaluation (Kirkpatrick Level 1) to measures of knowledge/skill acquisition, attitudinal/confidence change, practice change and institutional-level quality measures (Kirkpatrick Levels 2–4) [113]. Together these studies provide compelling evidence for the effectiveness of HOPE as a training tool for nurses, medical students, and medical residents. All studies were in English, therefore applicability to non-English speaking cultures is unknown.

#### Research uses of HOPE

Nineteen studies used or adapted HOPE as an instrument for their research study.[82–93,106−112] Twelve adapted isolated questions from HOPE for quantitative (*N* = 5), qualitative (*N* = 5) or mixed methods (*N* = 2) studies [82–93]. The 7 studies that used all 4 domains of HOPE effectively applied it as a qualitative research-instrument [106–112]. These findings are consistent with HOPE’s developed purpose as a patient-centered communication tool.

#### Acceptability, feasibility and validity of the HOPE model

A common concern about spiritual history/assessment tools is perceived lack of validity, despite their function as communication tools, rather than quantitative instruments. This scoping review revealed several studies supporting acceptability, feasibility and validity of HOPE.

The 266 articles in which authors independently offered opinions/evaluations of HOPE provide strong evidence for face validity (Table 2; Supplemental Table-1, Table-3). Of the 63 sources providing specific uses and/or evaluations of HOPE (Tables-4,5,6), 59 explicitly assessed and/or demonstrated acceptability, 34 feasibility, and 30 content validity.

Of the 31 studies/evaluations (Tables-5,6), 21(68%) were of HOPE alone and 10 (32%) evaluated HOPE plus other tools. Of the 17 “HOPE only” intervention studies (Table 5), 6 demonstrated its validity as a clinical tool, 7 as an educational tool, and 7 as a qualitative research instrument. Of the 14 studies evaluating the HOPE tool itself (Table 6), 4 studied HOPE alone and 10 compared spiritual history/assessment tools. Each study utilized different evaluation criteria. 13/13 specifically assessed for and demonstrated HOPE’s content validity, 12/13 acceptability (1 mixed results) and 4/5 feasibility (1 mixed results).

While together these findings are compelling, studies are very heterogeneous. Therefore, targeted research may be needed for specific situations.

#### Language and cultural adaptability of HOPE

16.5% of 571 sources mentioning HOPE and 15.1% of 266 offering opinion/evaluation of HOPE were published in non-English languages (Table 2). Of these 266 articles, 13 included a full-version translation of HOPE (4-Portuguese [90, 120, 126, 127], 4-Spanish [128–131], 1-German [131], 2-French [96, 131], 1-Dutch [119], 1-Polish [116]). Two studies describe translating HOPE (Italian [112], Hindi and Urdu [111]), but do not include translations. One study conducted a formal validation of HOPE (full-version) in Polish [116]. 

Seventeen articles described specific applications and/or evaluation of HOPE in non-English speaking countries (Tables-4,5,6). While several authors discuss HOPE’s suitability for diverse and/or secular settings, few studies have tested this hypothesis. Therefore, while HOPE appears feasible in non-English settings, more study is needed.

#### Uses of HOPE as a spiritual screening, history and/or assessment tool

Consensus in hospice/palliative care is that there should be a clear distinction between “spiritual screening”, “spiritual history” and “spiritual assessment”. Spiritual screening and history-taking may be performed by all clinical staff (primary spiritual care providers). However, spiritual assessment, resulting in a spiritual care plan, should be conducted by spiritual care specialists [46]. Additionally, while spiritual screening and history-taking may involve distinct question, spiritual assessment requires a conversation and is an ongoing process [132, 133]. 

This scoping review revealed evidence that HOPE is being successfully used for spiritual screening, history-taking and assessment. The way it is used depends on clinical setting, clinician expertise, and access to spiritual care specialists. For example, when spiritual care specialists are readily available (e.g. hospice/palliative care, hospital), the clear separation of roles described above is appropriate and HOPE’s use as a screening or history-taking tool improves chaplain referral rates [96, 97, 101, 104]. In educational settings with novice clinicians [76, 99–101, 103, 105], and in qualitative research [82, 86, 88, 91, 109, 112], HOPE is most often used as a history-taking/data-gathering tool.

However, in settings where access to spiritual care specialists is limited (e.g. outpatient primary care), experienced clinicians have used HOPE as an assessment tool to develop holistic treatment plans that incorporate consideration of patients’ spiritual health needs [36, 69–71, 102]. Whitehead notes, in her study of HOPE’s acceptability and feasibility for general practice physicians, that “the tool [HOPE] is designed to be used flexibly, allowing it to be a useful addition to a GPs consultation skills, rather than a box-ticking exercise.” [117] These findings are consistent with a recent study of family physicians’ stories regarding spirituality and patient care, which demonstrates that spiritual care in this setting includes longitudinal relationships, conversational spiritual assessments, shared decision-making regarding treatment options, detecting spiritual suffering or distress, and determining the most appropriate spiritual care specialist referral and/or community resource for each unique patient [134]. 

Additionally, some sources describe HOPE’s use as a simple spiritual assessment model for spiritual care specialists. While HOPE does not provide the depth that tools designed specifically for clinical chaplains cover (e.g. 7 × 7 model) [40], some chaplains use HOPE as a structure for organizing initial exploration of the 4 HOPE domains [98, 135, 136]. 

Finally, one source described utilizing HOPE as a self-assessment tool for learners [77]. Since the pandemic, the spiritual well-being of healthcare professionals has been receiving increasing attention, with studies showing an association between poor spiritual health and risk for burnout [137]. Therefore, further study regarding the usefulness of HOPE as a spiritual self-understanding, self-awareness and self-care tool for both trainees and experienced clinicians is warranted. Increased spiritual self-awareness may also help clinicians become more aware of beliefs and biases that might interfere with their provision of patient-centered care [138]. 

### What this study adds

Contribution to existing literature: (1) To our knowledge, this is the first scoping review assessing interest, applications, feasibility and validity of any of the well-known spiritual history/assessment tools; (2) It provides evidence regarding acceptability and feasibility of the HOPE model; (3) It revealed studies demonstrating HOPE’s content validity and its validity as a communication tool (versus quantitative instrument), in clinical, educational and qualitative research settings; (5) It identifies opportunities for future research and systems improvements.

### Strengths and limitations of the study

This study has several strengths. It followed an established methodology for scoping reviews. Researchers conducted full-text analysis of 909 articles, resulting in a comprehensive view of international literature regarding HOPE. The tiered article selection and analysis approach revealed both breadth of interest and specific applications/evaluations of HOPE. Rubrics, templates, and regular group analysis meetings facilitated consistency in data extraction and analysis and the ability of researchers to reach consensus regarding findings.

Limitations include that we were only able to examine one spiritual history/assessment tool in depth. While other tools exist, comprehensively accessing studies on all tools was not feasible. However, since HOPE is one of the most common spiritual history/assessment tools [33, 34], we believe we captured most studies that compared tools. Additionally, while we included non-English papers, we recognize that translation limitations may have prevented capturing nuances.

## Summary and future directions

Though this scoping review demonstrates global interest, across multiple disciplines, in the HOPE model, there remains discordance between patients’ desires for spiritual care and clinicians’ spiritual care provision [139]. Previous studies cite lack of comfort, training, and time as clinician barriers to assessing patients’ spiritual needs [42, 43, 140]. A potential additional barrier, preventing more widespread use of spiritual history/assessment tools, is the perception that they have not been tested in clinical, educational or research settings.

This scoping review revealed 266 articles in which authors provided unsolicited, generally positive opinions about the HOPE model. Of these, 63 described specific uses and/or evaluations and underwent in-depth analysis. While there is great heterogenicity in these articles, together they provide evidence of acceptability and feasibility in a variety of settings, with several studies demonstrating content validity and validity in specific settings (e.g. education).

While this scoping review focuses on HOPE, there are other spiritual history/assessment tools available. Studies comparing these,[33–34,117−124] suggest that several could be equally effective in improving communication. Apart from HOPE, the most commonly mentioned are SPIRIT [37] and FICA [35]. This scoping review reveals HOPE’s particular strengths as including its acceptability for diverse (religious/secular/multicultural) patient populations, its flexibility and its adaptability. Fitchett [132] and Cadge [32] both recommend that future directions in spiritual assessment focuses on enhanced use of existing tools rather than creating new tools. Our scoping review supports their recommendations.

This review revealed that HOPE is being used as a spiritual assessment and shared decision-making tool in settings where spiritual care specialist are not readily available. This highlights the different levels of spiritual care skills needed by primary spiritual care clinicians in different settings. There is consensus that referral to a spiritual care specialist is needed for such things as in-depth spiritual assessment, spiritual counseling, addressing theological concerns, and/or ritual needs [46]. However, some therapeutic interventions can be undertaken by primary spiritual care providers, such as providing presence and listening, modifying the medical treatment plan taking into account patients’ spiritual beliefs/needs, or referring patients to a spiritual care specialist [36, 138, 141]. Consensus exists regarding spiritual care competencies for some non-palliative care fields (e.g. family physicians and nurses/midwives).[140,141] However, further clarity is needed regarding appropriate boundaries of role, training and responsibility for a variety of other clinical specialties and settings.

This finding that clinical setting impacts how the HOPE model is used also demonstrates the importance of creating systems that support including spiritual care in patient-centered, whole-person medical care. Even if clinicians are trained in HOPE or other spiritual history/assessment tools and are comfortable communicating with their patients, they will be unable to comprehensively address their patients’ unique spiritual needs without appropriate systems in place that support both primary and specialty spiritual care. Additionally, systems need to support the spiritual wellbeing of healthcare providers, in order to prevent clinician burnout and the resulting negative effects on patients.

Recommended next steps:

1. Dissemination of knowledge regarding HOPE and other validated spiritual history/assessment communication tools.
2. Dissemination of evidence of acceptability, feasibility, and validity to decrease barriers to utilizing existing spiritual history/assessment tools.
3. Additional research examining applications and/or adaptions of the HOPE model, for specific settings, including its possible usefulness as a self-assessment and self-care tool.
4. Additional research examining HOPE in different cultures/languages, to better serve diverse patient populations.
5. Increase routine spiritual history/assessment training in educational settings (e.g. medical school, residency, nursing, social work, psychology), using HOPE and other tools.
6. Clarify competencies for primary spiritual care providers in a variety of clinical fields, with particular attention to appropriate boundaries of role and training.
7. Create systems to support clinicians in their efforts to provide whole-person care [10], such as: creating interdisciplinary teams that include spiritual care specialists (e.g. CPE trained clinical chaplains) in outpatient as well as inpatient settings; compiling referral resources for spiritual care specialists when they are not already part of the team; cultivating community resources tailored to the needs of the local patient population (e.g. religious organizations, indigenous healers, yoga/meditation groups, nature lovers’ groups); and advocating for reimbursement structures that support both primary and specialty spiritual care as part of whole-person care.

## Conclusions

Evidence supports the classification of spiritual well-being as a determinant of health [2, 11]. However, clinicians continue to disregard patients’ spiritual needs, in part due to discomfort discussing the topic and lack of consolidated evidence regarding the effectiveness of utilizing spiritual history/assessment tools designed to facilitate these conversations. This is the first systematically constructed review of any of the well-known spiritual history/assessment tools created for non-specialists in spiritual care.

This scoping review revealed that in the 24 years since its first publication [36], the HOPE model for spiritual assessment has garnered widespread interest, spanning numerous countries, languages, and disciplines. We found 63 sources that described specific uses and/or evaluations of HOPE, including 31 studies demonstrating HOPE’s content validity and/or validity as a clinical, educational or qualitative research tool. However, studies are heterogeneous, providing opportunities for further research. HOPE’s strengths compared to other similar tools include its opening questions, which are broadly inclusive of secular and multicultural religious approaches to spirituality, its adaptability across clinical settings and its flexibility for use by novice and expert clinicians.

Clarifying how to best communicate with patients and families from diverse backgrounds regarding their spiritual care needs during times of suffering, illness and death and creating systems that support this type of care are critical next steps in our ability to provide comprehensive, patient-centered, healthcare to our patients.

## Electronic supplementary material

Below is the link to the electronic supplementary material.

Supplementary Material 1

Supplementary Material 2

## Data Availability

The dataset used and/or analyzed during the current study is available from the corresponding author on reasonable request.
