# Supplementary material for: Evaluation of the HOPE spiritual assessment model: a scoping review of international interest, applications and studies over 20+ years
Source: BMC Palliat Care. 2025 Jul 7;24:191. doi: 10.1186/s12904-025-01809-z (PMC12236049; doi:10.1186/s12904-025-01809-z)
Supplement: Supplementary file 1 — Supplementary Material 1 [file 12904_2025_1809_MOESM1_ESM.docx]

**Supplemental Table 1 – Sources in Which Authors Offer Some Assessment of the HOPE Model (N=266)**

| **Author** | **Year** | **Full Citation** | **Accessed date** | **Non-English Language Article?** | **HOPE Translated?** |
| --- | --- | --- | --- | --- | --- |
| **Abbas SQ, Dein S.** | **2011** | S.Q. Abbas & S. Dein (2011) The difficulties assessing spiritual distress in palliative care patients: a qualitative study, Mental Health, Religion & Culture. 2011; 14(4): 341-352.  DOI: 10.1080/13674671003716780  https://doi.org/10.1080/13674671003716780 | 8/17/24 |  |  |
| **Abuchaim SCB** | **2018** | ABUCHAIM, SCB. Espiritualidade/religiosidade como recurso terapêutico na prática clínica: concepção dos estudantes de graduação em medicina da Escola Paulista de Medicina. Spirituality/religiosity as a therapeutic resource in clinical practice: conception of undergraduate medical students at Escola Paulista de Medicina. 2018. Dissertation (Master’s Degree). Paulista School of Nursing, Federal University of São Paulo. [*https://repositorio.unifesp.br/items/506e4e6c-7ff7-4273-b46d-c0c7e3ae9208*](https://repositorio.unifesp.br/items/506e4e6c-7ff7-4273-b46d-c0c7e3ae9208) | *8/17/24* | Portuguese | **Yes**  **Full Version (almost)**  **Portuguese** |
| **Adanikin A** | **2014** | Adanikin, A.I., Onwudiegwu, U. & Akintayo, A.A. Reshaping maternal services in Nigeria: any need for spiritual care?. *BMC Pregnancy Childbirth* **14**, 196 (2014). <https://doi.org/10.1186/1471-2393-14-196> | 8/17/24 |  |  |
| **Alch KC** | **2020** | [Alch, CK, et al. “Addressing Spiritual and Religious Needs in Advanced Illness A Teachable Moment.” JAMA INTERNAL MEDICINE, vol. 181, no. 1, Jan. 2021, pp. 115–16, https://doi.org/10.1001/jamainternmed.2020.6564. WOS:000593324200002.](https://doi.org/10.1001/jamainternmed.2020.6564) | 8/17/24 |  |  |
| **Anandarajah** | **2016** | Anandarajah et al., “A 10-Year Longitudinal Study of Effects of a Multifaceted Residency Spiritual Care Curriculum: Clinical Ability, Professional Formation, End of Life, and Culture.”J Pain Symp Manage. 2016;52(6):859-872. http://dx.doi.org/10.1016/j.jpainsymman.2016.06.006 | 8/17/24 |  |  |
| **Anderson J** | **2015** | [Anderson, J. Hope and Strengths within Adaptive Sailing: Narratives from the Queen’s Quay Disabled Sailing Program. //, https://scholar.uwindsor.ca/cgi/viewcontent.cgi?article=6303&context=etd.](https://scholar.uwindsor.ca/cgi/viewcontent.cgi?article=6303&context=etd) | 8/17/24 |  |  |
| **Anderson LS** | **2013** | [Anderson, L. S., and L. A. Heyne. “A Strengths Approach to Assessment in Therapeutic Recreation.” Therapeutic Recreation Journal, //, https://www.bctra.org/wp-content/uploads/tr_journals/3873-13443-1-SM.pdf.](https://www.bctra.org/wp-content/uploads/tr_journals/3873-13443-1-SM.pdf) | 8/17/24 |  |  |
| **Attar M** | **2009** | Attar MA, et al. Pediatric Residents’ Competency in Communicating Bad News and Eliciting Spiritual Needs: Development and Evaluation of a Skills-Based Curriculum. J Neonatal-Perinatal Med. 2010; 3:177-185. DOI 10.3233/NPM-2010-0113.  <https://www.researchgate.net/publication/286550054_Pediatric_residents'_competency_in_communicating_bad_news_and_eliciting_spiritual_needs_Development_and_evaluation_of_a_skills-based_curriculum> | 8/17/24 |  |  |
| **Ayvaci ER** | **2017** | [Ayvaci, E. R. “Religious Barriers to Mental Healthcare.” American Journal of Psychiatry Residents’ …, //, https://doi.org/10.1176/appi.ajp-rj.2016.110706.](https://doi.org/10.1176/appi.ajp-rj.2016.110706) | 8/17/24 |  |  |
| **Barbosa ACP** | **2022** | Barbosa, A. C. P. ABORDAR A ESPIRITUALIDADE/RELIGIOSIDADE EM CONSULTA: O PAPEL DA FORMAÇÃO EM COMUNICAÇÃO CLÍNICA SOB A PERSPETIVA DOS …. //, <https://repositorio-aberto.up.pt/bitstream/10216/147894/2/609266.pdf>. | Accessed  1/11/25 | Portuguese | No |
| **Barnhill JW** | **2019** | [Barnhill, J. W. Approach to the Psychiatric Patient: Case-Based Essays. books.google.com, 2018, https://books.google.com/books?hl=en&lr=&id=jAd_DwAAQBAJ&oi=fnd&pg=PT12&ots=NtMmi36RfD&sig=6HjsKL4LQK9uhTasLmkUvSY53oU.](https://books.google.com/books?hl=en&lr=&id=jAd_DwAAQBAJ&oi=fnd&pg=PT12&ots=NtMmi36RfD&sig=6HjsKL4LQK9uhTasLmkUvSY53oU) | 8/17/24 |  |  |
| **Basic J** | **2015** | [Bašić, J. Percepcija Duhovnosti in Duhovne Oskrbe Med Študenti Zdravstvene Nege. //, https://repozitorij.upr.si/IzpisGradiva.php?id=10347.](https://repozitorij.upr.si/IzpisGradiva.php?id=10347) | 1/15/25 | Slovanian | **Yes.**  **Full Version Slovanian** |
| **Beki F** | **2021** | *Bekİ, F. “Sosyal Hizmette Manevi Değerlendirme Yöntemleri ve Ölçekleri.” Toplumsal Politika Dergisi, //.* | *Accessed 9/7/24* | Turkish | No |
| **Besson J** | **2017** | [Besson, J. “Santé, Spiritualité et Addiction.” Psychotropes, //, https://www.cairn.info/revue-psychotropes-2020-1-page-73.htm.](https://www.cairn.info/revue-psychotropes-2020-1-page-73.htm) | Accessed 9/7/24 | French | Brief description; not translation |
| **Blaber M** | **2015** | [Blaber, M., et al. “Spiritual Care: Which Is the Best Assessment Tool for Palliative Settings?” International Journal of Palliative …, //, https://doi.org/10.12968/ijpn.2015.21.9.430.](https://doi.org/10.12968/ijpn.2015.21.9.430) | Accessed 9/14/24 |  |  |
| **Blesch P** | **2013** | [Blesch, P. S. “Spirituality in Nursing Education: Preparing Students to Address Spiritual Needs,” //. https://search.proquest.com/openview/e8ce4f181f3a87446a5eba48169499f5/1?pq-origsite=gscholar&cbl=18750.](https://search.proquest.com/openview/e8ce4f181f3a87446a5eba48169499f5/1?pq-origsite=gscholar&cbl=18750) | Accessed 9/20/24 |  |  |
| **Blum D** | **2014** | [Blum, D. L’accompagnement Spirituel Religieux En Unité de Soins Palliatifs: Effet d’un Entretien Spirituel Sur Le Recours à l’aumônerie.](https://hal.science/dumas-01095976/document)  <https://dumas.ccsd.cnrs.fr/dumas-01095976v1/file/2014GRE15121_blum_denis(1)(D).pdf> | Accessed 1/22/25 | French | **Yes**  **Full Version**  **French** |
| **Bond R** | **2020** | [Bond, R., and NS Brown. “Testing of a (Spiritual) Self-Assessment Tool ((S)SAT) in a Community Hospital Setting.” JOURNAL OF PASTORAL CARE & COUNSELING, vol. 74, no. 4, Dec. 2020, pp. 229–33, https://doi.org/10.1177/1542305020949443. WOS:000592368100003.](https://doi.org/10.1177/1542305020949443) | Accessed 9/20/24 |  |  |
| **Borneman T** | **2010** | Borneman, T., et al. “Evaluation of the FICA Tool for Spiritual Assessment.” JOURNAL OF PAIN AND SYMPTOM MANAGEMENT, vol. 40, no. 2, Aug. 2010, pp. 163–73, [https://doi.org/10.1016/j.jpainsymman.2009.12.019.](https://doi.org/10.1016/j.jpainsymman.2009.12.019) WOS:000281272200001. | Accessed 9/21/24 |  |  |
| **Borragini-Abuchaim S** | **2021** | [Borragini-Abuchaim, S, LG Alonso, and RL Tarcia. “Spirituality/Religiosity as a Therapeutic Resource in Clinical Practice: Conception of Undergraduate Medical Students of the Paulista School of Medicine (Escola Paulista de Medicina)-Federal University of Sao Paulo (Universidade Federal de Sao Paulo).” FRONTIERS IN PSYCHOLOGY 12 (December 24, 2021). https://doi.org/10.3389/fpsyg.2021.787340.](https://doi.org/10.3389/fpsyg.2021.787340) | Accessed 9/20/24 |  |  |
| **Bostanci A** | **2012** | [Bostanci, A., et al. “Clinical Tools to Assist with Specialist Palliative Care Provision.” Melbourne (Australia): St …, //,](http://pcv.trueserver.com.au/wp-content/uploads/2015/11/Clinical-tools-to-assist-with-palliative-care-provision-Nov-2012.pdf) <https://cpccentreforpallcare.blob.core.windows.net/assets/pages/DOH%20Clinical%20Tools%20Report%20Nov%202012(2).pdf> | Accessed 9/21/24 |  |  |
| **Brady S** | **2007** | *Brady, S., and W. Proctor. Pain Free for Life: The 6-Week Cure for Chronic Pain--Without Surgery Or Drugs. books.google.com, 2007,* [*https://books.google.com/books?hl=en&lr=&id=Ru3llwHaD_AC&oi=fnd&pg=PT3&ots=TFmLcR20fL&sig=hSF9K6U_tY8kdE4aWHQ5_BhHTl4.*](https://books.google.com/books?hl=en&lr=&id=Ru3llwHaD_AC&oi=fnd&pg=PT3&ots=TFmLcR20fL&sig=hSF9K6U_tY8kdE4aWHQ5_BhHTl4) | Accessed 9/21/24 |  |  |
| **Breen E** | **2019** | [Breen, E., et al. “Spirituality.” Enhancing Clinical Case Formulation, //, https://doi.org/10.4324/9780429486418-7.](https://doi.org/10.4324/9780429486418-7) | Accessed 9/20/24 |  |  |
| **Briggs M** | **2017** | Briggs M, Morzinski JA, Ellis J. Influences of a church-based intervention on falls risk among seniors. Wisconsin Med J. 2017;116(3):161-164.  <https://wmjonline.org/wp-content/uploads/2017/116/3/161.pdf> | Accessed  1/11/25 |  |  |
| **Brown P** | **2021** | Brown, P. (2021). An investigation of the presence of religion and spirituality in social work education focusing on social work educators’ familiarity with spiritual assessment tools. *ProQuest Dissertations & Theses Global*. (2557837863). Thesis. Doctor of Philosophy in Social Welfare (Ph.D.) Wurzweiler School of Social Work Yeshiva University New York. <https://www.proquest.com/dissertations-theses/investigation-presence-religion-spirituality/docview/2557837863/se-2> | *Accessed 9/20/24* |  |  |
| **Brown JL** | **2019** | Brown JL. The experiences of seven women living with pelvic surgical mesh complications. *Int Urogynecol J.* 2020; **31**:823–829. <https://doi.org/10.1007/s00192-019-04155-w> | Accessed 9/20/24 |  |  |
| **Bull AW** | **2012** | [Bull, A. W. The Insights Gained from a Portfolio of Spiritual Assessment Tools Used with Hospitalised School-Aged Children to Facilitate the Delivery of Spiritual Care Offered by the …. Query date: 2023-06-28 16:19:16. theses.gla.ac.uk, 2013. https://theses.gla.ac.uk/4529/1/2013bullphd.pdf.](https://theses.gla.ac.uk/4529/1/2013bullphd.pdf) | Accessed 9/21/24 |  |  |
| **Bush RS** | **2022** | [Bush, RS, et al. “Building Spiritual Care Competency in Undergraduate Psychiatric Mental Health Nursing Students: A Quality Improvement Project.” JOURNAL OF HOLISTIC NURSING, May 2022, https://doi.org/10.1177/08980101221103104. WOS:000800561300001.](https://doi.org/10.1177/08980101221103104) | Accessed 9/20/24 |  |  |
| **Büssing A** | **2021** | [Büssing, A. “Application and Implementation of the Spiritual Needs Questionnaire in Spiritual Care Processes.” Spiritual Needs in Research and Practice: The Spiritual …, //, https://doi.org/10.1007/978-3-030-70139-0_6.](https://doi.org/10.1007/978-3-030-70139-0_6) | Accessed 9/20/24 |  |  |
| **Cadge W** | **2015** | [Cadge, W, and J Bandini. “The Evolution of Spiritual Assessment Tools in Healthcare.” SOCIETY 52, no. 5 (October 2015): 430–37. https://doi.org/10.1007/s12115-015-9926-y.](https://doi.org/10.1007/s12115-015-9926-y) | Accessed 9/20/24 |  |  |
| **Campbell D** | **2023** | Campbell D, Robison J (Gigi), Godsey JA. Standardized Spiritual Screening Increases Chaplain Referrals Through the EMR: A Nurse-Chaplain Collaboration for Holistic Acute Healthcare. *Journal of Holistic Nursing*. 2023;41(1):30-39. doi:10.1177/08980101221079463  <https://doi.org/10.1177/08980101221079463>. | Accessed  1/11/25 |  |  |
| **Canteros MF** | **2021** | CANTEROS, Marcelo Fonseca. Attention to the spiritual and religious needs of patients by healthcare personnel. A model based on spiritual accompaniment.. Medical Research Archives, [S.l.], v. 9, n. 12, dec. 2021. ISSN 2375-1924. Available at: <https://esmed.org/MRA/mra/article/view/2633>. Date accessed: 02 feb. 2024. doi: <https://doi.org/10.18103/mra.v9i12.2633>. | Accessed 9/21/24 |  |  |
| **Canteros MF** | **2016** | Canteros MF 2016. Importancia de los aspectos espirituales y religiosos en la atención de pacientes quirúrgicos. Importance of spiritual and religious aspects in treatment of surgical patients. *Revista Chilena de Cirugía*. 2016;68(3):258-264. <https://doi.org/10.1016/j.rchic.2016.03.011>. | Accessed 9/7/24 | Spanish | **Yes**  **Full Version**  **Spanish** |
| **Carpenter JG** | **2012** | [Carpenter, JG, and PH Berry. “Refractory Cancer Pain in a Nursing Home Resident.” JOURNAL OF HOSPICE & PALLIATIVE NURSING, vol. 14, no. 8, Dec. 2012, pp. 516–21, https://doi.org/10.1097/NJH.0b013e318273b983. WOS:000310913700004.](https://doi.org/10.1097/NJH.0b013e318273b983) | Accessed 9/20/24 |  |  |
| **Carter EW** | **2019** | [Carter, E. W. “Spirituality and the Family Unit.” Systemically Treating Autism, //. https://doi.org/10.4324/9781315141831-22. Carter, E. W. “21 Spirituality and the Family Unit.” Systemically Treating Autism: A Clinician’s Guide for …, //, https://books.google.com/books?hl=en&lr=&id=0ueIDwAAQBAJ&oi=fnd&pg=PT320&ots=MM8M5VjSjD&sig=j3J-bSa2NG09iToZq6_wnSyF6P8.](https://doi.org/10.4324/9781315141831-22) | Accessed 9/20/24 |  |  |
| **Chidarikire S** | **2012** | [Chidarikire, S. “Spirituality: The Neglected Dimension of Holistic Mental Health Care.” Advances in Mental Health, //, https://doi.org/10.5172/jamh.2012.10.3.298.](https://doi.org/10.5172/jamh.2012.10.3.298) | Accessed 9/20/24 |  |  |
| **Chrash M** | **2011** | [Chrash, M., et al. “The APN Role in Holistic Assessment and Integration of Spiritual Assessment for Advance Care Planning.” Journal of the American …, //, https://doi.org/10.1111/j.1745-7599.2011.00644.x.](https://doi.org/10.1111/j.1745-7599.2011.00644.x) | Accessed 9/20/24 |  |  |
| **Christian O** | **2018** | [Christian, O. “TOWARD AN UNDERSTANDING OF SPIRITUALITY.” Psychiatric Nursing-EBook, //. https://books.google.com/books?hl=en&lr=&id=umRgDwAAQBAJ&oi=fnd&pg=PA57&ots=fM6w3mz-Rj&sig=6GP_2Q86gCHYoFaTZh8F5bLrUfg.](https://books.google.com/books?hl=en&lr=&id=umRgDwAAQBAJ&oi=fnd&pg=PA57&ots=fM6w3mz-Rj&sig=6GP_2Q86gCHYoFaTZh8F5bLrUfg) | Accessed 9/20/24 |  |  |
| **Cist A** | **2017** | [Choi, A. C. P. “Religion and Spirituality in the Intensive Care Unit.” Spirituality and Religion Within the Culture of …, //, https://books.google.com/books?hl=en&lr=&id=kjskDwAAQBAJ&oi=fnd&pg=PA165&ots=xLOSZKHeuh&sig=abhKKPPy-c7a7PrSvxlBBJ4XgnE.](https://books.google.com/books?hl=en&lr=&id=kjskDwAAQBAJ&oi=fnd&pg=PA165&ots=xLOSZKHeuh&sig=abhKKPPy-c7a7PrSvxlBBJ4XgnE) | Accessed 9/20/24 |  |  |
| **Clark WS** | **2008** | [Clark, W. S. Nurses’ Attitudes and Barriers toward Spirituality When Caring for Terminally Ill Patients. search.proquest.com, 2008, https://search.proquest.com/openview/a538d68a277725761523900b9bad2788/1?pq-origsite=gscholar&cbl=18750.](https://search.proquest.com/openview/a538d68a277725761523900b9bad2788/1?pq-origsite=gscholar&cbl=18750) | Accessed 9/20/24 |  |  |
| **Clement D** | **2016** | [Clement, D., and D. LaGuerre. “Role of Religion and Spirituality in Sport Injury Rehabilitation.” Sport, Psychology and …, //, https://doi.org/10.4324/9781351165488-6.](https://doi.org/10.4324/9781351165488-6) | Accessed 9/20/24 |  |  |
| **Cook CCH** | **2015** | [Cook CCH. Religion and spirituality in clinical practice. BJPsych Advances. 2015;21(1):42-50. doi:10.1192/apt.bp.114.013276](https://doi.org/10.1192/apt.bp.114.013276) | Accessed 9/20/24 |  |  |
| **Cooper Z** | **2022** | [Cooper, Z. “Spirituality in Primary Care Settings: Addressing the Whole Person through Christian Mindfulness.” RELIGIONS 13, no. 4 (April 2022). https://doi.org/10.3390/rel13040346.](https://doi.org/10.3390/rel13040346) | Accessed  9/20/24 |  |  |
| **Culliford L** | **2017** | [Culliford, L. “Taking a Spiritual History.” Advances in Psychiatric Treatment, //. https://www.cambridge.org/core/journals/advances-in-psychiatric-treatment/article/taking-a-spiritual-history/6005C8AEE855867192AA66935332C428 https://www.cambridge.org/core/services/aop-cambridge-core/content/view/6005C8AEE855867192AA66935332C428/S1355514600003850a.pdf/taking_a_spiritual_history.pdf.](https://www.cambridge.org/core/journals/advances-in-psychiatric-treatment/article/taking-a-spiritual-history/6005C8AEE855867192AA66935332C428?utm_campaign=shareaholic&utm_medium=copy_link&utm_source=bookmark) | Accessed 9/20/24 |  |  |
| **de Queiroz CM** | **2022** | de Queiroz , C. M. ., Abdalla , I. R. ., Aragão , R. D. D. ., & Hermita , R. P. de M. . (2022). Anamnese espiritual e relação médico-paciente: revisão e reconstrução de um instrumento cotidiano: Spiritual anamnesis and doctor-patient relationship: review and reconstruction of a daily routine instrument. *STUDIES IN HEALTH SCIENCES*, *3*(2), 1128–1141. <https://doi.org/10.54022/shsv3n2-039>  <https://ojs.studiespublicacoes.com.br/ojs/index.php/shs/article/view/560/568> | Accessed 9/21/24 | Portuguese | **Yes**  **Full Version**  **Portuguese** |
| **Dehaven MJ** | **2001** | [*DeHaven, MJ. “Comments on Spiritual Assessment and Medicine.” AMERICAN FAMILY PHYSICIAN, vol. 64, no. 3, Aug. 2001, pp. 373–74. WOS:000170370600003.*](https://pubmed.ncbi.nlm.nih.gov/11515827/)  https://pubmed.ncbi.nlm.nih.gov/11515827/ | Accessed 9/21/24 |  |  |
| **Delbridge E** | **2014** | [Delbridge, E., et al. “Honoring the ‘Spiritual’ in Biopsychosocial-Spiritual Health Care: Medical Family Therapists on the Front Lines of Graduate Education, Clinical Practice, and Research.” Medical Family Therapy: Advanced …, //, https://doi.org/10.1007/978-3-319-03482-9_11.](https://doi.org/10.1007/978-3-319-03482-9_11) | Accessed 9/20/24 |  |  |
| **Demirsoy N** | **2017** | [Demirsoy, N. “Holistic Care Philosophy for Patient‐centered Approaches and Spirituality.” Patient Centered Medicine, //, https://books.google.com/books?hl=en&lr=&id=sviODwAAQBAJ&oi=fnd&pg=PA119&ots=B0dF9AzDKL&sig=R3NT8LLxfjcMs3Y8y2Fl3i0riBE https://www.intechopen.com/chapters/54224.](https://books.google.com/books?hl=en&lr=&id=sviODwAAQBAJ&oi=fnd&pg=PA119&ots=B0dF9AzDKL&sig=R3NT8LLxfjcMs3Y8y2Fl3i0riBE%20https://www.intechopen.com/chapters/54224) | Accessed 9/20/24 |  |  |
| **Deodhar J** | **2022** | [Deodhar, J., C. Park, and M. Lazenby. “Spiritually Sensitive Care in Palliative and End of Life Settings.” … Oncology in Palliative and End of …, //. https://books.google.com/books?hl=en&lr=&id=172ZEAAAQBAJ&oi=fnd&pg=PT187&ots=e7PknhiAx4&sig=lcVYLJEOLU9bbBBpnaAKS4B29Dc.](https://books.google.com/books?hl=en&lr=&id=172ZEAAAQBAJ&oi=fnd&pg=PT187&ots=e7PknhiAx4&sig=lcVYLJEOLU9bbBBpnaAKS4B29Dc) | Accessed 9/20/24 |  |  |
| **Desmond ME** | **2017** | [Desmond, M. E. Simulation Using a Standardized Patient to Measure Perception and Congruency of Spiritual Care for a Veteran from Three Perspectives to Inform Nursing …. //, https://search.proquest.com/openview/d7685704535be005f51ba6c6337c7544/1?pq-origsite=gscholar&cbl=18750&casa_token=3I8IuqHeQ78AAAAA:wBNIOF23OshA7Ottqtg4MD8R7P2K7tV1I0yJsw-ez34U6AaRo6iFTtoozofiKdmpO7jB0zQCmg.](https://search.proquest.com/openview/d7685704535be005f51ba6c6337c7544/1?pq-origsite=gscholar&cbl=18750&casa_token=3I8IuqHeQ78AAAAA:wBNIOF23OshA7Ottqtg4MD8R7P2K7tV1I0yJsw-ez34U6AaRo6iFTtoozofiKdmpO7jB0zQCmg) | Accessed 9/20/24 |  |  |
| **Dhar N** | **2012** | [Dhar, Neera, et al. “Self Evolution: 1(St) Domain of Spiritual Health.” Ayu, vol. 33, no. 2, Apr. 2012, pp. 174–77, https://doi.org/10.4103/0974-8520.105234.](https://doi.org/10.4103/0974-8520.105234) | Accessed 9/20/24 |  |  |
| **Dhar N** | **2013** | [Dhar, N., S. K. Chaturvedi, and D. Nandan. “Spiritual Health, the Fourth Dimension: A Public Health Perspective.” WHO South-East Asia Journal of …, //. https://apps.who.int/iris/bitstream/handle/10665/329763/seajphv2n1_p3.pdf.](https://apps.who.int/iris/bitstream/handle/10665/329763/seajphv2n1_p3.pdf) | Accessed 9/20/24 |  |  |
| **Dhar N** | **2011** | [Dhar, N., S. K. Chaturvedi, and D. Nandan. “Spiritual Health Scale 2011: Defining and Measuring 4th Dimension of Health.” Indian Journal of Community …, //. https://www.ncbi.nlm.nih.gov/pmc/articles/PMC3263147/.](https://www.ncbi.nlm.nih.gov/pmc/articles/PMC3263147/) | Accessed 9/20/24 |  |  |
| **Di Placido M** | **2023** | Di Placido M, Palmisano S, Timmins F. (2023). Institutional, Nurses’ and Patients’ Spiritual Imaginaries Compared: Sociological Notes from the Field. *Fieldwork in Religion*. 2023;18(2):158–183. <https://doi.org/10.1558/firn.25878> | Accessed 9/21/24 |  | Translated to Italian for their study;  Translation not in article |
| **Dillard V** | **2021** | Dillard, V., et al. “Attitudes, Beliefs and Behaviors of Religiosity, Spirituality, and Cultural Competence in the Medical Profession: A Cross-Sectional Survey Study.” PLOS ONE, vol. 16, no. 6, June 2021, https://doi.org/10.1371/journal.pone.0252750. WOS:000665475100010. | Accessed  1/11/25 |  |  |
| **Doreen AW** | **2016** | *Doreen, A. W. Spirituality in Nursing Practice: The Basics and Beyond. Query date: 2023-06-28 16:19:16. books.google.com, 2016.* [*https://books.google.com/books?hl=en&lr=&id=VoJKDAAAQBAJ&oi=fnd&pg=PP1&ots=Rwiil5X_6X&sig=qVQ4jkyk3cge5sN1rtpYz3uWfoQ.*](https://books.google.com/books?hl=en&lr=&id=VoJKDAAAQBAJ&oi=fnd&pg=PP1&ots=Rwiil5X_6X&sig=qVQ4jkyk3cge5sN1rtpYz3uWfoQ) | Accessed 9/20/24 |  |  |
| **Drury C** | **2016** | [Drury, C., and J. Hunter. “The Hole in Holistic Patient Care.” Open Journal of Nursing, //. https://www.scirp.org/html/12-1440693_70974.htm.](https://www.scirp.org/html/12-1440693_70974.htm) | Accessed 9/20/24 |  |  |
| **Dudgeon D** | **2022** | [Dudgeon, D., and L. M. Herx. “Response to Suffering.” Palliative Medicine: A Case …, //, https://books.google.com/books?hl=en&lr=&id=LYpNEAAAQBAJ&oi=fnd&pg=PA381&ots=Slqs9WhER6&sig=2JVMOXodRSpoIYqKSIjZ5XQB93c.](https://books.google.com/books?hl=en&lr=&id=LYpNEAAAQBAJ&oi=fnd&pg=PA381&ots=Slqs9WhER6&sig=2JVMOXodRSpoIYqKSIjZ5XQB93c) | Accessed 9/20/24 |  |  |
| **Dugan BDA** | **2011** | Dugan, BDA., et al. “Integrating Spirituality in Patient Care: Preparing Students for the Challenges Ahead.” *Currents in Pharmacy Teaching and Learning*. 2011; 3(4):260-266.  <https://www.sciencedirect.com/science/article/pii/S1877129711000670>. | Accessed 1/11/25 |  |  |
| **Dugdale LS** | **2017** | [Dugdale, L. S. “RELIGION AND SPIRITUALITY ININTERNAL MEDICINE.” Spirituality and Religion Within the Culture of …, //, https://books.google.com/books?hl=en&lr=&id=kjskDwAAQBAJ&oi=fnd&pg=PA79&ots=xLOSZKIdwi&sig=L1auO8YahHtoLr6jJibpiX1fsoA.](https://books.google.com/books?hl=en&lr=&id=kjskDwAAQBAJ&oi=fnd&pg=PA79&ots=xLOSZKIdwi&sig=L1auO8YahHtoLr6jJibpiX1fsoA) | Accessed 9/20/24 |  |  |
| **Duke N** | **2016** | [Duke, N., and W. Wigley. “Literature Review: The Self-Management of Diet, Exercise and Medicine Adherence of People with Type 2 Diabetes Is Influenced by Their Spiritual Beliefs.” Journal of Diabetes Nursing, //, http://repository.uwl.ac.uk/id/eprint/3460/ http://repository.uwl.ac.uk/id/eprint/3460/1/Duke-Wigley-2016-Literature-review-the-self-management-of-diet-exercise-and-medicine.pdf.](http://repository.uwl.ac.uk/id/eprint/3460/1/Duke-Wigley-2016-Literature-review-the-self-management-of-diet-exercise-and-medicine.pdf) | Accessed 9/21/24 |  |  |
| **Dumont MB** | **2019** | [Dumont, M. B., and A. Solis Suarez. La Spiritualité En Soins Palliatifs Pédiatriques. //, https://sonar.ch/documents/315612/files/TDB_Dumont_Solis.pdf.](https://sonar.ch/documents/315612/files/TDB_Dumont_Solis.pdf) | Accessed 9/7/24 | French | No |
| **Ebertsch C** | **2019** | [Ebertsch, C. Does Performing a Spiritual Assessment on a Patient Enhance Patient Outcomes: An Integrative Literature Review. //, https://via.library.depaul.edu/nursing-colloquium/2018/winter/18/.](https://via.library.depaul.edu/nursing-colloquium/2018/winter/18/) | Accessed 9/20/24 |  |  |
| **Edwards W** | **2007** | [Edwards, W., and P. Gilbert. “Spiritual Assessment: Narratives and Responses.” Spirituality, Values and Mental Health, //, https://books.google.com/books?hl=en&lr=&id=aPQPBQAAQBAJ&oi=fnd&pg=PA144&ots=2djYr0zC5s&sig=QgsnR_EG8CfEDwAEzVTM8siOuyM.](https://books.google.com/books?hl=en&lr=&id=aPQPBQAAQBAJ&oi=fnd&pg=PA144&ots=2djYr0zC5s&sig=QgsnR_EG8CfEDwAEzVTM8siOuyM) | Accessed 9/20/24 |  |  |
| **Elliot N** | **2017** | Elliott N. Faith, ethics and social work: Framework for an Introductory Lecture^*^. *Ethics and Social Welfare*. 2017; 11(1): 92–99. <https://doi.org/10.1080/17496535.2017.1287633>. | Accessed 1/11/25 |  |  |
| **Eriksson-Tapio L** | **2017** | Eriksson-Tapio, L. TOIVOA JA VOIMAA HENGELLISYYDESTÄ:-Hoidossa Olevan Ihmisen Toiveita Ja Kokemuksia.  https://www.theseus.fi/handle/10024/138407 | Accessed 9/7/24 | Finnish | Brief translation  Finnish |
| **Esperandio M** | **2020** | Esperandio M, Leget C.  [Leget, C., and M. Esperandio. “Espiritualidade Nos Cuidados Paliativos: Questão de Saúde Pública?” Revista Bioética, //, https://www.redalyc.org/journal/3615/361570652020/361570652020.pdf.](https://www.redalyc.org/journal/3615/361570652020/361570652020.pdf) | Accessed 9/7/24 | Portuguese | Brief translation  Portuguese |
| **Estupiñan B** | **2017** | Estupiñan, B., Kibble, J. The Relationship Between Spirituality and Burnout in the Lives of Medical Students. *Med.Sci.Educ.* **28**, 37–44 (2018). <https://doi.org/10.1007/s40670-017-0490-5> | Accessed 1/11/25 |  |  |
| **Eyres P** | **2019** | [Eyres, P. An Exploration of Religious Practice as a Valued Occupation. pearl.plymouth.ac.uk, 2020, https://pearl.plymouth.ac.uk/handle/10026.1/16755](https://pearl.plymouth.ac.uk/handle/10026.1/16755%20https:/pearl.plymouth.ac.uk/bitstream/handle/10026.1/16755/2020EYRES10272485PhD.pdf?sequence=1) ~~https://pearl.plymouth.ac.uk/bitstream/handle/10026.1/16755/2020EYRES10272485PhD.pdf?sequence=1.~~  <https://dx.doi.org/10.24382/413> | Accessed 9/21/24 |  |  |
| **Fanning JB** | **2008** | [Fanning, J. B. Genetic Counseling and the Spirit of Communication. ir.vanderbilt.edu, 2008, https://ir.vanderbilt.edu/handle/1803/11685 https://ir.vanderbilt.edu/bitstream/handle/1803/11685/Fanningfinal.pdf?sequence=1&isAllowed=y.](https://ir.vanderbilt.edu/bitstream/handle/1803/11685/Fanningfinal.pdf?sequence=1&isAllowed=y) | Accessed 9/21/24 |  |  |
| **Feldman DB** | **2022** | [Feldman, DB, et al. “Healthcare Professionals’ Lay Definitions of Hope.” JOURNAL OF HAPPINESS STUDIES, vol. 24, no. 1, Jan. 2023, pp. 231–47, https://doi.org/10.1007/s10902-022-00589-0. WOS:000879140700003.](https://doi.org/10.1007/s10902-022-00589-0) | Accessed 9/20/24 |  |  |
| **Feldstein BD** | **2008** | Feldstein, C. B. D., Grudzen, M., Johnson, A., & LeBaron, S. (2008). Integrating Spirituality and Culture with End-of-Life Care in Medical Education. *Clinical Gerontologist*, *31*(4), 71–82. <https://doi.org/10.1080/07317110801947185> | Accessed 9/20/24 |  |  |
| **Felgoise SH** | **2010** | Felgoise, S. H., Becker, M. A., & Jebitsch, J. L. (2010). Spirituality. In R. A. DiTomasso, B. A. Golden, & H. Morris (Eds.), Handbook of cognitive behavioral approaches in primary care (pp. 129–156). Springer Publishing Company. | Book |  |  |
| **Fick JL** | **2006** | Fick, Jennifer. *Patient Perspectives on Discussing Spirituality in Genetics Clinic: A Cross-sectional Analysis of Relevance and Comfort.* 2006. University of Cincinnati, Master's thesis. *OhioLINK Electronic Theses and Dissertations Center*, <http://rave.ohiolink.edu/etdc/view?acc_num=ucin1148316686>. | Accessed 9/21/24 |  |  |
| **Finocchiaro DN** | **2017** | [Finocchiaro, D. N. “Supporting the Patient’s Spiritual Needs at the End of Life.” Nursing2022https://journals.lww.com/nursing/Fulltext/2016/05000/Supporting_the_patient_s_spiritual_needs_at_the.16.aspx.](https://journals.lww.com/nursing/Fulltext/2016/05000/Supporting_the_patient_s_spiritual_needs_at_the.16.aspx) | Accessed 9/20/24 |  |  |
| **Flannagan T** | **2020** | [Flanagan, T. Narrative Medicine in Hospice Care: Identity, Practice, and Ethics through the Lens of Paul Ricoeur. books.google.com, 2019, https://books.google.com/books?hl=en&lr=&id=JkHCDwAAQBAJ&oi=fnd&pg=PP1&ots=JkiqNhffDH&sig=YEQNG-N9JxadlLs1SVhwk4XB8o8.](https://books.google.com/books?hl=en&lr=&id=JkHCDwAAQBAJ&oi=fnd&pg=PP1&ots=JkiqNhffDH&sig=YEQNG-N9JxadlLs1SVhwk4XB8o8) | Accessed 9/20/24 |  |  |
| **Fleming NL** | **2020** | [Fleming, N. L. Interreligious Pastoral Counselling in the Context of a Seventh-Day Adventist Psychiatric Clinic. //, https://repository.up.ac.za/bitstream/handle/2263/87861/Fleming_Interreligious_2020.pdf?sequence=1.](https://repository.up.ac.za/bitstream/handle/2263/87861/Fleming_Interreligious_2020.pdf?sequence=1) | Accessed 9/20/24 |  |  |
| **Fopka-Kowalczyk M** | **2022** | Fopka-Kowalczyk M, Machul M, Dobrowolska B. Research protocol of the Polish adaption and validation of HOPE scale: Qualitative measurement of patients’ spiritual needs. J Pall Med. 2022;25(10):1492-1500. <https://doi.org/10.1089/jpm.2021.0530> | Accessed 9/20/24 | Polish | **Yes**  **Full Version**  **Polish** |
| **Fopka-Kowalczyk M** | **2020** | [Fopka-Kowalczyk, M. “Providing Support and Spiritual Care to People with Chronic Diseases.” Paedagogia Christiana, //, https://apcz.umk.pl/PCh/article/view/Pch.2020.023 https://apcz.umk.pl/PCh/article/download/Pch.2020.023/27890.](https://apcz.umk.pl/PCh/article/view/Pch.2020.023%20https:/apcz.umk.pl/PCh/article/download/Pch.2020.023/27890) | Accessed 9/21/24 | Polish | No |
| **Fox J** | **2019** | [Fox, J., and P. Videmšek. “ISTRAŽIVANJE MENTALNOG ZDRAVLJA I DUHOVNOG OPORAVKA STRUČNJAKA PO ISKUSTVU: RASPRAVA O JEDINSTVENOM DOPRINOSU SOCIJALNIH ….”](https://hrcak.srce.hr/clanak/330514%20https:/hrcak.srce.hr/file/330514)  [**https://hrcak.srce.hr/226963**](https://hrcak.srce.hr/226963) | Accessed 9/7/24 | Croatian | No |
| **Garbutt D** | **2014** | *Garbutt, D. “The Principles of Death, Dying and Bereavement.” Nursing Practice: Knowledge and Care, //.* [*https://books.google.com/books?hl=en&lr=&id=P1pEBAAAQBAJ&oi=fnd&pg=PA409&ots=BiUDFBMDyi&sig=fJUBBbGQ9TNi7zIDZ89Y83kIWsc.*](https://books.google.com/books?hl=en&lr=&id=P1pEBAAAQBAJ&oi=fnd&pg=PA409&ots=BiUDFBMDyi&sig=fJUBBbGQ9TNi7zIDZ89Y83kIWsc) | Accessed 9/20/24 |  |  |
| **Gauznabi S** | **2022** | *Gauznabi, S. “Managing the Hope for a Miracle: A Reflection.” JOURNAL OF PRIMARY HEALTH CARE, vol. 14, no. 3, 2022, pp. 280–82, ~~https://doi.org/10.1071/HC22070.~~ WOS:000852722600001.*  [*https://researchspace.auckland.ac.nz/bitstream/handle/2292/65580/Published%20Article.pdf?sequence=1&isAllowed=y*](https://researchspace.auckland.ac.nz/bitstream/handle/2292/65580/Published%20Article.pdf?sequence=1&isAllowed=y) | Accessed 9/21/24 |  |  |
| **Gellerman D** | **2016** | [Gellerman, D., D. Hinton, and F. G. Lu. Supplementary Model 5: Spirituality, Religion and Moral Traditions. Query date: 2023-06-28 16:19:16. books.google.com, 2016. https://books.google.com/books?hl=en&lr=&id=p6gvCgAAQBAJ&oi=fnd&pg=PA90&ots=IytETrbQJ6&sig=Bsqka_LRydegSzCKVkpyHDFU9eE.](https://books.google.com/books?hl=en&lr=&id=p6gvCgAAQBAJ&oi=fnd&pg=PA90&ots=IytETrbQJ6&sig=Bsqka_LRydegSzCKVkpyHDFU9eE) | Accessed 9/20/24 |  |  |
| **Gibeau C** | **2009** | [Gibeau, C. Perception and Quality of Life: Contrasting Personal Faith Based Wellness and the Traditional Medical Models of Care Used in Alcohol Abuse and Addiction Treatment …. scholarworks.smith.edu, 2009, https://scholarworks.smith.edu/theses/1195/ https://scholarworks.smith.edu/cgi/viewcontent.cgi?article=2272&context=theses.](https://scholarworks.smith.edu/cgi/viewcontent.cgi?article=2272&context=theses) | Accessed 9/21/24 |  |  |
| **Giorgio B** | **2005** | [Georgio B. “Valuing” education for future consciousness: The role of connectedness and hope. An empirical study of an Australian cohort of first year psychology students. Paper presented at 2nd Asia-Pacific Educational Integrity Conference: Educational Integrity: Values in Teaching, Learning & Research Newcastle 2-3 December 2005.](http://brahmakumaris.info/download/BK%20Academic%20papers/Giorgio-latest.pdf)  [http://brahmakumaris.info/download/BK%20Academic%20papers/Giorgio-latest.pdf.](http://brahmakumaris.info/download/BK%20Academic%20papers/Giorgio-latest.pdf) | Accessed 9/21/24 |  |  |
| **Goldberg MJ** | **2015** | [Goldberg, M. J. How Spirituality Training Impacts the Practice of Social Workers Assisting Elderly Clients in End-of-Live Care: A Hermeneutical Phenomenological Approach. ourspace.uregina.ca, 2015, https://ourspace.uregina.ca/handle/10294/5856 https://ourspace.uregina.ca/bitstream/handle/10294/5856/Goldberg_Michael_200286029_MA_GRON_Spring2015.pdf.](https://ourspace.uregina.ca/bitstream/handle/10294/5856/Goldberg_Michael_200286029_MA_GRON_Spring2015.pdf) | Accessed 9/21/24  both links |  |  |
| **Gomi S** | **2013** | Gomi, S., et al. “Spiritual Assessment in Mental Health Recovery.” COMMUNITY MENTAL HEALTH JOURNAL, vol. 50, no. 4, May 2014, pp. 447–53,  *doi: 10.1007/s10597-013-9653-z.*  [*https://link.springer.com/article/10.1007/s10597-013-9653-z*](https://link.springer.com/article/10.1007/s10597-013-9653-z) | Accessed 9/21/24 |  |  |
| **Gore JD** | **2013** | [Gore, J. D. Providing Holistic and Spiritual Nursing Care. digitalcommons.liberty.edu, 2013, https://digitalcommons.liberty.edu/honors/370/ https://digitalcommons.liberty.edu/cgi/viewcontent.cgi?article=1383&context=honors.](https://digitalcommons.liberty.edu/honors/370/%20https:/digitalcommons.liberty.edu/cgi/viewcontent.cgi?article=1383&context=honors) | Accessed 9/21/24 both links |  |  |
| **Gorman LM** | **2008** | [Gorman, L. M., and D. F. Sultan. “PSYCHOSOCIAL NURSING.” Mis.Kp.Ac.Rw,](https://mis.kp.ac.rw/admin/admin_panel/kp_lms/files/digital/Core%20Books/Nursing/Psychosocial%20Nursing%20for%20general%20patient%20care.%203rd%20ed.%5B%20Gorman,%20Linda%20M.%5D.pdf) ~~https://mis.kp.ac.rw/admin/admin_panel/kp_lms/files/digital/Core%20Books/Nursing/Psychosocial%20Nursing%20for%20general%20patient%20care.%203rd%20ed.%5B%20Gorman,%20Linda%20M.%5D.pdf.~~  <https://www.ifeet.org/files/Psychosocial-Nursing-for-General-Patient-Care-by-Linda-Gorman,-Donna-Sultan.pdf> | Accessed 9/21/24 |  |  |
| **Grad D** | **2016** | [D. Grad. “Spiritualität in Der Sozialen Arbeit.” Researchgate.Net, n.d. https://www.researchgate.net/profile/David-Grad-2/publication/315707192_Spiritualitat_in_der_Sozialen_Arbeit_-_Ansatze_einer_spirituell-sensitiven_Sozialen_Arbeit/links/58dd8662a6fdcc3c6abf9656/Spiritualitaet-in-der-Sozialen-Arbeit-Ansaetze-einer-spirituell-sensitiven-Sozialen-Arbeit.pdf.](https://www.researchgate.net/profile/David-Grad-2/publication/315707192_Spiritualitat_in_der_Sozialen_Arbeit_-_Ansatze_einer_spirituell-sensitiven_Sozialen_Arbeit/links/58dd8662a6fdcc3c6abf9656/Spiritualitaet-in-der-Sozialen-Arbeit-Ansaetze-einer-spirituell-sensitiven-Sozialen-Arbeit.pdf) | Accessed 9/7/24 | German | Brief translation  German |
| **Grant A** | **2007** | *Grant, A. “Spirituality, Health and the Complementary Medicine Practitioner.” JOURNAL OF THE AUSTRALIAN TRADITIONAL-MEDICINE SOCIETY, vol. 13, no. 4, Dec. 2007, pp. 207–09. WOS:000257189800002.*  [link.gale.com/apps/doc/A173677448/AONE?u=anon~e8f277d6&sid=googleScholar&xid=dd729f3f](http://link.gale.com/apps/doc/A173677448/AONE?u=anon~e8f277d6&sid=googleScholar&xid=dd729f3f) | *Accessed 9/21/24* |  |  |
| **Gupta P** | **2014** | Gupta PS, Anandarajah G. The role of spirituality in diabetes self-management in an urban, underserved population: a qualitative exploratory study. R I Med J (2013). 2014 Mar 3;97(3):31-5. PMID: 24596928. |  |  |  |
| **Haire J** | **2010** | *Haire, J., et al. Ageing and Spirituality across Faiths and Cultures. books.google.com, 2010, https://books.google.com/books?hl=en&lr=&id=goYcTM7ztqcC&oi=fnd&pg=PP1&ots=UH3Z9Flqt2&sig=1rrYL4Np0hg9bICso9tiYqPLH08.* | Accessed 9/20/24 |  |  |
| **Hamilton JL** | **2001** | [*Hamilton, JL, and GR Swian. “Comments on Spiritual Assessment and Medicine.” AMERICAN FAMILY PHYSICIAN, vol. 64, no. 3, Aug. 2001, pp. 376-+. WOS:000170370600005.*](https://pubmed.ncbi.nlm.nih.gov/11515829/) | Accessed 9/20/24 |  |  |
| **Hasimah C** | **2018** | Chick H. Punca utama keresahan spiritual dalam penjagaan paliatif menurut perspektif psikologi agama  <https://www.academia.edu/115152453/Punca_utama_keresahan_spiritual_dalam_penjagaan_paliatif_menurut_perspektif_psikologi_agama_Hasimah_Chik?uc-sb-sw=26870131> | Accessed 9/20/24 | Malay | No |
| **Haufe M** | **2020** | [Haufe, M., et al. “How Can Existential or Spiritual Strengths Be Fostered in Palliative Care? An Interpretative Synthesis of Recent Literature.” BMJ Supportive & …, //, https://spcare.bmj.com/content/early/2020/09/13/bmjspcare-2020-002379?int_source=trendmd&int_medium=cpc&int_campaign=usage-042019.](https://spcare.bmj.com/content/early/2020/09/13/bmjspcare-2020-002379?int_source=trendmd&int_medium=cpc&int_campaign=usage-042019) | Accessed 9/20/24 |  |  |
| **Hemphill B** | **2015** | Hemphill B. Spiritual assessments in occupational therapy. *The Open J of Occup Therapy.* 2015; 3(3).  [*https://doi.org/10.15453/2168-6408.1159*](https://doi.org/10.15453/2168-6408.1159) | Accessed 9/21/24 |  |  |
| **Herschkopf M** | **2017** | Herschkopf and Jafari, “Religion and Spirituality in Medical Education.” In: Spirituality and Religion Within the Culture of Medicine: From Evidence to ... https://books.google.com/books?hl=en&lr=&id=OMudDgAAQBAJ&oi=fnd&pg=PA195&ots=iYte420sgU&sig=A607RGf-HaHHzmhX7OWFfcPLjCk | Accessed 9/20/24 |  |  |
| **Higginbotham AR** | **2002** | [Higginbotham, AR, and TR Marcy. “Spiritual Assessment: A New Outlook on the Pharmacist’s Role.” AMERICAN JOURNAL OF HEALTH-SYSTEM PHARMACY, vol. 63, no. 2, Jan. 2006, pp. 169–73, https://doi.org/10.2146/ajhp050275. WOS:000234901700011.](https://doi.org/10.2146/ajhp050275) | Accessed 9/20/24 |  |  |
| **Higgins E** | **2021** | [Higgins, E., et al. “The CASH Assessment Tool: A Window into Existential Suffering.” JOURNAL OF HEALTH CARE CHAPLAINCY, vol. 28, no. 4, Oct. 2022, pp. 482–96, https://doi.org/10.1080/08854726.2021.1922980. WOS:000652139100001.](https://doi.org/10.1080/08854726.2021.1922980) | Accessed 9/20/24 |  |  |
| **Hilmersson A** | **2021** | Hilmersson, A., and S. Levin. ETT SISTA GLAS. gupea.ub.gu.se, 2021  <https://gupea.ub.gu.se/bitstream/handle/2077/68295/gupea_2077_68295_1.pdf?sequence=1> | Accessed 1/16/25 | Swedish | Brief translation  Swedish |
| **Ho JQ** | **2018** | [Ho, JQ, et al. “Spiritual Care in the Intensive Care Unit: A Narrative Review.” JOURNAL OF INTENSIVE CARE MEDICINE, vol. 33, no. 5, May 2018, pp. 279–87, https://doi.org/10.1177/0885066617712677. WOS:000429876400001.](https://doi.org/10.1177/0885066617712677) | Accessed 9/20/24 |  |  |
| **Hodge D** | **2014** | Hodge, D. “Chaplains–How Are They Known?” Health and Social Care Chaplaincy, //, https://journal.equinoxpub.com/HSCC/article/view/15409. | Accessed  1/11/15 |  |  |
| **Holloway M** | **2010** | [Holloway, M., and B. Moss. Spirituality and Social Work. Query date: 2023-06-28 12:26:39. books.google.com, 2010. https://books.google.com/books?hl=en&lr=&id=-JtGEAAAQBAJ&oi=fnd&pg=PP1&ots=o5f0TnyPmh&sig=Pnk-j9TiA4nL5ZTWVBeisTV86RM.](https://books.google.com/books?hl=en&lr=&id=-JtGEAAAQBAJ&oi=fnd&pg=PP1&ots=o5f0TnyPmh&sig=Pnk-j9TiA4nL5ZTWVBeisTV86RM) | Accessed 9/20/24 |  |  |
| **Hookers S** | **2015** | [Hooker, S., and D. B. Bekelman. “Spiritual and Existential Issues.” End-of-Life Care in Cardiovascular Disease, //, https://doi.org/10.1007/978-1-4471-6521-7_10.](https://doi.org/10.1007/978-1-4471-6521-7_10) | Accessed 9/20/24 |  |  |
| **Howington TL** | **2023** | [Howington, T. L. Identifying Key Components for Developing a Curriculum That Enhances Collaboration Between Medical Interdisciplinary Teams and Chaplains in Upstate …. //, https://search.proquest.com/openview/426ade95a958f835d9dded74a0ef3ed8/1?pq-origsite=gscholar&cbl=18750&diss=y.](https://search.proquest.com/openview/426ade95a958f835d9dded74a0ef3ed8/1?pq-origsite=gscholar&cbl=18750&diss=y) | Accessed 9/20/24 |  |  |
| **Hughes C** | **2017** | [Hughes C. The spiritual needs of inpatient mental health service users: theoretical and practical applications. Mental Health Practice. 2017; 20(8,):21-25. doi: 10.7748/mhp.2017.e1166](https://doi.org/10.7748/mhp.2017.e1166) | Accessed 9/20/24 |  |  |
| **Huguelet P** | **2009** | [Huguelet, P., and S. Mohr. “Religion, Spirituality, and Psychiatry: Spiritual Assessment and Clinical Care.” Psychiatric Times, //, https://go.gale.com/ps/i.do?id=GALE%7CA325892648&sid=googleScholar&v=2.1&it=r&linkaccess=abs&issn=08932905&p=AONE&sw=w.](https://go.gale.com/ps/i.do?id=GALE%7CA325892648&sid=googleScholar&v=2.1&it=r&linkaccess=abs&issn=08932905&p=AONE&sw=w) | Accessed 9/20/24 |  |  |
| **Huguelet P** | **2011** | [Huguelet, P., et al. “A Randomized Trial of Spiritual Assessment of Outpatients With Schizophrenia: Patients’ and Clinicians’ Experience.” PSYCHIATRIC SERVICES, vol. 62, no. 1, Jan. 2011, pp. 79–86, https://doi.org/10.1176/appi.ps.62.1.79. WOS:000285917200015.](https://doi.org/10.1176/appi.ps.62.1.79) | Accessed 9/20/24 |  |  |
| **Hunt J** | **2022** | Hunt J. Bio-psycho-social-spiritual assessment? teaching the skill of spiritual assessment.*Social Work and Christianity.* 2014; 41(4): 373-384. <https://www.proquest.com/scholarly-journals/bio-psycho-social-spiritual-assessment-teaching/docview/1655810190/se-2> | Accessed 1/11/25 |  |  |
| **Hvdit EA** | **2017** | Hvidt, EA, et al. Development of the EMAP Tool Facilitating Existential Communication between General Practitioners and Cancer Patients. EUROPEAN JOURNAL OF GENERAL PRACTICE, vol. 23, no. 1, Aug. 2017, pp. 261–68. <https://pmc.ncbi.nlm.nih.gov/articles/PMC8816390/> | Accessed 1/11/25 |  |  |
| **Hyun I** | **2013** | [Hyun, I. “Therapeutic Hope, Spiritual Distress, and the Problem of Stem Cell Tourism.” CELL STEM CELL, vol. 12, no. 5, May 2013, pp. 505–07, https://doi.org/10.1016/j.stem.2013.04.010. WOS:000329569600005.](https://doi.org/10.1016/j.stem.2013.04.010) | Accessed 9/21/24 |  |  |
| **Igboin BO** | **2017** | [Igboin, B. O. “Spirituality and Medical Practice: A Christian Perspective.” Indian Journal of Medical Ethics, //. https://www.researchgate.net/profile/Benson-Igboin/publication/315900180_Spirituality_and_medical_practice_a_Christian_perspective/links/596ce40aa6fdcc44f7d2f175/Spirituality-and-medical-practice-a-Christian-perspective.pdf.](https://www.researchgate.net/profile/Benson-Igboin/publication/315900180_Spirituality_and_medical_practice_a_Christian_perspective/links/596ce40aa6fdcc44f7d2f175/Spirituality-and-medical-practice-a-Christian-perspective.pdf) | Accessed 9/20/24 |  |  |
| **Inbadas H** | **2018** | [Inbadas, H. “Spirituality, Spiritual Care and the Role of Nurses in Palliative Care.” Palliative Care Nursing: Principles and Evidence for …, //. https://books.google.com/books?hl=en&lr=&id=GMkvEAAAQBAJ&oi=fnd&pg=PA170&ots=-rWcIZqgC6&sig=gRz4odOirIY985hv3awqfNFgXus.](https://books.google.com/books?hl=en&lr=&id=GMkvEAAAQBAJ&oi=fnd&pg=PA170&ots=-rWcIZqgC6&sig=gRz4odOirIY985hv3awqfNFgXus) | Accessed 9/20/24 |  |  |
| **Iranmanesh S** | **2011** | Iranmanesh, S., et al. “Developing and Testing a Spiritual Care Questionnaire in the Iranian Context.” JOURNAL OF RELIGION & HEALTH, vol. 51, no. 4, Dec. 2012, pp. 1104–16, https://doi.org/10.1007/s10943-011-9458-8. WOS:000311313500008. | Accessed 1/11/25 |  |  |
| **Jamil A** | **2022** | Jamil A, Jonkman LJ, Miller M, Jennings L, Connor SE. Medication adherence and health beliefs among South Asian immigrants with diabetes in the United States: A qualitative study. *J Am Coll Clin Pharm*. 2022; 5(8): 829-836. doi:[10.1002/jac5.1668](https://doi.org/10.1002/jac5.1668)  [**https://doi.org/10.1002/jac5.1668**](https://doi.org/10.1002/jac5.1668) | Accessed 9/20/24 |  | Reports translation in Hindi & Urudu, but translation not in article |
| **Jobin G** | **2020** | [Jobin, G. Spirituality in the Biomedical World: Moving between Order and “Subversion.” Query date: 2023-06-28 16:19:16. books.google.com, 2020. https://books.google.com/books?hl=en&lr=&id=cEj2DwAAQBAJ&oi=fnd&pg=PP5&ots=ACXX7-iRmE&sig=4HWcA1Ow9F6CWI2iFqwhzasSvo4.](https://books.google.com/books?hl=en&lr=&id=cEj2DwAAQBAJ&oi=fnd&pg=PP5&ots=ACXX7-iRmE&sig=4HWcA1Ow9F6CWI2iFqwhzasSvo4) | Accessed 9/20/24 |  |  |
| **Johnson CV** | **2008** | *Johnson, CV, and HL Friedman. “Enlightened or Delusional? Differentiating Religious, Spiritual, and Transpersonal Experiences from Psychopathology.” JOURNAL OF HUMANISTIC PSYCHOLOGY, vol. 48, no. 4, Oct. 2008, pp. 505–27, https://doi.org/10.1177/0022167808314174. WOS:000259639300014.* | Accessed 9/21/24 |  |  |
| **Jones BM** | **2014** | [Jones, B. M. Obesity in African American Females: A Review of Prevalence, Correlates, and Treatment Strategies with Clinical Applications. //, https://search.proquest.com/openview/7ad701bf7f81109f9339438e31c467a3/1?pq-origsite=gscholar&cbl=18750&casa_token=WMWbTNe9DlcAAAAA:in76vfKZoR377tgExWHESO2D3cTClmk9Ob5Pmp4GJajXleo2AZMAJ25gwei_QMEaj01Ijczt2A.](https://search.proquest.com/openview/7ad701bf7f81109f9339438e31c467a3/1?pq-origsite=gscholar&cbl=18750&casa_token=WMWbTNe9DlcAAAAA:in76vfKZoR377tgExWHESO2D3cTClmk9Ob5Pmp4GJajXleo2AZMAJ25gwei_QMEaj01Ijczt2A) | Accessed 9/20/24 |  |  |
| **Jones I** | **2009** | [Jones, İ, and L. L. C. Bartlett Learning. “System Foundations.” Samples.Jbpub.Com, n.d. http://samples.jbpub.com/9781284124491/Chapter2.pdf.](http://samples.jbpub.com/9781284124491/Chapter2.pdf) | Accessed 9/20/24 |  |  |
| **Jones JEH** | **2018** | Jones JEH. Spirituality and the adult survivor of parental suicide: A qualitative investigation. 2018. (Order No. 10751782). Available from ProQuest Central; ProQuest *Dissertations & Theses Global*. (2029861370). <https://www.proquest.com/dissertations-theses/spirituality-adult-survivor-parental-suicide/docview/2029861370/se-2>. | Accessed 9/20/24 |  |  |
| **Jones KF** | **2020** | Jones, K. F., Pryor, J., Care-Unger, C., & Simpson, G. (2020). “Spirituality is everybody’s business”: an exploration of the impact of spiritual care training upon the perceptions and practice of rehabilitation professionals. *Disability and Rehabilitation*, *44*(8), 1409–1418.  <https://doi.org/10.1080/09638288.2020.1820586> | Accessed 1/11/25 |  |  |
| **Jones KF** | **2021** | [Jones KF, Washington J, Kearney M, Kissane D, Best MC. Australian perspectives on spiritual care training in healthcare: A Delphi study. *Palliative and Supportive Care*. 2021;19(6):686-693. doi:10.1017/S1478951521001024 https://doi.org/10.1017/S1478951521001024. WOS:000737142900008.](%20Jones%20KF,%20Washington%20J,%20Kearney%20M,%20Kissane%20D,%20Best%20MC.%20Australian%20perspectives%20on%20spiritual%20care%20training%20in%20healthcare:%20A%20Delphi%20study. Palliative%20and%20Supportive%20Care.%202021;19(6):686-693.%20doi:10.1017/S1478951521001024%20https://doi.org/10.1017/S1478951521001024.%20WOS:000737142900008.) | Accessed 9/20/24 |  |  |
| **Jones S** | **2018** | Jones S, Sutton K, Isaacs A. Concepts, practices and advantages of spirituality among people with a chronic mental illness in Melbourne. *J Relig Health.* 2019*;* 58: 343–355. (ePub 2018). <https://doi.org/10.1007/s10943-018-0673-4> | Accessed 9/20/24 |  |  |
| **Kagin RS** | **2010** | [Kagin, R. S. The Relationship between Music Therapists’ Spiritual Beliefs and Clinical Practice. Query date: 2023-06-28 12:26:39. search.proquest.com, 2010. https://search.proquest.com/openview/dcdf3ee7a5d8519b812d4118bff6164c/1?pq-origsite=gscholar&cbl=18750.](https://search.proquest.com/openview/dcdf3ee7a5d8519b812d4118bff6164c/1?pq-origsite=gscholar&cbl=18750) | Accessed 9/20/24 |  |  |
| **Kang KK** | **2020** | *Kang, KK, and N. Moran. “Experiences of Inpatient Staff Meeting the Religious and Cultural Needs of BAME Informal Patients and Patients Detained under the Mental Health Act 1983.” MENTAL HEALTH REVIEW JOURNAL, vol. 25, no. 2, 2020, pp. 113–25,* [*https://doi.org/10.1108/MHRJ-11-2019-0041. WOS:000543490700001.*](https://doi.org/10.1108/MHRJ-11-2019-0041) | Accessed 9/20/24 |  |  |
| **Karvinen I** | **2009** | [Karvinen, I. Henkinen Ja Hengellinen Terveys–Etnografinen Tutkimus Kendun Sairaalan Henkilökunnan Ja Potilaiden Sekä Kendu Bayn Kylän Asukkaiden Henkisen Ja …. erepo.uef.fi, 2009, https://erepo.uef.fi/bitstream/handle/123456789/9036/urn_isbn_978-951-27-1208-3.pdf?sequence=1.](https://erepo.uef.fi/bitstream/handle/123456789/9036/urn_isbn_978-951-27-1208-3.pdf?sequence=1) | Accessed 9/20/24 | Finnish | No |
| **Kellaher K** | **2021** | [Kellaher, K. How Servant Leadership Influences Healthcare’s Value-Based Care. //, https://search.proquest.com/openview/74c26a7cf4de6ea8fc46297ebcfe569a/1?pq-origsite=gscholar&cbl=18750&diss=y.](https://search.proquest.com/openview/74c26a7cf4de6ea8fc46297ebcfe569a/1?pq-origsite=gscholar&cbl=18750&diss=y) | Accessed 9/20/24 |  |  |
| **Kemp AJ** | **2008** | [Kemp, A. J. Quality of Life and the Health Care System in New River Valley, Virginia: Residents’ Perceptions and Experiences. vtechworks.lib.vt.edu, 2008, https://vtechworks.lib.vt.edu/handle/10919/26611 https://vtechworks.lib.vt.edu/bitstream/handle/10919/26611/Kemp_FINAL_ETD.pdf.](https://vtechworks.lib.vt.edu/bitstream/handle/10919/26611/Kemp_FINAL_ETD.pdf) | Accessed 9/21/24 |  |  |
| **Khan IJ** | **2019** | [Khan IJ. Spirituality and Religion-relevance and Assessment in the Clinical Setting. Current Psychiatry Research and Reviews, 2019, 15, 80-87](https://doi.org/10.2174/1573400515666190404143104)**,** | Accessed 9/20/24 |  |  |
| **Kimball SL** | **2022** | Kimball, SL, et al. “Embedding Chaplaincy Services in Primary Care for Immigrants, Refugees and Asylum Seekers: A Boston Pilot Intervention.” JOURNAL OF RELIGION & HEALTH, vol. 62, no. 1, Feb. 2023, pp. 55–64.  <https://doi.org/10.1007/s10943-022-01568-8> | Accessed 1/11/25 |  |  |
| **King DE** | **2006** | [King, DE. “Spirituality, Health, and Medical Care of Adults.” SOUTHERN MEDICAL JOURNAL, vol. 99, no. 10, Oct. 2006, pp. 1166–67, https://doi.org/10.1097/01.smj.0000242809.99420.e0. WOS:000244072800040.](https://doi.org/10.1097/01.smj.0000242809.99420.e0) | Accessed 9/20/24 |  |  |
| **King DE** | **2007** | [King, DE, and J. Crisp. “Case Discussion: Do Not Neglect the Spiritual History.” SOUTHERN MEDICAL JOURNAL, vol. 100, no. 4, Apr. 2007, pp. 426–426, https://doi.org/10.1097/SMJ.0b013e31803169dd. WOS:000246777600033.](https://doi.org/10.1097/SMJ.0b013e31803169dd) | Accessed 9/20/24 |  |  |
| **King DE** | **2004** | King DE, Blue A, Mallin R, Thiedke C: Implementation and assessment of a spiritual history taking curriculum in the first year of medical school. *Teach Learn Med* 2004;16:64–68. doi: 10.1207/s15328015tlm1601_13. PMID: 14987177.  <https://doi.org/10.1207/s15328015tlm1601_13> | Accessed 9/20/24 |  |  |
| **Klimasinki MJ** | **2021** | [Klimasinski, MW. “Spiritual Care in the Intensive Care Unit.” ANAESTHESIOLOGY INTENSIVE THERAPY, vol. 53, no. 4, 2021, pp. 350–57, https://doi.org/10.5114/ait.2021.109920. WOS:000714677200011.](https://doi.org/10.5114/ait.2021.109920) | Accessed 9/7/24 | Polish | No |
| **Koch DC** | **2011** | *Koch, K. D., and C. Feudtner. “Spiritual Dimensions.” Palliative Care for Infants, Children, and …, //, https://books.google.com/books?hl=en&lr=&id=WaAzS1GlvCMC&oi=fnd&pg=PA227&ots=95nbo_S32q&sig=fybRVWAbJyMy5ai3tGRZk05Kq3I https://hamdir.ir/wp-content/uploads/2021/10/Palliative-Care-for-Infants-Children-and-Adolescents.pdf#page=244.* | Accessed (both links) 9/20/24 |  |  |
| **Koenig G** | **2001** | [*Koenig, HG. “Spiritual Assessment in Medical Practice.” AMERICAN FAMILY PHYSICIAN, vol. 63, no. 1, Jan. 2001, pp. 30-+. WOS:000166414400001.*](https://pubmed.ncbi.nlm.nih.gov/11195767/) | Accessed 9/20/24 |  |  |
| **Koster M** | **2010** | Koster M. Zingeving in het consult van de huisarts. University of Groningen Faculty of Theology and Religious Studies. Thesis - Master of Spiritual Care. 2010  <https://rcs.studenttheses.ub.rug.nl/190/1/0910-GV%20%20KOSTER%20M.%20Ma-scriptie.pdf> | Accessed 9/21/24 | Dutch | Brief translation  Dutch |
| **Kraft M** | **2011** | [Kraft, M. “Spirituality and Medicine.” Physicians’ Pathways to Non-Traditional Careers and …, //. https://doi.org/10.1007/978-1-4614-0551-1_30.](https://doi.org/10.1007/978-1-4614-0551-1_30) | Accessed 9/20/24 |  |  |
| **Kretzer K** | **2005** | [Kretzer, K. “The Hypertension and Self Identity through Hoʻoponopono Study in Hawaiʻi,” //. https://scholarspace.manoa.hawaii.edu/bitstream/10125/11764/2/uhm_phd_4624_r.pdf.](https://scholarspace.manoa.hawaii.edu/bitstream/10125/11764/2/uhm_phd_4624_r.pdf) | Accessed 9/20/24 |  |  |
| **Kuckel DP** | **2022** | [*Kuckel, DP, et al. “The Spiritual Assessment.” AMERICAN FAMILY PHYSICIAN, vol. 106, no. 4, Oct. 2022, pp. 415–19. WOS:000886956600018.*](https://pubmed.ncbi.nlm.nih.gov/36260898/) | Accessed 9/20/24 |  |  |
| **Kuhl CD** | **2005** | *Kuhl, C. D. Spiritually Commanding an Air Force Squadron in the 21st Century. apps.dtic.mil, 2005, https://apps.dtic.mil/sti/citations/ADA476165 https://apps.dtic.mil/sti/pdfs/ADA476165.pdf.* | Accessed 9/20/24 |  |  |
| **Laker CS** | **2022** | [Laker, C. S. An Exploration of the Perceptions and Practice of Community Nursing Staff within Two Care Settings in Relation to Spiritual Distress at the End of Life. //, https://repository.canterbury.ac.uk/download/ff095e3600a24d55aecb1985447e5601b59490770009be40abe938892c6a9554/3132758/Thesis%20re-submission%20with%20highlighted%20changes%20%28Clive%20Laker%29.pdf.](https://repository.canterbury.ac.uk/download/ff095e3600a24d55aecb1985447e5601b59490770009be40abe938892c6a9554/3132758/Thesis%20re-submission%20with%20highlighted%20changes%20%28Clive%20Laker%29.pdf) | Accessed 9/20/24 |  |  |
| **Larson K** | **2003** | [Larson, K. “The Importance of Spiritual Assessment: One Clinician’s Journey.” GERIATRIC NURSING 24, no. 6 (November 2003): 370–71. https://doi.org/10.1016/j.gerinurse.2003.10.020.](https://doi.org/10.1016/j.gerinurse.2003.10.020) | Accessed 9/20/24 |  |  |
| **Lee E** | **2020** | [Lee, E., et al. “The Relationship between Caregivers’ Perceptions of End-of-Life Care in Long-Term Care and a Good Resident Death.” PALLIATIVE & SUPPORTIVE CARE, vol. 18, no. 6, Dec. 2020, pp. 683–90, https://doi.org/10.1017/S1478951520000292. WOS:000604909900009.](https://doi.org/10.1017/S1478951520000292) | Accessed 9/20/24 |  |  |
| **Levitt C** | **2006** | *Levitt, C. “The Spiritual Side of Health Is Very Often Neglected in Tradi-Tional Practice and Training Programs in Medicine. We Tend to Focus on Biomedical Sciences and Behavioural ….” Spirituality and Health: Multidisciplinary Explorations, //. https://books.google.com/books?hl=en&lr=&id=zud0CwAAQBAJ&oi=fnd&pg=PA61&ots=MvtC_Cicxy&sig=0-IvprWktc2UsmKOMpwUgI6lsnY.* | Accessed 9/20/24 |  |  |
| **Lind B** | **2011** | Lind B, Sendelbach S, Steen S. Effects of a Spirituality Training Program for Nurses on Patients in a Progressive Care Unit. *Crit Care Nurse*. 2011; 31(3):87-90. doi: <https://doi.org/10.4037/ccn2011372> | Accessed 9/20/24 |  |  |
| **Loustalot F** | **2008** | [Loustalot, “Assessing Patients’ Spiritual Needs.” Kai Tiaki Nursing New Zealand. 2008, Vol.14(8), p.21.](https://pubmed.ncbi.nlm.nih.gov/18822557/) (accessed via Brown Library) | Accessed 9/20/24 |  |  |
| **Loynes B** | **2015** | [Loynes, B., and J. O’Hara. “How Can Mental Health Clinicians, Working in Intellectual Disability Services, Meet the Spiritual Needs of Their Service Users?” Advances in Mental Health and Intellectual …, //, https://doi.org/10.1108/AMHID-10-2014-0035.](https://doi.org/10.1108/AMHID-10-2014-0035) | Accessed 9/20/24 |  |  |
| **Lucchetti G** | **2013** | Lucchetti, G, RM Bassi, and ALG Lucchetti. “TAKING SPIRITUAL HISTORY IN CLINICAL PRACTICE: A SYSTEMATIC REVIEW OF INSTRUMENTS.” EXPLORE-THE JOURNAL OF SCIENCE AND HEALING 9, no. 3 (May 2013): 159–70. https://doi.org/10.1016/j.explore.2013.02.004[.](https://doi.org/10.1016/j.explore.2013.02.004) | Accessed 9/21/24 |  |  |
| **Lucchetti G** | **2012** | [Lucchetti, G., et al. Integrating Spirituality into Primary Care. Citeseer, 2012, https://citeseerx.ist.psu.edu/document?repid=rep1&type=pdf&doi=69d784e71c9dbba41b9a4bc5f3c5da381446b14b.](https://citeseerx.ist.psu.edu/document?repid=rep1&type=pdf&doi=69d784e71c9dbba41b9a4bc5f3c5da381446b14b) | Accessed 9/20/24 |  |  |
| **Martins VRF** | **2011** | Martins, V. R. F. Caracterização e Abordagem Das Necessidades Espirituais Do Doente Em Final de Vida: Visão Integrada Dos Profissionais de Cuidados Paliativos.  <https://repositorio.ul.pt/handle/10451/6290> | Accessed  9/21/24 | Portuguese | Brief translation  Portuguese |
| **Mastrangelo S** | **2023** | Mastrangelo S. Mastrangelo S, Rochat E, Pruijm M. Exploration of the Spiritual Expectations of Patients in a Swiss Hemodialysis Center. *Kidney and Dialysis*. 2025; 5(1):2 (e-pub ahead of print 2023). <https://doi.org/10.3390/kidneydial5010002> | Accessed 1/11/25 |  |  |
| **Maus K** | **2021** | Maus et al., “Resilienz, Kohärenz, Lebenssinn Sowie Andere Konzepte Und Begriffe in Der Palliativversorgung–Eine Standortbestimmung.”  Spiritual Care 2021;10(2):145-155  <https://doi.org/10.1515/spircare-2020-0121> | Accessed 9/7/24 | German | Brief translation  German |
| **McClane KS** | **2006** | [McClane, KS. “Screening Instruments for Use in a Complete Geriatric Assessment.” CLINICAL NURSE SPECIALIST, vol. 20, no. 4, July 2006, pp. 201–07, https://doi.org/10.1097/00002800-200607000-00010. WOS:000239420000008.](https://doi.org/10.1097/00002800-200607000-00010) | Accessed 9/20/24 |  |  |
| **McCormick TR** | **2012** | [McCormick, T. R., et al. “Ethical and Spiritual Concerns near the End of Life.” Journal of Religion …, //, https://doi.org/10.1080/15528030.2012.698385.](https://doi.org/10.1080/15528030.2012.698385) | Accessed 9/20/24 |  |  |
| **McCormick TR** | **2014** | [McCormick, T. R., and D. Min. “Spirituality and Medicine.” Ethics in Medicine, //. https://depts.washington.edu/bhdept/ethics-medicine/bioethics-topics/detail/79.](https://depts.washington.edu/bhdept/ethics-medicine/bioethics-topics/detail/79) | Accessed 9/20/24 |  |  |
| **McEachron G** | **2014** | [McEachron, G. “Compassion for the Young Experiencing the Trauma of Death.” Journal of Child &Adolescent Trauma, //, https://doi.org/10.1007/s40653-014-0005-0.](https://doi.org/10.1007/s40653-014-0005-0) | Accessed 9/20/24 |  |  |
| **McLean HJ** | **2009** | [McLean, H. J. “ONE MORE HAT IN THE RING: A COMPARATIVE SYNOPSIS AND NEW TOOL FOR PATIENT SPIRITUAL ASSESSMENT.” Journal of Pastoral Counseling, //, https://search.ebscohost.com/login.aspx?direct=true&profile=ehost&scope=site&authtype=crawler&jrnl=0449508X&asa=Y&AN=55750430&h=oQd9OvHaZ24QvYS5JPORYNwfuJxUpNwLfQd2fvDHpM4dGKP%2BxKa0633OSuUt%2Flf2ZhlpBQmMj%2B8EqBbO2cdwCA%3D%3D&crl=c](https://search.ebscohost.com/login.aspx?direct=true&profile=ehost&scope=site&authtype=crawler&jrnl=0449508X&asa=Y&AN=55750430&h=oQd9OvHaZ24QvYS5JPORYNwfuJxUpNwLfQd2fvDHpM4dGKP%2BxKa0633OSuUt%2Flf2ZhlpBQmMj%2B8EqBbO2cdwCA%3D%3D&crl=c%20https://search.ebscohost.com/login.aspx?direct=true&profile=ehost&scope=site&authtype=crawler&jrnl=0449508X&asa=Y&AN=55750430&h=oQd9OvHaZ24QvYS5JPORYNwfuJxUpNwLfQd2fvDHpM4dGKP%2BxKa0633OSuUt%2Flf2ZhlpBQmMj%2B8EqBbO2cdwCA%3D%3D&crl=f) | Accessed 9/20/24 |  |  |
| **McNair T** | **2018** | [McNair, T. Nurses’ Perspectives On Spiritual Care And Its Connection To Healing.](https://digitalrepository.salemstate.edu/handle/20.500.13013/659%20https:/digitalrepository.salemstate.edu/bitstream/handle/20.500.13013/659/auto_convert.pdf?sequence=3&isAllowed=y)  <https://digitalrepository.salemstate.edu/bitstream/handle/20.500.13013/659/auto_convert.pdf?sequence=3&isAllowed=y> | Accessed 9/21/24 |  |  |
| **McSherry W** | **2019** | [McSherry, W., et al. “Spiritual Assessment in Healthcare: An Overview of Comprehensive, Sensitive Approaches to Spiritual Assessment for Use within the Interdisciplinary Healthcare Team.” Spirituality in Healthcare …, //, https://doi.org/10.1007/978-3-030-04420-6_3.](https://doi.org/10.1007/978-3-030-04420-6_3) | Accessed 09/20/24 |  |  |
| **McSherry W** | **2010** | [McSherry, W. “Spiritual Assessment: Definition, Categorisation and Features.” Spiritual Assessment in Healthcare Practice, //, https://books.google.com/books?hl=en&lr=&id=7SK7qbvU1hIC&oi=fnd&pg=PA57&ots=ak2pQq9Yvq&sig=TjX8LpQ1P1SU2oPajG1BcU0e6Sw.](https://books.google.com/books?hl=en&lr=&id=7SK7qbvU1hIC&oi=fnd&pg=PA57&ots=ak2pQq9Yvq&sig=TjX8LpQ1P1SU2oPajG1BcU0e6Sw) | Accessed 09/20/24 |  |  |
| **Memaryan N** | **2021** | Memaryan N, Rasouli M, Ghaempanah Z, Mehrabi M. (2021). An Islamic Model for Taking Patients’ Spiritual History: Islamic Model of Taking Spiritual History. *Bioethics and Health Law Journal (BHL)*. 2021;1(1):1–8 (e23). <https://doi.org/10.22037/bhl.v1i1.38185> | Accessed 9/21/24 |  |  |
| **Mendoza MD** | **2017** | [Mendoza, M. D., and M. Lopez. “Culture, Race, and Ethnicity Issues in Health Care.” Family Medicine: Principles and Practice, //, https://www.researchgate.net/profile/Michael-Mendoza-9/publication/307143164_Culture_Race_and_Ethnicity_Issues_in_Health_Care/links/5d4aac64299bf1995b6ab38a/Culture-Race-and-Ethnicity-Issues-in-Health-Care.pdf.](https://www.researchgate.net/profile/Michael-Mendoza-9/publication/307143164_Culture_Race_and_Ethnicity_Issues_in_Health_Care/links/5d4aac64299bf1995b6ab38a/Culture-Race-and-Ethnicity-Issues-in-Health-Care.pdf) | Accessed 09/20/24 |  |  |
| **Mennie BA** | **2011** | [Mennie, B. A. Sojourning with the Spirit in Recovery from Mental Illness. era.library.ualberta.ca, 2011, https://era.library.ualberta.ca/items/749fe821-fe78-4390-a386-994e2f100255 https://era.library.ualberta.ca/items/749fe821-fe78-4390-a386-994e2f100255/download/0cad2c80-7539-4935-b441-4e590dfc7847.](https://era.library.ualberta.ca/items/749fe821-fe78-4390-a386-994e2f100255/download/0cad2c80-7539-4935-b441-4e590dfc7847) | Accessed 9/21/24 both links |  |  |
| **Messina G** | **2018** | [Messina, G. “Spirituality and Cancer Disease: A Study on the Efficacy of Antitumor Therapies with Natural Anticancer Agents in Relation to the Spiritual Profile,” //. https://aisberg.unibg.it/retrieve/e40f7b88-3dba-afca-e053-6605fe0aeaf2/TDUnibg_Messina-Giuseppina.pdf.](https://aisberg.unibg.it/retrieve/e40f7b88-3dba-afca-e053-6605fe0aeaf2/TDUnibg_Messina-Giuseppina.pdf) | Accessed 09/20/24 |  |  |
| **Mezger M** | **2018** | [Mezger, M. Religion, Spiritualität, Medizin: Alternative Religiosität Und Palliative Care in Der Schweiz. library.oapen.org, 2018, https://library.oapen.org/bitstream/handle/20.500.12657/27380/1/9783839441657.pdf.](https://library.oapen.org/bitstream/handle/20.500.12657/27380/1/9783839441657.pdf) | Accessed 09/20/24 | German | No |
| **Miller DR** | **2019** | Miller DR. Spiritual, religious and existential dimentions of care. In. Sumser, B., and W. Muller. Palliative Care: A Guide for …, //, [https://books.google.com/books?hl=en&lr=&id=BmmtDwAAQBAJ&oi=fnd&pg=PA122&ots=GW6k8fFUlU&sig=DBoOI_Gwq3EEpdM_k1lb8WRXRvU. Palliative Care: A Guide for Health Social Workers. United Kingdom, Oxford University Press, 2019.](https://books.google.com/books?hl=en&lr=&id=BmmtDwAAQBAJ&oi=fnd&pg=PA122&ots=GW6k8fFUlU&sig=DBoOI_Gwq3EEpdM_k1lb8WRXRvU) | Accessed 09/20/24 |  |  |
| **Miller L** | **2015** | *Miller, L. Health Care Clinicians’ Compliance with Conducting Spiritual Assessments and Providing Spiritual Care to Infertile Women. //,* [*https://search.proquest.com/openview/9f46a17004b8b32bbc78512ecb311758/1?pq-origsite=gscholar&cbl=18750.*](https://search.proquest.com/openview/9f46a17004b8b32bbc78512ecb311758/1?pq-origsite=gscholar&cbl=18750) | Accessed 09/20/24 |  |  |
| **Milligan S** | **2011** | *Milligan, S. “Addressing the Spiritual Care Needs of People near the End of Life.” Nursing Standard (through 2013), //,* [*https://search.proquest.com/openview/ad0be88c56301bf17aa7b7aa4488e2ce/1?pq-origsite=gscholar&cbl=30130.*](https://search.proquest.com/openview/ad0be88c56301bf17aa7b7aa4488e2ce/1?pq-origsite=gscholar&cbl=30130) | Accessed 09/20/24 |  |  |
| **Milstein G** | **2017** | Milstein G, Middel D, & Espinosa A. Consumers, clergy, and clinicians in collaboration: Ongoing implementation and evaluation of a mental wellness program. *Am J Psych Rehab*. 2017; 20(1), 34–61.  [*http://dx.doi.org/10.1080/15487768.2016.1267052*](http://dx.doi.org/10.1080/15487768.2016.1267052) | Accessed 1/11/25 |  |  |
| **Mitchell D** | **2004** | [Mitchell and Gordon, “Making Sense of Spiritual Care.” https://books.google.com/books?hl=en&lr=&id=J45ZxweFcnUC&oi=fnd&pg=PA65&ots=9cvZIHt9_N&sig=UarA5Nzpc-P_F58030GVCa1L0fo#v=onepage&q&f=false](https://books.google.com/books?hl=en&lr=&id=J45ZxweFcnUC&oi=fnd&pg=PA65&ots=9cvZIHt9_N&sig=UarA5Nzpc-P_F58030GVCa1L0fo#v=onepage&q&f=false) | Accessed 09/20/24 |  |  |
| **Mizock L** | **2012** | [Mizock, L., et al. “Spiritual and Religious Issues in Psychotherapy with Schizophrenia: Cultural Implications and Implementation.” RELIGIONS, vol. 3, no. 1, Mar. 2012, pp. 82–98, https://doi.org/10.3390/rel3010082. WOS:000321253400005.](https://doi.org/10.3390/rel3010082) | Accessed 09/20/24 |  |  |
| **Mohr S** | **2009** | [Mohr, S, and P Huguelet. “The Relationship between Schizophrenia and Religion and Its Implications for Care.” SWISS MEDICAL WEEKLY 134, no. 25–26 (June 26, 2004): 369–76. https://smw.ch/index.php/smw/article/view/385/382](https://smw.ch/index.php/smw/article/view/385/382) | Accessed 09/20/24 |  |  |
| **Montazeri M** | **2017** | Maryam Montazeri, Fariba Borhani, Saeid Nazari Tavakkoli, Spirituality in Treatment and Medical Ethics, J Res Med Dent Sci, 2017, 5 (6): 131-136, [DOI: 10.24896/jrmds.20175623](https://www.jrmds.in/articles/spirituality-in-treatment-and-medical-ethics.pdf) | Accessed 09/20/24 |  |  |
| **Morgan G** | **2017** | [Morgan, G. Independent Advocacy and Spiritual Care. Springer, 2017, https://doi.org/10.1057/978-1-137-53125-4.](https://doi.org/10.1057/978-1-137-53125-4) | Accessed 09/20/24 |  |  |
| **Motl J** | **2016** | Motl, J. Spiritualita a Duševní Nemoc: Role Spirituality v Životě Lidí Se Zkušeností s Psychotickým Onemocněním. dspace.cuni.cz, 2016, https://dspace.cuni.cz/handle/20.500.11956/78575  <https://dspace.cuni.cz/bitstream/handle/20.500.11956/78575/140048882.pdf?sequence=1>. | Accessed 1/11/25 | Czech | No |
| **Mueller PS** | **2001** | Mueller, PS, et al. “Religious Involvement, Spirituality, and Medicine: Implications for Clinical Practice.” MAYO CLINIC PROCEEDINGS, vol. 76, no. 12, Dec. 2001, pp. 1225–35, https://doi.org/10.4065/76.12.1225. WOS:000172500700008. | Accessed 9/21/24 |  |  |
| **Nagy BM** | **2008** | Nagy, BM. The part of spirituality in coping with chronic illness.  <https://www.researchgate.net/publication/240971088_The_part_of_spirituality_in_the_coping_with_chronic_illnesses>  Mentálhigiéné És Pszichoszomatika, //, <https://akjournals.com/view/journals/0406/10/1/article-p21.xml>. | Accessed 9/7/24 | Hungarian | Brief translation  Hungarian |
| **Nolan S** | **2015** | [Nolan, S. “Healthcare Chaplains Responding to Change: Embracing Outcomes or Reaffirming Relationships?” Health and Social Care Chaplaincy, //, https://journal.equinoxpub.com/HSCC/article/view/15315.](https://journal.equinoxpub.com/HSCC/article/view/15315) | Accessed 09/20/24 |  |  |
| **Norris L** | **2013** | [Norris, L., et al. “Communicating about Spiritual Issues with Cancer Patients.” New Challenges in Communication …, //, https://doi.org/10.1007/978-1-4614-3369-9_8.](https://doi.org/10.1007/978-1-4614-3369-9_8) | Accessed 09/20/24 |  |  |
| **Odier C** | **2004** | *Odier, C. “Accompagnement Spirituel Ou «faire Passer Un Chameau Par Le Trou d’une Aiguille…».” Frontieres. 2004.17(1). DOI : https://doi.org/10.7202/1073609ar*  <https://www.erudit.org/fr/revues/fr/2004-v17-n1-fr05676/1073609ar/> | *Accessed 9/7/24* | French | Brief translation  French |
| **Oji V** | **2010** | [Oji, V. “Mind, Medications &mental Disorders: A Spiritual Approach.” Journal of Christian Nursing, //, https://journals.lww.com/journalofchristiannursing/fulltext/2010/04000/Mind,_Medications___Mental_Disorders__A_Spiritual.10.aspx.](https://journals.lww.com/journalofchristiannursing/fulltext/2010/04000/Mind,_Medications___Mental_Disorders__A_Spiritual.10.aspx) | Accessed 09/20/24 |  |  |
| **Oliveira JAC** | **2018** | [Oliveira, J. A. C. Desafios Do Cuidado Integral Em Saúde: A Dimensão Espiritual Do Médico Se Relaciona Com Sua Prática Na Abordagem Espiritual Do Paciente? teses.usp.br, 2018,](https://www.teses.usp.br/teses/disponiveis/5/5137/tde-28092018-083224/en.php%20https:/www.teses.usp.br/teses/disponiveis/5/5137/tde-28092018-083224/publico/JanaineAlineCamargoOliveiraVersaoCorrigida.pdf)  <https://www.teses.usp.br/teses/disponiveis/5/5137/tde-28092018-083224/pt-br.php> | Accessed 9/21/24 | Portuguese | **Yes**  **Full Version**  **Portuguese** |
| **Opatrny M** | **2021** | Opatrný, M. III. 2. Spiritual Assessment in Social Work. In (Ed) Gehrig RB et al. Spirituality, Ethics and Social Work. 1st edition, Freiburg: FreiDoc plus, 2021 ISBN: 978-3-928969-86-4 DOI: 10.6094/978-3-928969-86-4  Opatrný, M. “III. 2. La Evaluación de La Espiritualidad En El Trabajo Social.” Espiritualidad, Ética y Trabajo Social, <https://d-nb.info/1242409475/34#page=137>.  Opatrný, M. “Einschätzung Der Spiritualität in Der Sozialen Arbeit.” Deutsche Nationalbibliothek.  <https://www.researchgate.net/publication/356645874_Einschatzung_der_Spiritualitat_in_der_Sozialen_Arbeit>  Opatrný, M. La spiritualite, l’ethique et le travail social.  <https://www.researchgate.net/publication/361417483_La_spiritualite_l'ethique_et_le_travail_social> | *Accessed 9/7/24* | *German*  *French*  *Spanish*  *English* | **Yes**  **Full Version**  **German**  **French**  **Spanish** |
| **Oswald KD** | **2004** | [Oswald, K. D. Nurses’ Perceptions of Spirituality and Spiritual Care. search.proquest.com, 2004, https://search.proquest.com/openview/30b2ccc4a3665c1c683c929066981635/1?pq-origsite=gscholar&cbl=18750&diss=y.](https://search.proquest.com/openview/30b2ccc4a3665c1c683c929066981635/1?pq-origsite=gscholar&cbl=18750&diss=y) | Accessed 09/20/24 |  |  |
| **Oxhandler HK** | **2017** | [Oxhandler, HK, and DE Parrish. “Integrating Clients’ Religion/Spirituality in Clinical Practice: A Comparison among Social Workers, Psychologists, Counselors, Marriage and Family Therapists, and Nurses.” JOURNAL OF CLINICAL PSYCHOLOGY, vol. 74, no. 4, Apr. 2018, pp. 680–94, https://doi.org/10.1002/jclp.22539. WOS:000428330400013.](https://doi.org/10.1002/jclp.22539) | Accessed 09/20/24 |  |  |
| **Panitz GO** | **2018** | [Panitz, G. O., et al. “Instrumentos de Abordagem Da Espiritualidade Na Prática Clínica.” Acta Méd.(Porto …, //, https://ebooks.pucrs.br/edipucrs/acessolivre/periodicos/acta-medica/assets/edicoes/2018-1/arquivos/pdf/3.pdf.](https://ebooks.pucrs.br/edipucrs/acessolivre/periodicos/acta-medica/assets/edicoes/2018-1/arquivos/pdf/3.pdf) | Accessed  1/16/25 | Portuguese | No |
| **Parada ML** | **2022** | [Parada, ML. “Integrating Religion/Spirituality into Professional Social Work Practice.” JOURNAL OF RELIGION AND SPIRITUALITY IN SOCIAL WORK, vol. 41, no. 4, Oct. 2022, pp. 351–68, https://doi.org/10.1080/15426432.2022.2102102. WOS:000831171500001.](https://doi.org/10.1080/15426432.2022.2102102) | Accessed 09/20/24 |  |  |
| **Park DM** | **2005** | [Park, D. M. “The Health of the Healer: Physician/Health-Care Provider Wellness.” Integrative Oncology: Principles and Practice, //. https://books.google.com/books?hl=en&lr=&id=_uugeo02N04C&oi=fnd&pg=PA57&ots=wmhZMzu2sn&sig=9YOQgiLX7ecMYHGKYKDLQS-sCLU.](https://books.google.com/books?hl=en&lr=&id=_uugeo02N04C&oi=fnd&pg=PA57&ots=wmhZMzu2sn&sig=9YOQgiLX7ecMYHGKYKDLQS-sCLU) | Accessed 09/20/24 |  |  |
| **Parker AR** | **2019** | [Parker, A. R. Spiritually Informed Art Therapy: An Inquiry into Formation. Query date: 2023-06-28 16:19:16. era.library.ualberta.ca, 2019. https://era.library.ualberta.ca/items/665f7ebc-31ec-4ec1-a655-9ddbc8ae3225 https://era.library.ualberta.ca/items/665f7ebc-31ec-4ec1-a655-9ddbc8ae3225/download/de1c2e95-3b1b-44ab-af24-8da6f993825a.](https://era.library.ualberta.ca/items/665f7ebc-31ec-4ec1-a655-9ddbc8ae3225%20https:/era.library.ualberta.ca/items/665f7ebc-31ec-4ec1-a655-9ddbc8ae3225/download/de1c2e95-3b1b-44ab-af24-8da6f993825a) | Accessed 09/21/24 |  |  |
| **Paswan RK** | **2019** | Paswan RK, Pushp B, Aswal S, & Motwani Y. (2019). Use spirituality related beliefs in treatment of psychiatric illness. *International Journal of Medical and Biomedical Studies*. 2019; *3*(10): 227-229. <https://doi.org/10.32553/ijmbs.v3i10.662> | Accessed 9/21/24 |  |  |
| **Patton L** | **2018** | [Patton, L. A. Education and Standardized Discussion Guides to Enhance Nurses’ Spiritual Care Practices in the Medical Intensive Care Unit. rave.ohiolink.edu, 2018, https://rave.ohiolink.edu/etdc/view?acc_num=casednp1519836081431734](https://rave.ohiolink.edu/etdc/view?acc_num=casednp1519836081431734) | Accessed 9/21/24 |  |  |
| **Pawlikowski J** | **2013** | [Pawlikowski, J. Problemy Metodologiczne w Badaniach Nad Związkiem Pomiędzy Religijnością a Zdrowiem.](https://repozytorium.amu.edu.pl/handle/10593/8424%20http:/repozytorium.amu.edu.pl:8080/bitstream/10593/8424/1/Jakub%20Pawlikowski%20Problemy_metodologiczne_w_badaniach_nad_zwi%C4%85zkiem_pomi%C4%99dzy_religijno%C5%9Bci%C4%85_a_zdrowiem.pdf)  <https://repozytorium.amu.edu.pl/items/01f0a64a-bd84-4033-b980-f35b3117afce>  chrome-extension://efaidnbmnnnibpcajpcglclefindmkaj/https://repozytorium.amu.edu.pl/server/api/core/bitstreams/0f5915e6-e17b-4bfb-8112-79948f2b7952/content | Accessed 9/7/24 | Polish | No |
| **Payman V** | **2016** | [Payman, V. “The Importance of Taking a Religious and Spiritual History.” AUSTRALASIAN PSYCHIATRY, vol. 24, no. 5, Oct. 2016, pp. 434–36, https://doi.org/10.1177/1039856216647257. WOS:000384836800008.](https://doi.org/10.1177/1039856216647257) | Accessed 9/20/24 |  |  |
| **Pembroke NF** | **2008** | [Pembroke, NF. “Appropriate Spiritual Care by Physicians: A Theological Perspective.” JOURNAL OF RELIGION & HEALTH, vol. 47, no. 4, Dec. 2008, pp. 549–59, https://doi.org/10.1007/s10943-008-9183-0. WOS:000260511000010.](https://doi.org/10.1007/s10943-008-9183-0) | Accessed 9/20/24 |  |  |
| **Pennaertz R** | **2013** | Pennaertz R. Spirituele diagnostiek: Diagnostische instrumenten voor primaire zorgverleners in de palliatieve zorg in Nederland. Masters Thesis. University of Groningen. Faculty of Theology and Religious Studies. 2013.  <https://ggw.studenttheses.ub.rug.nl/118/1/1314-GV%20%20%20PENNAERTS%20R.%20%20Ma-scriptie%20%20definitieve%20versie.pdf> | Accessed 1/16/25 | Dutch | No  (full version is in English) |
| **Pennington CG** | **2019** | *Pennington, C. G. “AChristian PERSPECTIVE ON RETURNING TO.” Researchgate.Net, https://www.researchgate.net/profile/Colin-Pennington/publication/335841162_A_CHRISTIAN_PERSPECTIVE_ON_RETURNING_TO_HEALTH_AND_WELLNESS/links/5d7fb50ea6fdcc66b0009981/A-CHRISTIAN-PERSPECTIVE-ON-RETURNING-TO-HEALTH-AND-WELLNESS.pdf.* | *Accessed 9/20/24* |  |  |
| **Perez M** | **2005** | [Perez, M. “The Role of Spirituality in the Recovery Process of Schizophrenia: A Multiple Case Study,” //. https://search.proquest.com/openview/21beccbcda6143562fba8f4e4e764f0b/1?pq-origsite=gscholar&cbl=18750&diss=y.](https://search.proquest.com/openview/21beccbcda6143562fba8f4e4e764f0b/1?pq-origsite=gscholar&cbl=18750&diss=y) | Accessed 9/20/24 |  |  |
| **Perkins HS** | **2016** | [Perkins, H. S., and H. S. Perkins. “The ‘Right’ Time and Way to Die.” A Guide to Psychosocial and Spiritual Care at …, //. https://doi.org/10.1007/978-1-4939-6804-6_11.](https://doi.org/10.1007/978-1-4939-6804-6_11) | Accessed 9/20/24 |  |  |
| **Perry BS** | **2016** | [Perry, B. S. Assessing Knowledge and Attitudes Toward Spirituality and Spiritual Care in APRN Students. digitalcommons.ric.edu, 2016, https://digitalcommons.ric.edu/etd/151/ https://digitalcommons.ric.edu/cgi/viewcontent.cgi?article=1152&context=etd.](https://digitalcommons.ric.edu/etd/151/%20https:/digitalcommons.ric.edu/cgi/viewcontent.cgi?article=1152&context=etd) |  |  |  |
| **Piderman KM** | **2011** | [Piderman, K. M., et al. “Spiritual Well-Being and Spiritual Practices in Elderly Depressed Psychiatric Inpatients.” Journal of Pastoral …, //, https://doi.org/10.1177/154230501106500103.](https://doi.org/10.1177/154230501106500103) | Accessed 9/20/24 |  |  |
| **Piotrowski LF** | **2013** | Piotrowski LF. Advocating and Educating for Spiritual Screening Assessment and Referrals to Chaplains. *OMEGA - Journal of Death and Dying*. 2013; 67(1-2): 185-192. <https://doi.org/10.2190/OM.67.1-2.v> | Accessed 9/20/24 |  |  |
| **Power BA** | **2011** | [Powers, B. A., and N. M. Watson. “Spiritual Nurturance and Support for Nursing Home Residents with Dementia.” Dementia, //, https://doi.org/10.1177/1471301210392980.](https://doi.org/10.1177/1471301210392980) | Accessed 9/20/24 |  |  |
| **Precoma DB** | **2019** | Précoma, D. B., et al. “Atualização Da Diretriz de Prevenção Cardiovascular Da Sociedade Brasileira de Cardiologia-2019.”  Updated Cardiovascular Prevention Guideline of the Brazilian Society of Cardiology – 2019. Brazilian Society of Cardiology (Sociedade Brasileira de Cardiologia – SBC).  <http://publicacoes.cardiol.br/portal/abc/portugues/aop/2019/aop-diretriz-prevencao-cardiovascular-portugues.pdf> | Accessed 9/7/24 | Portuguese | **Yes**  **Full Version (shortened)**  **Portuguese** |
| **Prescott G** | **2012** | Prescott G, Venci J, Bednarzyk E. An advanced pharmacy practice experience (APPE) in spirituality and ethics at a secular school. *Christianity & Pharmacy.* 2012;15(1):13-16.  <https://www.cpfi.org/assets/docs/c-and-p/2012/cp_2012v15_1_p13-16.pdf>. | Accessed 1/11/25 |  |  |
| **Prestes C** | **2015** | Prestes, C. “Religión Como Tratamiento Complementario, Posibles Consecuencias En La Terapéutica. Revisión.  <https://revistas.um.edu.uy/index.php/revistahumanidades/article/view/179/157> | Accessed 9/7/24 | Spanish | Brief translation  Spanish |
| **Puchalski CM** | **2010** | [Puchalski, C., et al. “The Spiritual History: An Essential Element of Patient-Centred Care.” Spiritual Assessment in …, //, https://books.google.com/books?hl=en&lr=&id=7SK7qbvU1hIC&oi=fnd&pg=PA79&ots=ak2pQq9_zt&sig=NtHmRWhH4LZaHKnWiGrcb6ARGis.](https://books.google.com/books?hl=en&lr=&id=7SK7qbvU1hIC&oi=fnd&pg=PA79&ots=ak2pQq9_zt&sig=NtHmRWhH4LZaHKnWiGrcb6ARGis) | Accessed 9/20/24 |  |  |
| **Pullen L** | **2015** | [Pullen, L., et al. “The Relevance of Spirituality to Nursing Practice and Education.” Mental Health Practice, //, https://doi.org/10.7748/mhp.18.5.14.e916.](https://doi.org/10.7748/mhp.18.5.14.e916) | Accessed 9/20/24 |  |  |
| **Purnell MC** | **2019** | [*Purnell, MC, MS Johnson, R Jones, EB Calloway, DA Hammond, LA Hall, and DC Spadaro. “Spirituality and Religiosity of Pharmacy Students.” AMERICAN JOURNAL OF PHARMACEUTICAL EDUCATION 83, no. 1 (2019).*](https://www.researchgate.net/publication/331440422_Spirituality_and_Religiosity_of_Pharmacy_Students) | *Accessed 9/20/24* |  |  |
| **Querrey C** | **2020** | [Querrey, C. THE IMPORTANCE OF AND METHODS FOR EFFECTIVELY ADDRESSING SPIRITUALITY IN HEALTHCARE. Query date: 2023-06-28 16:19:16. repository.arizona.edu, 2020. https://repository.arizona.edu/handle/10150/651385.](https://repository.arizona.edu/handle/10150/651385) | Accessed 9/20/24 |  |  |
| **Quest TE** | **2006** | [Quest, TE, and NM Franks. “Vulnerable Populations: Cultural and Spiritual Direction.” EMERGENCY MEDICINE CLINICS OF NORTH AMERICA, vol. 24, no. 3, Aug. 2006, pp. 687-+, https://doi.org/10.1016/j.emc.2006.05.010. WOS:000239911600012.](https://doi.org/10.1016/j.emc.2006.05.010) | Accessed 9/21/24 |  |  |
| **Raffay J** | **2013** | [Raffay, J. “How Staff and Patient Experience Shapes Our Perception of Spiritual Care in a Psychiatric Setting.” JOURNAL OF NURSING MANAGEMENT, vol. 22, no. 7, Oct. 2014, pp. 940–50, https://doi.org/10.1111/jonm.12056. WOS:000343756300013.](https://doi.org/10.1111/jonm.12056) | Accessed 9/20/24 |  |  |
| **Ravenscroft P** | **2004** | [Ravenscroft, P., and E. Ravenscroft. “Spirituality and Surgery.” Surgical Palliative Care, //. https://books.google.com/books?hl=en&lr=&id=ydt1DwAAQBAJ&oi=fnd&pg=PA65&ots=dsqLzcY8IT&sig=aM6YX3kEQ4jwVG_FbG7GEBwH9QQ http://ndl.ethernet.edu.et/bitstream/123456789/36321/1/3053.pdf#page=78.](https://books.google.com/books?hl=en&lr=&id=ydt1DwAAQBAJ&oi=fnd&pg=PA65&ots=dsqLzcY8IT&sig=aM6YX3kEQ4jwVG_FbG7GEBwH9QQ%20http://ndl.ethernet.edu.et/bitstream/123456789/36321/1/3053.pdf#page=78) | Accessed 9/20/24 |  |  |
| **Rehn J** | **2019** | [Rehn, J. “Spiritualität Als Bedeutender Recovery-Faktor Bei Psychisch-Seelischen Erkrankungen Unter Besonderer Beachtung Transkultureller Behandlungsmethoden,” //. https://opus4.kobv.de/opus4-euv/files/810/Rehn_Julia.pdf.](https://opus4.kobv.de/opus4-euv/files/810/Rehn_Julia.pdf) | Accessed 9/7/24 | German | No |
| **Reis L** | **2007** | Reis LM, Baumiller R, Scrivener W, Yager G, Warren NS. Spiritual assessment in genetic counseling. *J Genet Counsel*. 2007;16: 41-52. <https://doi.org/10.1007/s10897-006-9041-8> | Accessed 9/20/24 |  |  |
| **Richardson P** | **2014** | [Richardson, P. “Spirituality, Religion and Palliative Care.” Ann Palliat Med, //. https://cdn.amegroups.cn/journals/amepc/files/journals/8/articles/4175/public/4175-PB9-R2.pdf.](https://cdn.amegroups.cn/journals/amepc/files/journals/8/articles/4175/public/4175-PB9-R2.pdf) | Accessed 9/20/24 |  |  |
| **Riley H** | **2020** | [Riley, H., and C. S. Officer. “Innovation to Transform Nursing Home Care.” Soulrapha.Org, https://www.soulrapha.org/s/SoulRapha_Research_Paper.pdf.](https://www.soulrapha.org/s/SoulRapha_Research_Paper.pdf) | Accessed 9/20/24 |  |  |
| **Rivera-Ledesma A** | **2007** | Ledesma, A. R., and M. M. L. Lena. “Ejercicio Clínico y Espiritualidad.” Anales de Psicología  <https://revistas.um.es/analesps/article/view/23111>  https://revistas.um.es/analesps/article/view/23111/22391 | Accessed 9/7/24 | Spanish | Brief translation  Spanish |
| **Roberts D** | **2019** | [Roberts, D. “From the Trainer: Spiritual Health: It’s Not Just for the Dying.” InnovAiT, //, https://doi.org/10.1177/1755738019854478.](https://doi.org/10.1177/1755738019854478) | Accessed 9/20/24 |  |  |
| **Robinson DE** | **2012** | [Robinson, D. E. Pastoral Care: A New Model for Assessing the Spiritual Needs of Hospitalized Patients. search.proquest.com, 2012, https://search.proquest.com/openview/04401b1fce0d6915713b616588f6b95f/1?pq-origsite=gscholar&cbl=18750.](https://search.proquest.com/openview/04401b1fce0d6915713b616588f6b95f/1?pq-origsite=gscholar&cbl=18750) | Accessed 9/20/24 |  |  |
| **Robinson KA** | **2017** | [Robinson, KA, et al. “Religious and Spiritual Beliefs of Physicians.” JOURNAL OF RELIGION & HEALTH, vol. 56, no. 1, Feb. 2017, pp. 205–25, https://doi.org/10.1007/s10943-016-0233-8. WOS:000392290700018.](https://doi.org/10.1007/s10943-016-0233-8) | Accessed 9/20/24 |  |  |
| **Rogers M** | **2021** | [Rogers, M. “Introduction to Spirituality.” Spiritual Dimensions of Advanced Practice Nursing …, //, https://doi.org/10.1007/978-3-030-71464-2_1.](https://doi.org/10.1007/978-3-030-71464-2_1) | Accessed 9/20/24 |  |  |
| **Rogers M** | **2016** | [Rogers, M. Spiritual Dimensions of Advanced Nurse Practitioner Consultations in Primary Care through the Lens of Availability and Vulnerability. A Hermeneutic Enquiry. eprints.hud.ac.uk, 2016, http://eprints.hud.ac.uk/28469 http://eprints.hud.ac.uk/id/eprint/28469/1/FinalThesisMelanieRogersMay2016.pdf.](http://eprints.hud.ac.uk/28469%20http:/eprints.hud.ac.uk/id/eprint/28469/1/FinalThesisMelanieRogersMay2016.pdf) | Accessed 9/20/24  both links |  |  |
| **Rush C** | **2020** | [Rush, C., et al. “Spirituality/Religion and Pain.” … of Spirituality, Religion, and Mental Health, //, https://www.sciencedirect.com/science/article/pii/B9780128167663000100 https://dl.dinevasi.com/books/2.pdf#page=204.](https://dl.dinevasi.com/books/2.pdf#page=204) | Accessed 9/21/24  both links |  |  |
| **Ruthes VRM** | **2019** | [Ruthes, VRM. “Integration of Spirituality in Health Care: Theoretical-Epistemological Considerations.” PERSPECTIVA TEOLOGICA, vol. 51, no. 3, Sept. 2019, pp. 481–502, https://doi.org/10.20911/21768757v51n3p481/2019. WOS:000504855700006.](https://doi.org/10.20911/21768757v51n3p481/2019) | Accessed 9/7/24 | Portuguese | Brief translation Portuguese |
| **Sagaser KG** | **2016** | [Sagaser, KG, SS Hashmi, RD Carter, J Lemons, H Mendez-Figueroa, S Nassef, B Peery, and CN Singletary. “Spiritual Exploration in the Prenatal Genetic Counseling Session.” JOURNAL OF GENETIC COUNSELING 25, no. 5 (October 2016): 923–35. https://doi.org/10.1007/s10897-015-9920-y.](https://doi.org/10.1007/s10897-015-9920-y) | Accessed 9/20/24 |  |  |
| **Sagberg S** | **2011** | Sagberg S, Roen I. Social practices of encountering death: A discussion of spiritual health in grief and the significance of worldview. International J of Childrens Spirituality. 2011;16(4):347-60.  <https://doi.org/10.1080/1364436X.2011.642854> | Accessed 9/20/24 |  |  |
| **Saguil A** | **2012** | [*Saguil, A., and K. Phelps. “The Spiritual Assessment.” AMERICAN FAMILY PHYSICIAN, vol. 86, no. 6, Sept. 2012, pp. 546–50. WOS:000308849600009.*](https://pubmed.ncbi.nlm.nih.gov/23062046/) | Accessed 9/20/24 |  |  |
| **Sandor MK** | **2006** | Sandor, MK, VS Sierpina, HV Vanderpool, and SV Owen. “Spirituality and Clinical Care: Exploring Developmental Changes in Nursing and Medical Students.” EXPLORE-THE JOURNAL OF SCIENCE AND HEALING 2, no. 1 (January 2006): 37–42. [*https://doi.org/10.1016/j.explore.2005.10.009*](https://doi.org/10.1016/j.explore.2005.10.009)*.* | Accessed 1/11/25 |  |  |
| **Saunders D** | **2020** | [Saunders, D., et al. “Varieties of Religious (Non) Affiliation: A Primer for Mental Health Practitioners on the ‘Spiritual but Not Religious’ and the ‘Nones.’” The Journal of …, //, https://journals.lww.com/jonmd/Fulltext/2020/05000/Varieties_of_Religious__Non_Affiliation__A_Primer.10.aspx?context=LatestArticles.](https://journals.lww.com/jonmd/Fulltext/2020/05000/Varieties_of_Religious__Non_Affiliation__A_Primer.10.aspx?context=LatestArticles) | Accessed 9/20/24 |  |  |
| **Seth SG** | **2010** | Seth SG, Goka T, Harbison A, Hollier L, Peterson S, Ramondetta L, Noblin SJ. Exploring the Role of Religiosity and Spirituality in Amniocentesis Decision-Making Among Latinas. *J Genet Counsel*. 201; 20:660-673.  <https://doi.org/10.1007/s10897-011-9378-5> | Accessed 1/11/25 |  |  |
| **Sheiff DS** | **2020** | [Sheriff, D. S. “The Eleventh International Public Health &Bioethics Ambassador Conference (IPHA11).” Researchgate.Net, n.d. https://www.researchgate.net/profile/Dhastagir-Sheriff/publication/345359593_Spiritual_Health_Care_and_Mental_Health_Issues_during_COVID_19_pandemic/links/5fa4f2db92851cc28698e82a/Spiritual-Health-Care-and-Mental-Health-Issues-during-COVID-19-pandemic.pdf.](https://www.researchgate.net/profile/Dhastagir-Sheriff/publication/345359593_Spiritual_Health_Care_and_Mental_Health_Issues_during_COVID_19_pandemic/links/5fa4f2db92851cc28698e82a/Spiritual-Health-Care-and-Mental-Health-Issues-during-COVID-19-pandemic.pdf) | Accessed 9/20/24 |  |  |
| **Shek DTL** | **2012** | [Shek, D. T. L. “A survey of the Literature Shows That Various Definitions of Spirituality Have Been Put Forward by Different Researchers. Based on Content Analyses of 31 Definitions of ….” The Oxford Handbook of Chinese Psychology, //, https://books.google.com/books?hl=en&lr=&id=5lNi6CZEhzoC&oi=fnd&pg=PA343&ots=QcUMZyFW9L&sig=PlwWW0LpejFYJt6DDnv9qbG87GA.](https://books.google.com/books?hl=en&lr=&id=5lNi6CZEhzoC&oi=fnd&pg=PA343&ots=QcUMZyFW9L&sig=PlwWW0LpejFYJt6DDnv9qbG87GA) | Accessed 9/20/24 |  |  |
| **Sichitiu CR** | **2011** | [Sichitiu, C. R., and T. Mulligan. “Spirituality as an Adjunct to Pain Management.” Handbook of Pain Relief in Older Adults: An …, //. https://doi.org/10.1007/978-1-60761-618-4_5.](https://doi.org/10.1007/978-1-60761-618-4_5) | Accessed 9/20/24 |  |  |
| **Siler S** | **2019** | [Siler, S., et al. “Interprofessional Perspectives on Providing Spiritual Care for Patients With Lung Cancer in Outpatient Settings.” ONCOLOGY NURSING FORUM, vol. 46, no. 1, Jan. 2019, pp. 49–58, https://doi.org/10.1188/19.ONF.49-58. WOS:000461139200008.](https://doi.org/10.1188/19.ONF.49-58) | Accessed 9/21/24 |  |  |
| **Skalla KA** | **2015** | [Skalla, KA, and B. Ferrell. “Challenges in Assessing Spiritual Distress in Survivors of Cancer.” CLINICAL JOURNAL OF ONCOLOGY NURSING, vol. 19, no. 1, Feb. 2015, pp. 99–104, https://doi.org/10.1188/15.CJON.99-104. WOS:000352448300019.](https://doi.org/10.1188/15.CJON.99-104) | Accessed 9/21/24 |  |  |
| **So H** | **2023** | [So, HT, L Mackenzie, C Chapparo, J Ranka, and MA McColl. “Spirituality in Australian Health Professional Practice: A Scoping Review and Qualitative Synthesis of Findings.” JOURNAL OF RELIGION & HEALTH, June 12, 2023. https://doi.org/10.1007/s10943-023-01840-5.](https://doi.org/10.1007/s10943-023-01840-5) | Accessed 9/21/24 |  |  |
| **Stackhouse JR** | **2003** | [Stackhouse, J. R. The Appropriate Clinical Response to Patient Suffering. Query date: 2023-06-28 12:26:39. search.proquest.com, 2003. https://search.proquest.com/openview/b48f3216e259257fdf853577730d47ce/1?pq-origsite=gscholar&cbl=18750&diss=y.](https://search.proquest.com/openview/b48f3216e259257fdf853577730d47ce/1?pq-origsite=gscholar&cbl=18750&diss=y) | Accessed9/21/24 |  |  |
| **Steven J** | **2012** | [Steven, J., M. E. Nelson, V. Barnet, and M. C. Brannigan. The Essential Guide to Religious Traditions and Spirituality for Health Care Providers. Query date: 2023-06-28 16:19:16. books.google.com, 2012. https://books.google.com/books?hl=en&lr=&id=39SlDwAAQBAJ&oi=fnd&pg=PT11&ots=R8zK9t_Uel&sig=I6HVGp9g7UNab9SqOMCrduN1JQ8.](https://books.google.com/books?hl=en&lr=&id=39SlDwAAQBAJ&oi=fnd&pg=PT11&ots=R8zK9t_Uel&sig=I6HVGp9g7UNab9SqOMCrduN1JQ8) | Accessed 9/21/24 |  |  |
| **Stratton RG** | **2019** | Stratton RG. The difference in satisfaction of religious and nonreligious hospital patients who received chaplain interventions. 2019. Thesis.  Available from ProQuest Central; *ProQuest Dissertations & Theses Global*. (2303232499). Retrieved from <https://www.proquest.com/dissertations-theses/difference-satisfaction-religious-nonreligious/docview/2303232499/se-2>. | Accessed 9/21/24 |  |  |
| **Summers KM** | **2022** | [Summers, KM, et al. “Defining Infertility: A Qualitative Interview Study of Patients and Physicians.” JOURNAL OF REPRODUCTIVE AND INFANT PSYCHOLOGY, June 2023, https://doi.org/10.1080/02646838.2023.2221277. WOS:001002430200001.](https://doi.org/10.1080/02646838.2023.2221277) | Accessed 9/21/24 |  |  |
| **Thangathurai D** | **2010** | Thangathurai D. Spirituality of caretakers and end of life care. The Chicago School of Professional Psychology ProQuest Dissertations & Theses.  2010. 10280460. Publicly Available Content Database. (1939044110). <https://www.proquest.com/dissertations-theses/spirituality-caretakers-end-life-care/docview/1939044110/se-2> | Accessed 9/21/24 |  |  |
| **Thomas AJ** | **2022** | *Thomas, A. J. An Educational Program for Nurses to Incorporate Spiritual Care into Clinical Practice. //,* [*https://search.proquest.com/openview/2f53ff6f140d39e0b6af59648f5c4c9e/1?pq-origsite=gscholar&cbl=18750&diss=y*](https://search.proquest.com/openview/2f53ff6f140d39e0b6af59648f5c4c9e/1?pq-origsite=gscholar&cbl=18750&diss=y)*.* | Accessed 9/21/24 |  |  |
| **Thune-Boyle ICV** | **2004** | [Thuné-Boyle, I. C. V. “Religiousness and Spirituality in Coping with Cancer.” Psychological Aspects of Cancer: A Guide to Emotional …, //, https://doi.org/10.1007/978-3-030-85702-8_8.](https://doi.org/10.1007/978-3-030-85702-8_8) | Accessed 9/21/24 |  |  |
| **Tracy S** | **2017** | Tracy, S. “Integrating Spiritual Assessment and Care in the Relief and Remission of Pain.” PAIN MANAGEMENT NURSING, vol. 18, no. 1, Feb. 2017, pp. 1–2, https://doi.org/10.1016/j.pmn.2016.12.002. WOS:000394413300001. | Accessed 9/21/24 |  |  |
| **Tschida C** | **2012** | [Tschida, C. A. Spirituality in Nursing Homes: A Social Work Perspective. Query date: 2023-06-28 16:19:16. ir.stthomas.edu, 2012. https://ir.stthomas.edu/ssw_mstrp/117/ https://ir.stthomas.edu/cgi/viewcontent.cgi?article=1116&context=ssw_mstrp.](https://ir.stthomas.edu/ssw_mstrp/117/) | Accessed 9/21/24 |  |  |
| **Unosson L** | **2016** | Unosson, L. ” Vart Skulle Jag Annars Gå?” Hur Kristna Människor Har Upplevt Gud i Livets Svåra Perioder.  chrome-extension://efaidnbmnnnibpcajpcglclefindmkaj/https://gupea.ub.gu.se/bitstream/handle/2077/44456/gupea_2077_44456_1.pdf?sequence=1&isAllowed=y | Accessed 9/7/24 | Swedish | Brief translation  Swedish |
| **Vásquez G** | **2018** | Vásquez, G. Fernández, and O. M. Murillo Picado. Abordaje Integral de Los Cuidados Espirituales En Salud En Atención Primaria: Revisión Bibliográfica.  <https://repositorio.sibdi.ucr.ac.cr/items/8db1e8ff-63a5-4315-9577-49084023c076> | Accessed 9/7/24 | Spanish | **Yes**  **Long version**  **Spanish** |
| **Ventura S** | **2005** | [Ventura, S. H. A Middle-Range Theory to Guide the Promotion, Support, or Improvement of Spiritually-Sensitive Care in Hospital Settings. //, https://search.proquest.com/openview/2cdff06fd50a13785f88e3c349d06389/1?pq-origsite=gscholar&cbl=18750&diss=y.](https://search.proquest.com/openview/2cdff06fd50a13785f88e3c349d06389/1?pq-origsite=gscholar&cbl=18750&diss=y.) | Accessed 9/21/24 |  |  |
| **Viana HM** | **2022** | Viana, H. M. Telemedicina:“Cuidados Espirituais Aplicados No Fim de Vida” Numa Revisão Sistemática Integrativa Com Síntese Narrativa.  <https://ubibliorum.ubi.pt/handle/10400.6/12835> | Accessed 9/7/24 | Portuguese | Brief translation  Portuguese |
| **Wachholtz AB** | **2016** | [Wachholtz, AB, et al. “A Comprehensive Approach to the Patient at End of Life: Assessment of Multidimensional Suffering.” SOUTHERN MEDICAL JOURNAL, vol. 109, no. 4, Apr. 2016, pp. 200–06, https://doi.org/10.14423/SMJ.0000000000000439. WOS:000373282400002.](https://doi.org/10.14423/SMJ.0000000000000439) | Accessed 9/21/24 |  |  |
| **Wachholtz A** | **2016** | [Wachholtz, A., and C. Fitch. “Spiritual Dimensions of Pain and Suffering.” Handbook of Pain and Palliative Care …, //, https://doi.org/10.1007/978-3-319-95369-4_34.](https://doi.org/10.1007/978-3-319-95369-4_34) | Accessed 9/21/24 |  |  |
| **Wacholtz A** | **2019** | [Wachholtz, A., and C. E. Fitch. “Role of Religion and Spirituality in the Patient Pain Experience.” Deer’s Treatment of Pain: An Illustrated Guide for …, //, https://doi.org/10.1007/978-3-030-12281-2_14.](https://doi.org/10.1007/978-3-030-12281-2_14) | Accessed 9/21/24 |  |  |
| **Welch JC** | **2016** | Welch, J. C. “The Development of a New Model for Assessing African-American Spirituality in Palliative Care,” //. [https://search.proquest.com/openview/16ccff073e0ddbfbb7113bd58b571a48/1?pq-origsite=gscholar&cbl=18750.](https://search.proquest.com/openview/16ccff073e0ddbfbb7113bd58b571a48/1?pq-origsite=gscholar&cbl=18750) | Accessed 9/21/24 |  |  |
| **Whitehead IO** | **2022** | [Whitehead, I. O. “Do You Believe in God, Doctor?” InnovAiT, //, https://doi.org/10.1177/17557380221124958.](https://doi.org/10.1177/17557380221124958) | Accessed 9/21/24 |  |  |
| **Whitehead IO** | **2022** | Whitehead IO, Jagger C, Hanratty B. Discussing spiritual health in primary care and the HOPE tool—A mixed methods survey of GP views. *PLoS ONE.* 2022;17(11): e0276281. <https://doi.org/10.1371/journal.pone.0276281> | Accessed 9/21/24 |  |  |
| **Whitehouse E** | **2020** | [Whitehouse, E., and N. Dando. “Management of Cancer Pain in Primary, Secondary, and Palliative Care.” Chronic Pain Management in General and …, //, https://doi.org/10.1007/978-981-15-2933-7_26.](https://doi.org/10.1007/978-981-15-2933-7_26) | Accessed 9/21/24 |  |  |
| **Wijker DE** | **2014** | Wijker, D. D. E. Kan Ik u Ergens Mee Helpen? Vaststellen van Spirituele Zorgbehoeften Bij Patiënten Die Zijn Opgenomen in Een Algemeen Ziekenhuis  <https://studenttheses.uu.nl/handle/20.500.12932/18771> | Accessed 9/7/24 | Dutch | **Yes**  **Long version**  **Dutch** |
| **Wiltjer H** | **2019** | [Wiltjer, H. K. N., and N. Kendall. “Assessment of Older People 6: Assessing the Spiritual Domain.” Nursing Times, //, https://www.nursingtimes.net/roles/older-people-nurses-roles/assessment-older-people-6-assessing-spiritual-domain-09-09-2019/.](https://www.nursingtimes.net/roles/older-people-nurses-roles/assessment-older-people-6-assessing-spiritual-domain-09-09-2019/) | Accessed 9/21/24 |  |  |
| **Winiger D** | **2007** | *Winiger, D. Physicians’ Perceptions of the Chaplain’s Role in Critical Care. //,* [*https://place.asburyseminary.edu/cgi/viewcontent.cgi?article=1300&context=ecommonsatsdissertations.*](https://place.asburyseminary.edu/cgi/viewcontent.cgi?article=1300&context=ecommonsatsdissertations) | Accessed 9/21/24 |  |  |
| **Wordsworth HA** | **2021** | *Wordsworth, H. A. “Addressing Spiritual Needs in Faith Community Nursing.” Spiritual Needs in Research and Practice: The Spiritual …, //,* [*https://doi.org/10.1007/978-3-030-70139-0_30.*](https://doi.org/10.1007/978-3-030-70139-0_30) | Accessed 9/21/24 |  |  |
| **Wynne L** | **2013** | Wynne, “Spiritual Care at the End of Life.”Nursing Standard (through 2013). Nursing Standard. 28, 2, 41-45. Date of submission: June 14 2013; date of acceptance: July 3 2013. [https://search.proquest.com/openview/d2efa2b0e05ff7085fe38c7e3c59b518/1?pq-origsite=gscholar&cbl=30130.](https://search.proquest.com/openview/d2efa2b0e05ff7085fe38c7e3c59b518/1?pq-origsite=gscholar&cbl=30130) (obtained via Brown Library request) | Accessed 9/21/24 |  |  |
| **Wysocka MA** | **2023** | [Wysocka, M., et al. “Is There a Connection Between Spiritual Transcendence and Quality of Life? A Cross-Sectional Survey Study in Patients Under the End-of-Life Care.” JOURNAL OF PALLIATIVE CARE, vol. 38, no. 1, Jan. 2023, pp. 10–16, https://doi.org/10.1177/08258597211034642. WOS:000685847000001.](https://doi.org/10.1177/08258597211034642) | Accessed 9/21/24 |  |  |
| **Younas A** | **2016** | [Younas, A. “Spiritual Care and the Role of Advanced Practice Nurses.” Nurs Midwifery Stud, //, https://www.researchgate.net/profile/Ahtisham-Younas/publication/317261729_Spiritual_Care_and_the_Role_of_Advanced_Practice_Nurses/links/5ca1d126299bf11169548bea/Spiritual-Care-and-the-Role-of-Advanced-Practice-Nurses.pdf.](https://www.researchgate.net/profile/Ahtisham-Younas/publication/317261729_Spiritual_Care_and_the_Role_of_Advanced_Practice_Nurses/links/5ca1d126299bf11169548bea/Spiritual-Care-and-the-Role-of-Advanced-Practice-Nurses.pdf) | Accessed 9/21/24 |  |  |
| **Zauszniewski JA** | **2021** | [Zauszniewski, JA, et al. “Development and Testing of a Spiritual Resourcefulness Scale: Holistic Expansion in Operationalizing the Resourcefulness Construct.” JOURNAL OF HOLISTIC NURSING, vol. 40, no. 1, Mar. 2022, pp. 7–15, https://doi.org/10.1177/08980101211025370. WOS:000664687500001.](https://doi.org/10.1177/08980101211025370) | Accessed 9/21/24 |  |  |
