# Supplementary material for: Evaluation of the HOPE spiritual assessment model: a scoping review of international interest, applications and studies over 20+ years
Source: BMC Palliat Care. 2025 Jul 7;24:191. doi: 10.1186/s12904-025-01809-z (PMC12236049; doi:10.1186/s12904-025-01809-z)
Supplement: Supplementary file 2 — Supplementary Material 2 [file 12904_2025_1809_MOESM2_ESM.docx]

**Supplemental Table 2**

| **Criteria Applied to Assess Acceptability, Feasibility and Validity**  **of the HOPE model in Scoping Review Sources** | | | | |
| --- | --- | --- | --- | --- |
| Rationale:  Since the HOPE model is a flexible, clinical communication tool (not a quantitative questionnaire), validity criteria typically used for quantitative research purposes do not apply. Therefore, based on literature review, the following methods and criteria were used to determine whether articles discussing HOPE reflected evidence for acceptability, feasibility and some forms of validity.  Methods:   1. All researchers independently evaluated each article selected for in-depth review using the following criteria & worksheet 2. Researchers met for several group analysis meetings to discuss differences of opinions until consensus was reached. | | | | |
|  | **Criteria** | **Rating** | | |
|  |  | **Yes** | **No** | **Unsure** |
| **Acceptability** | (a) author’s expert opinion recommendation or selection for use/adaption of HOPE in their setting  OR  (b) feedback from or study of stakeholders (patients, learners, clinicians, etc) regarding HOPE |  |  |  |
| **Feasibility** | (a) successful use of the HOPE model in a particular setting  OR  (b) feedback from stakeholders or study of stakeholders regarding HOPE |  |  |  |
| **Face Validity** | (a) authors choose to present HOPE and/or recommend use, based on literature review  OR  (b) authors select HOPE for use or adaption for use in their intervention, study, etc |  |  |  |
| **Content Validity** | (a) authors explicitly compare HOPE questions to domains deemed important in the literature regarding spiritual history or spiritual assessment  OR  (b) authors are experts in the field and endorse specific content |  |  |  |
| **Validity as a Teaching Tool** | Authors successfully used the HOPE model with desired educational outcomes, based on objective measures |  |  |  |
| **Validity as a Clinical Tool** | Authors successfully used the HOPE model with the desired clinical outcome, based on objective measures |  |  |  |
| **Validity as a Qualitative Research Tool** | Authors have successfully utilized or adapted the HOPE model for use in a qualitative research study with the desired outcome (eg. as a semi-structured interview guide) |  |  |  |
| **Language Translation –Validity** | Authors have translated and tested the HOPE model in their setting |  |  |  |
